# Fluorescent naphthalimide boronates as theranostics: structural investigations, confocal fluorescence and multiphoton fluorescence lifetime imaging microscopy in living cells†

**DOI:** 10.1039/d3cb00112a

**Published:** 2023-09-18

**Authors:** Megan J. Green, Haobo Ge, Stephen E. Flower, Charareh Pourzand, Stanley W. Botchway, Hui-Chen Wang, Navaratnarajah Kuganathan, Gabriele Kociok-Köhn, Meng Li, Suying Xu, Tony D. James, Sofia I. Pascu

**Affiliations:** a Department of Chemistry, University of Bath Calverton Down Bath BA2 7AY UK syxu@mail.buct.edu.cn T.D.James@bath.ac.uk s.pascu@bath.ac.uk; b Department of Life Sciences, University of Bath BA2 7AY UK; c Centre for Therapeutic Innovation, University of Bath BA2 7AY UK; d STFC Research Complex at Harwell, Rutherford Appleton Laboratory, Harwell Science and Innovation Campus Harwell Oxfordshire OX11 0QX UK; e Department of Materials, Imperial College London London SW7 2AZ UK; f Materials and Chemical Characterisation Facility (MC2), University of Bath Calverton Down Bath BA2 7AY UK; g State Key Laboratory of Chemical Resource Engineering, Beijing University of Chemical Technology Beijing 100029 P. R. China; h Hebei Key Lab of Power Plant Flue Gas Multi-Pollutants Control, Department of Environmental Science and Engineering, North China Electric Power University Baoding 071003 P. R. China

## Abstract

New design and synthetic strategies were developed to generate functional phenyl boronic acid (BA)-based fluorescent probes incorporating the 1,8-naphthalimide (NI) tag. This fluorescent core was anchored onto the BA unit through small organic linkers consisting of nitrogen groups which can arrest, and internally stabilise the phenyl-boronate units. The newly synthesised fluorophores were characterised spectroscopically by NMR spectroscopy and mass spectrometry and evaluated for their ability to bind to a naturally occurring polysaccharide, β-d-glucan in DMSO and simultaneously as act as *in vitro* cell imaging reagents. The uptake of these new NI-boronic acid derivatives was studied living cancer cells (HeLa, PC-3) in the presence, and absence, of β-d-glucan. Time-correlated single-photon counting (TCSPC) of DMSO solutions and two-photon fluorescence-lifetime imaging microscopy (FLIM) techniques allowed an insight into the probes’ interaction with their environment. Their cellular uptake and distributions were imaged using laser scanning confocal fluorescence microscopy under single- and two-photon excitation regimes (*λ*_max_ 910 nm). FLIM facilitated the estimation of the impact of the probe's cellular surroundings using the fluorophore lifetime. The extent to which this was mediated by the β-d-glucan was visualised by 2-photon FLIM in living cells. The fluorescence lifetime observed under a range of temperatures varied appreciably, indicating that changes in the environment can be sensed by these probes. In all cases, the cellular membrane penetration of these new probes was remarkable even under variable temperature conditions and localisation was widely concentrated in the cellular cytoplasm, without specific organelle trapping: we conclude that these new probes show promise for cellular imaging in living cancer cells.

## Abstract 2

New design and synthetic strategies were developed to generate functional phenyl boronic acid-based fluorescent probes with a 1,8-naphthylimide bright core, and their beta-d-glucan hybrids. These were monitored by MP FLIM for their ability to report changes in their environments in live cells.

## Graphical abstract

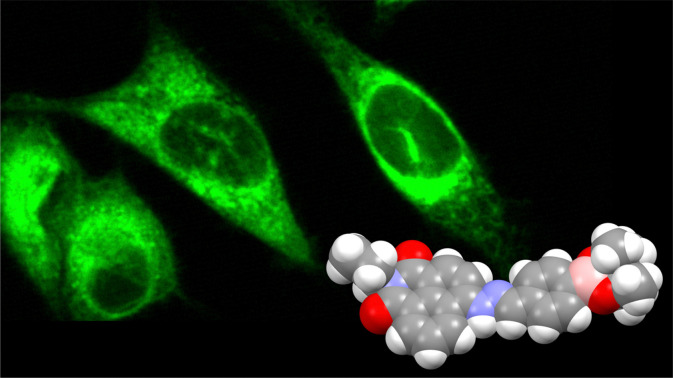

## 1. Introduction

Functional boronic acid-based molecules and materials have become increasingly relevant as components of the synthetic toolbox used for biological molecular sensing, organic optoelectronic materials assemblies, and high-performance gels, in bioimaging applications as well as in bio-nanotechnology building on from the established ability of boronic acids to recognise the diol functionalities of saccharides, *in vivo* and *in vitro*.^1^ James *et al.* have a long-standing interest in development of new functional boronic acids for the detection of monosaccharides and elucidated the mechanisms by which boronic acid derivatives can interact rapidly and reversibly with saccharides in aqueous media, of relevance to sensing and imaging. The processes by which they undergo competing dynamic mechanisms to exchange protecting diols for better matched substrates, under thermodynamic control, have been unravelled.^2,3^

**Scheme 1:**
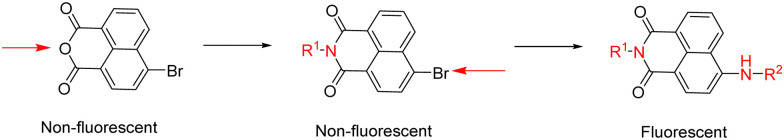
Functionalisation sites at the 1,8-naphthalimide (NI) core with potential to switch fluorescence emission on/off by inclusion of N-donors.

Naphthalimides (NIs) play an important role as synthetic scaffold for complex, functional materials, and as fluorescent dyes. Unlike common organic fluorophores incorporating pyrene (with emission *λ*_max_ 373–385 nm) and anthracene (emission *λ*_max_ 400–450 nm), the 1,8-naphthalimide core displays a fluorescence emission band centred around 500 nm and it is also versatile for extensive functionalisation on basis of two possible sites of substitution, which provide the flexibility for several conjugation pathways, opening possibilities for bioconjugation and applications at the life sciences interface. The unique structure of the naphthalimide core endows the resulting compounds with exceptional features, including strong electron affinity as well as strong thermal and oxidative resilience, which has sparked broad interest in their development and applications. Derivatization of differing positions at the NI core can drastically alter the photophysical properties due to substantial changes of the charge distribution along the conjugated backbone. Depending on the nature of substituents involved at the peripheral structure of 1,8-naphthalimide the on/off fluorescence switching can be triggered (Scheme 1). Taken together these characteristics render naphthalimides as adaptable fluorophores in the current sensing research ground.^4–9^ Overall, 1,8-naphthylimide performs as electron acceptor in donor–acceptor supramolecular systems. To advance the design of new sensing agents for biological processes *in vitro* that can simultaneously act as cellular imaging probes, the naphthalimide synthon acts as a bridge, between R1 and –NH–, a functional, electron-donating group that can further act as an aliphatic or aromatic linker, denoted R2 in Scheme 1. Depending on the nature of substitutions introduced at the 4-position of the naphthyl group through derivatisation of R1, the fluorescence emission signal may be tuneable. Based on these features, this scaffold has become a focus of interest to develop new fluorescent chemosensors for analytical chemistry applications such as the molecular recognition of bio-analytes and their molecular imaging, of relevance for biomedical processes in living systems. N-substituted-1,8-naphthalimides (NIs) and their widely studied congeners, naphthaldiimides (NDIs) have been shown to display remarkable photophysical and photochemical properties in aqueous environments and have been recognised as important Donor–π–Acceptor chromophores for biologically-relevant binding interactions.^10,11^ This uniquely positioned functional group, NI, is excitable with the commonly available laser lines for excitation at 405 or 488 nm in confocal fluorescence microscopy assays, and is also responsive to the corresponding 810 and 910 nm excitation in 2-photon excitation modes. In our previous studies, related compounds of the wider napthalimide (NI) and naphthaldiimide (NDI) family showed kinetic stability and biocompatibility in cellular environments, as well as ability to respond to changes in their environment, which was probed by multiphoton fluorescence lifetime imaging techniques.^12–16^ The flat and aromatic structures of NI/NDI and perylene based-imides renders them easily modifiable to give rise to supramolecular aggregates and excimers with strong absorption and fluorescence emissions ranging from the visible to near-IR region.^17^ Functional N-substituted-1,8-naphthalimide derivatives have been effectively pursued as desirable probes for DNA binding in solution and in living cells, and synthetic scaffolds for anticancer and cellular imaging agents.^18,19^

Unlike the naphthalene imides core,^20^ the monomeric naphthalene diimide is only weakly fluorescent and extensive core-derivatisation needs to be performed to enhance its fluorescence and thus wider usefulness. Interestingly perylene monoimide (PeIm)-conjugates led to new NIR probes for *in vitro* and *in vivo* bio-imaging,^21^ although there are solubility issues in aqueous media with both of these larger aromatic acceptor molecules with extended aromatic rings. In addition, the high reactivity of the boronate groups gives the NI-based dyes incorporating this recognition unit inherent cancer targeting properties, as reactions with glycosylated groups of proteins and other biomolecules in living cells can occur under physiological conditions.

In earlier bioanalytical sensing-geared research, James *et al.* discovered that the occurrence of an enhanced interaction between the neighbouring amine and the boron atom of a probe structurally related to compound 1. The enhanced N–B interaction hampered the reaction between boron and certain analytes (*e.g.* reactive oxygen species) in the presence of saccharides and these processes were carefully monitored by fluorescence quenching experiments in the presence and absence of a range of carbohydrates and under variable pH and solvent conditions.^22^

For example, the Trupp group designed a water-soluble probe (I) for saccharides by attaching the boronic acid moiety in the 4-position with an ethylene as a linker.^23^ By ‘borrowing’ the B–N interaction, pronounced spectral changes were observed by fluorescence spectroscopies with the addition of a range of saccharides. The sensor showed highest selectivity towards d-fructose with a fluorescence turn-on response to saccharides with a Stokes' shift of emission maximum of *ca.* 120 nm upon exposure to saccharides, a crucial advantage for the design construction of fluorescent sensors. Based on the design of probe I, the Wang and James groups reported several series of sensors synthesised by modifying the 4-amino group directly with functional phenylboronic acid groups and investigated the substitution effect on the saccharide sensing.^24,25^ Generally results indicated that the variation of the substitution only produced a limited effect on saccharide binding. By attaching the boronic acid group to the naphthalimide through a hydrazone bond, the degree of conjugation in the entire system would be expected to extend due to the “amino conjugation”,^26^ which, it was hypothesised, would facilitate the occurrence of a charge transfer interaction and therefore a change in the fluorescence emission with respect to the un-substituted NI unit.

Within this work, a set of new functional compounds based on the boron-containing 1,8-naphthalimide probe motifs were investigated. We synthesized a new library of imaging agents incorporating the NI-phenyl-boronic acid entity as a highly modular framework. As stated above, boronic acids (BA) are a class of receptors very well suited to the binding of carbohydrates. Therefore, we aimed to evaluate the cellular uptake of napthalamide-boronic acids conjugates in cells and compare their cellular activity with that of the known, and structurally related, NI-boronic acid sensor type III that shown remarkable cellular activity and sensing properties.^27^ Overall, such species showed promising biological compatibility in a range of cancer cells, and ability to act as sensors by virtue of their binding to either fluoride anions (in case of III, first reported by S.-Y. Xu *et al.*^27^) or Cu(ii) cations (in case of IV, first reported by M. Li *et al.*^33^) in biocompatible solvents. We showed earlier that the presence of a weakly bound pinacol protective group is necessary at the synthesis stage to prolong the kinetic stability of these boronic acids, yet this was shown to be easily removed under mass spectrometry conditions, under acidic conditions or in the presence of the competitive diol groups provided by competing carbohydrates, *e.g.* including those of the β-d-glucan and related species present on cellular membranes. Our previous experiments on boronic acid conjugates of established, commercial dyes such as coumarin^12^ and fluorescein^13^ and corresponding hybrids with β-d-glucan were highly successful as well as the use of florescence lifetime imaging microscopy (FLIM) to characterise such hybrids in cells. This inspired us to prepare the corresponding boronic acid conjugates of the naphthalimides 1–3 with β-d-glucans and investigate their cellular uptake as well as potential as theranostics.

Here we describe the novel boronate-based 4-amino-1,8-naphthalimide conjugates 1–3, which we found are possessing both desirable emission properties for cellular imaging applications and cellular cytotoxicity, which also enable us to probe the hypothesis that the presence of β-d-glucan mediates the cellular uptake and biolocalisation of napthalimide boronic acids and visualise this in living cancer cells using two-photon fluorescence lifetime imaging microscopy (2P FLIM).

## 2. Results and discussion

### 2.1. Synthesis and spectroscopic investigations

**Fig. 1:**
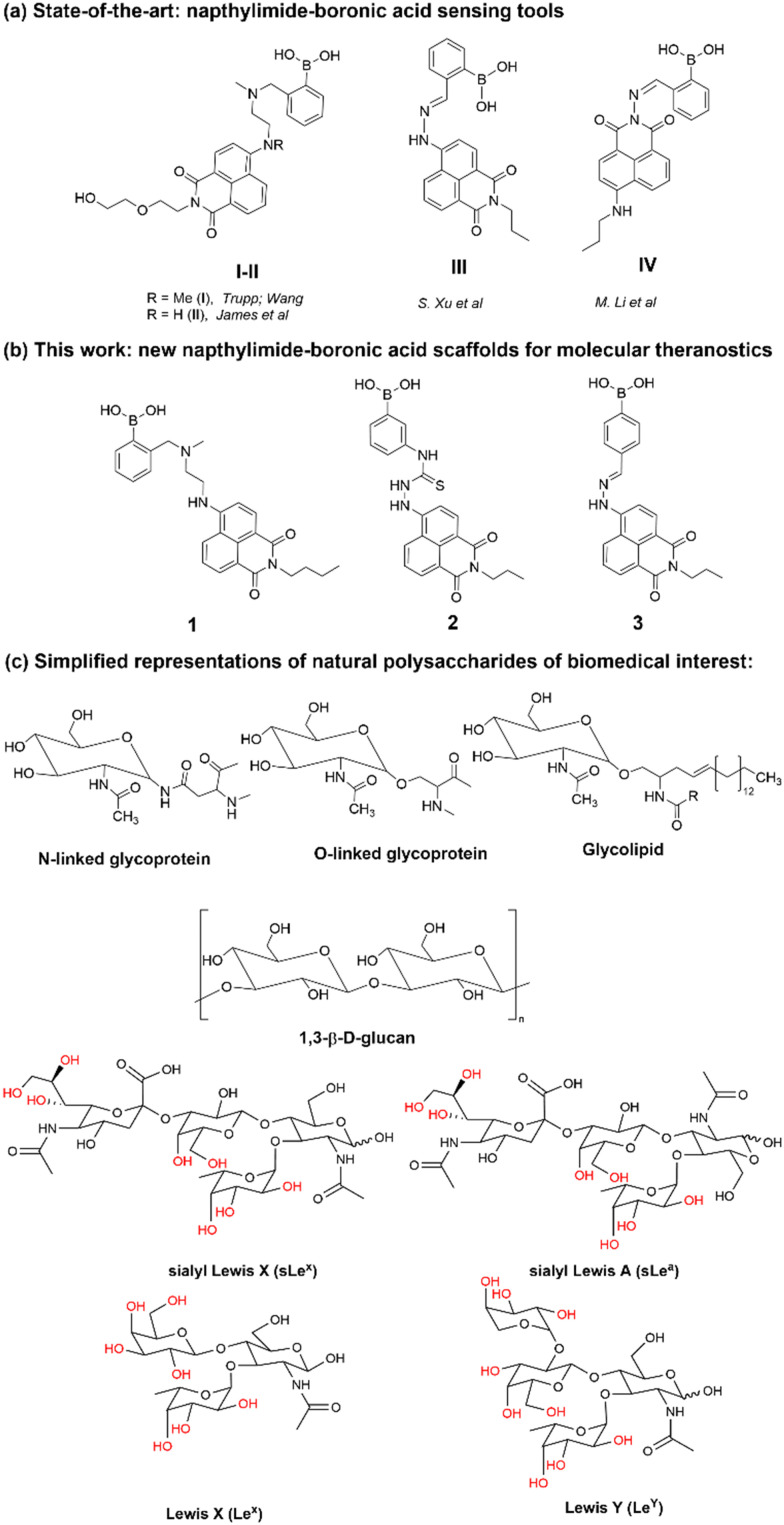
Schematic representations of: (a) previously reported NI-boronic acid sensors for the detection of analytes such as carbohydrates, denoted compounds I–IV; (b) new compounds 1–3 which were investigated here as BPin protected species and (c) fragments of the polysaccharides of interests for this investigation: β-d-glucan (naturally-occurring biopolymer incorporating glucose units as the monosaccharides monomers and a range of beta-1,3 main glycosidic linkages, from barley, [C_6_H_10_O_5_]_*n*_, *M*_W_ 30–650 × 10^3^ g mol^−1^, Aldrich, used hereby) and several related saccharide synthons commonly found in living cells.

**Scheme 2:**
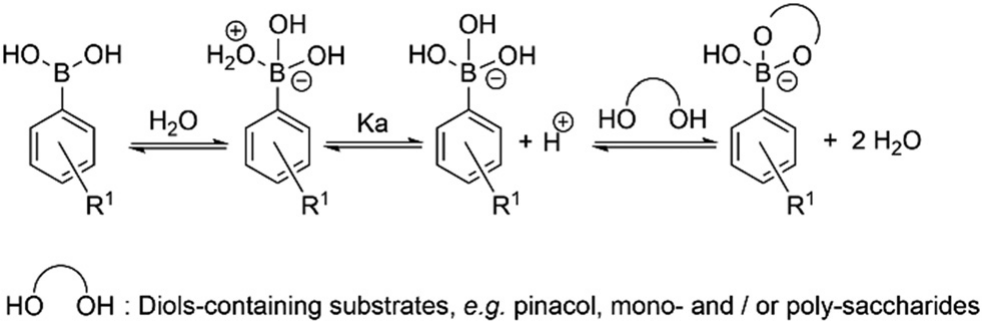
Schematic representation of the simple competing equilibria in aqueous environment involved at the recognition of vicinal diols by phenyl-boronic acid.

Synthesis and characterisation details for new compounds 1–3 (isolated as corresponding pinacol-borate derivatives) are given in ESI.† Whilst the multi-step synthesis of compound 1 was challenging especially in terms of its purification, compounds 2 and 3 were formed in moderate yield from straight forward protocols (using established procedures, see ESI†). For instance, the HRMS (ESMS) showed the expected isotopic distribution pattern matching that of the calculated one (ESI†) for all compounds 1–3. Compounds III and IV, depicted in Fig. 1, have both previously been developed in our laboratories as carbohydrate sensors and were employed herein for comparison studies. A comparison of the solution behaviour of compound 3 (synthesised and characterised as described in ESI†) and the known compound III, resynthesised here as a BPin protected analogue. Selected examples of functionalization reactions involving 1,8-napthliminde-based boronic acid functionalized dyes are given in the ESI,† alongside computational approaches (DFT) to geometry prediction of compound 3. Compound III (our earlier-reported, *ortho*-substituted, analogue of 3,^27^) was re-synthesised hereby through an improved methodology, because it showed an extremely high fluorescence emission in DMSO, which was traceable in cells at less than 2 μM concentrations: this indicated that these compounds generally have a poor solubility in aqueous environment and precipitate from pure water and mostly water-based buffers, yet they are highly soluble in DMSO. The excitation and emission spectra of pinacol-protected 1–3 were recorded at room temperature in DMSO (see ESI†) and showed the expected features for this class of compounds. The absorption spectra of 1–3 showed bands centred at 350 nm, assigned to the p–p* transitions of the phenyl substituents. A strong absorption band centred at *λ*_max_ 450 nm was observed in each case, as expected, and assigned to the internal charge transfer (ICT) transition that dominates the absorption spectroscopy of compounds incorporating the 4-amino-1,8-naphthalimide unit. Excitation of 1–3 at *λ*_max_ 488 nm resulted in a broad emission band centred at 580 nm in each case (with the characteristically low quantum yields, less than 0.005). Pinacol-protected compounds III-BPin and 3-BPin remained stable in buffer systems with water content of minimum 17%, as shown by monitoring fluorescence emission intensity changes over time in methanol–water co-solvent buffer system (ESI†). It is well known that the interaction affinity between boronic acid and diols are dependent on the pH values of the system. The boronic acid on compound III was synthesised as protected by a pinacol group therefore, competitive binding processes likely occur within this system upon treatment with (poly)saccharides, *e.g.* of the type shown in Fig. 1. The pH profile was therefore plotted by recording fluorescence intensities of compound III at different pH values with and without presence of saccharide (ESI†). The fluorescence emission intensity of compound III remained stable under slightly acidic conditions and decreased along with increasing the pH of the system. The fluorescence was almost totally quenched at pH 11. It is well-known that the binding between boronic acid and saccharides generally induces the formation of boronate esters, which could easily convert to its anionic form, leading to the quenching of fluorescence, according to the schematic representation shown in Scheme 2. It has been shown that even the simplest BA receptors have affinity for carbohydrates under biologically relevant conditions, in aqueous media. The binding interaction between d-fructose and boronic acids is likely much stronger than that of pinacol with boronic acid moiety, which was shown in previous work to overwhelm the pinacol.^27^ The pH titration suggested that the occurrence of binding interactions with saccharides could result in the largest fluorescent difference at around pH 7.4. The boron-containing 1,8-naphthalimide probe III showed good response for saccharide detection. Additionally, kinetic stability and pH optimisation tests were carried out and it was found that the pinacol-protected compound III showed observable spectral changes at pH 7.4 with 17 vol% water content. Fluorescent titrations with different monosaccharides revealed that the emission intensity of compound III was reduced, and the degree of quenching follows the trend: d-fructose > d-galactose > d-mannose > d-glucose. These tests with compound III showed the expected saccharide binding interactions and these, together with kinetic stabilities tests for compounds in the series are given in ESI† (Fig. S33–S45). The fluorescence measurements on these naphthalimide based boronic acid sensors have indicated low sensitivity upon addition of β-d-glucan. In cases of the (pinacol-protected) compounds studied here, the presence of excess of β-d-glucan led to minor quenching of fluorescence emission and only up to 5 nm red-shift of the fluorescence emission. This is in line with previous observations in binding studies undertaken for boronic acid conjugates of coumarin– and fluorescein–boronic acids which also showed interactions with these saccharides as well as β-d-glucan, and where fitting isotherms at the titrations gave association constants^12,13^ in the range of 10^4^ to 10^6^ M^−1^ yet no binding constant could be determined for this interaction, likely due to multiple equilibria present in solution. Due to the bifunctional nature of 1–3, and the strong similarities observed with the structurally analogues compounds III–IV, we anticipated that the presence of the boronate groups anchored onto the 4-amino-1,8-naphthalimide unit would modulate the interactions between these compounds and polysaccharides such as β-d-glucan. This was implemented here for its potential to act as an adjuvant, to facilitate cellular uptake and enhance the biocompatibility of compounds.^12,13^ Such 1,3-β-d-glucans are glucopyranose polysaccharides with (1,3) glycosidic linkages with varying degree of (1,4) and (1,6) branches found in the cell walls of yeasts and plants such as oats and barley We also considered that this polysaccharide as a model biopolymer closely related to those found in cancer cells, as shown in Fig. 1. Therefore, formulations with excess of β-d-glucan were prepared for 1–3 (according to our established protocols and ESI†), this approach aimed to improve the biocompatibility of the NI dye whilst retaining cellular penetration potential. Briefly, a stock solution of the NI compounds 1–3 as their BPin protected variants (10 mM, 1 mL) were dissolved in DMSO. The corresponding solutions were added to β-d-glucan (1 mg mL^−1^, 1 mL) in DMSO, and the pH was adjusted to 7.4 using a phosphate buffer solution and the mixture was stirred at room temperature overnight, in a vial was protected from light, and used directly as a stock solution for 2-photon TCSPC and cellular imaging experiments. This gave rise to the hybrids denoted‘compound@glucan’ and analysed in solution and in cellular environments (*vide infra*).

### 2.2. Molecular structure insights for 3-BPin and 3 from single crystals X-ray diffraction and DFT calculations

**Fig. 2:**
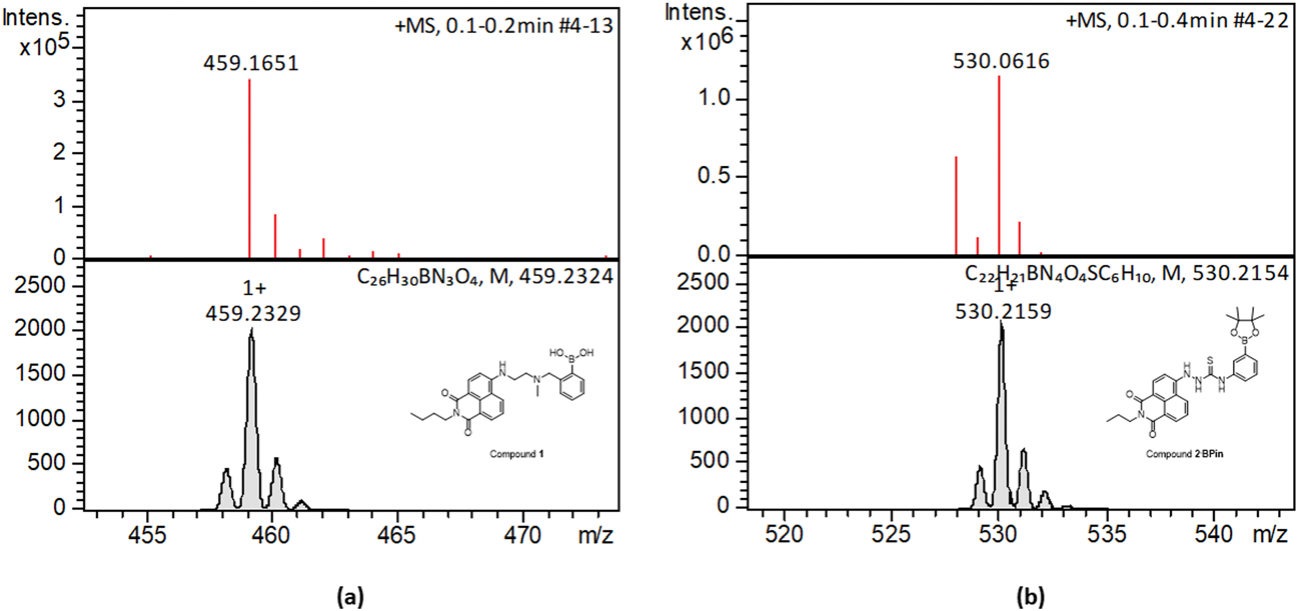
HR mass spectrometry (ESI^+^) and best fit isotopes match found at the analysis of compounds 1-Bpin (a) and compound 2-Bpin (b). The compound fragmentation and loss of pinacol group/deprotection was typically being observed during LC-MS ESI^+^ analysis of all compounds in this series. Further mass spectrometry data are given in ESI.†

**Fig. 3:**
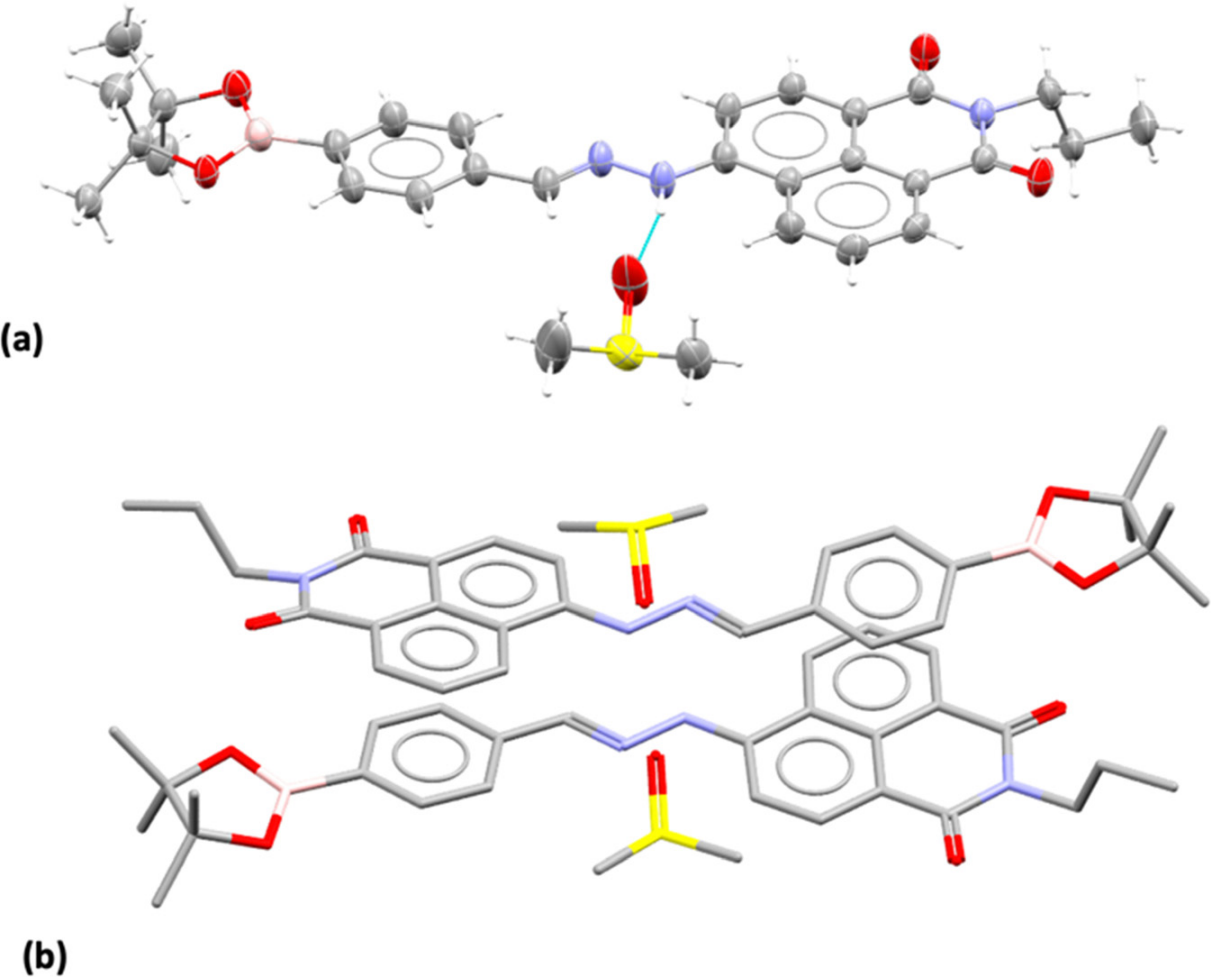
Single crystal X-ray structure analysis: (a) molecular structure of compound 3 showing the presence of H-bonded DMSO molecule. ORTEP representation (50% ellipsoids); (b) content of asymmetric unit indicated the presence of two almost identical pinacol-protected NI units of compound 3, in a head-to-tail aromatic stacked configuration with associated DMSO molecules (H's were excluded for clarity). Molecular parameters from the solid state characterisation and their comparison with the gas-phase DFT calculations are given in the ESI,† colours: C: grey, S: yellow, B: pink, N: blue, O: red, H: white.

Compound 3, isolated from analogous precursors with those employed in the synthesis of compounds 1–2 (with selected characterisation data given in Fig. 2 and ESI†) was fully characterised structurally as the pinacol-protected derivative, 3-BPin (Fig. 3). Single, X-ray quality, crystals of 3-BPin were grown from a 10 mM concentration DMSO solution at room temperature, over several weeks. Compound 3-BPin crystallised in the monoclinic space group *P*2_1_/*c*, with two identical molecules (aromatically stacked and positioned head-to-tail) and further two interstitial DMSO molecules, H-bonded to the linking NH groups in the asymmetric unit. Experimental details are given in ESI,† and crystal structure analysis data was deposited to CCDC database (2278161). In the unit cell extended aromatic stacking interactions were observed between the phenyl-boronate moieties and the 4-amino-1,8-naphthalimide rings of the neighbouring molecules, with centroid–centroid distances of *ca.* 3.5 Å.

Gas-phase DFT calculations on compound 3-BPin and deprotected variant 3 were performed to shed light into the electronic structures of these species. The resulting relaxed structure of naphthalimide boronate with pinacol-protected boronic acid group is shown in Fig. 5a whereas Fig. 5b shows the relaxed structure of the naphthalimide boronate featuring an unprotected boronic acid group. Density functional theory (DFT) calculations as implemented in the Gaussian 09 package^28^ were used to examine the structures, estimate the Mulliken charges on each atoms in the relaxed structures and construct molecular orbitals to predict the band gap that associated with the degree of charge transfer. The exchange correlation energy was modelled using the schemed parameterised by Perdew, Burke and Ernzerhof Perdew (PBE). The PBEPBE exchange correlation functional and 6-31G** basis sets were used for all atoms (B, C, N, O and H) as implemented in the relevant code.^29^ Mulliken analysis^30^ was used to estimate the charges on the atoms in the relaxed structures and molecular orbital diagrams (Fig. 5c) were constructed using GaussView 6.1.1.^31^

DFT calculations (gas-phase approximation) showed that there is a good agreement between experimental structural parameters from the X-ray diffraction structure and those in the optimised structures (ESI†).

**Fig. 4:**
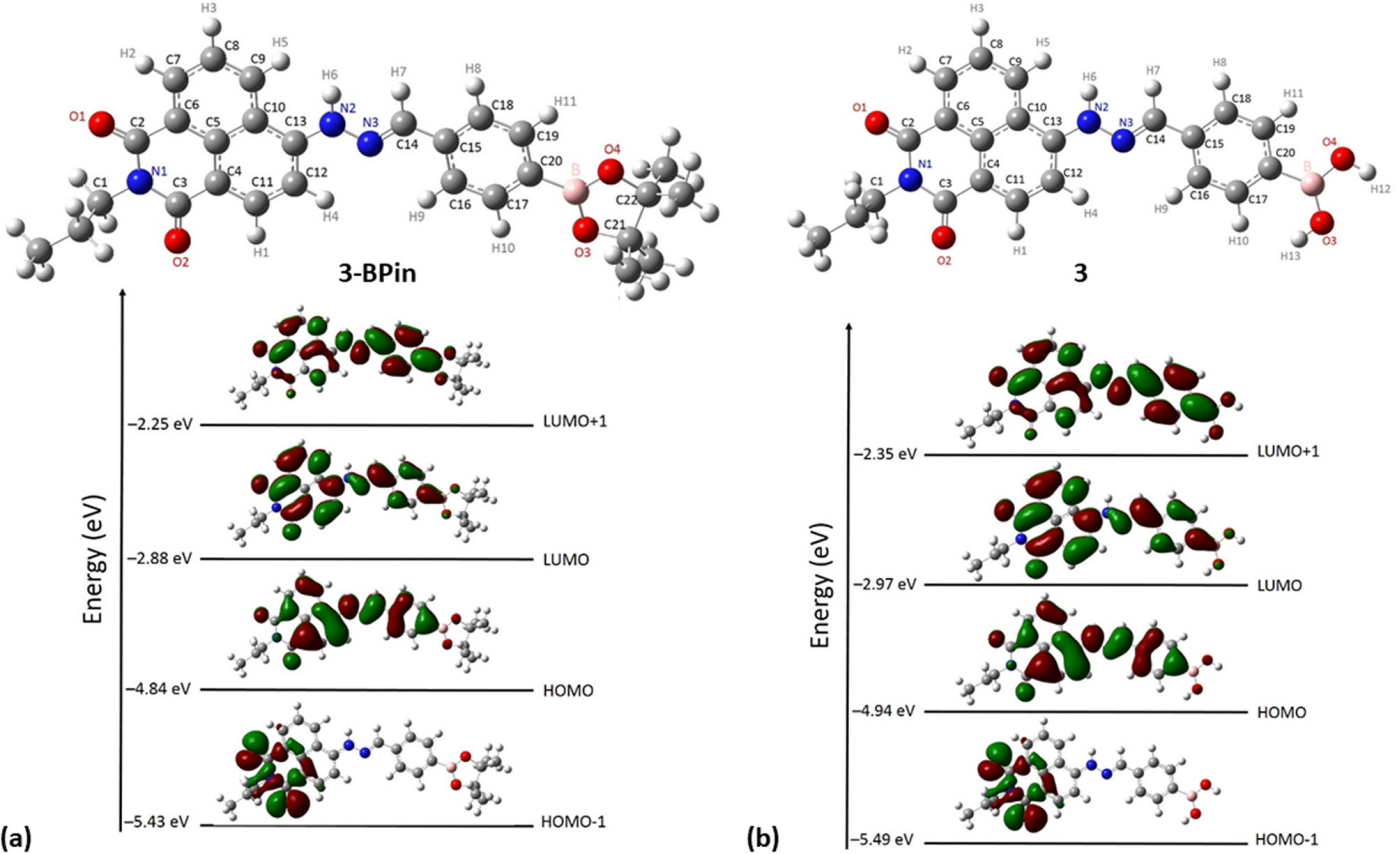
DFT optimisations (gas phase): (a) relaxed structure of naphthalimide boronate with pinacol-protected boronic acid group, 3-BPin, and corresponding DFT calculated shapes and energies of frontier orbitals (b) relaxed structure of the deprotected naphathalimide boronate naphthalimide boronic acid 3 and corresponding DFT calculated shapes and energies of frontier orbitals.

The Mulliken charge analysis shows that N and O are partially negatively charged as expected due to their high electronegativities. The charge on the boron atom in the boronic acid group is +0.433 and its nearest neighbour O atoms are negatively charged (see ESI†) showing the large electronegativity difference between the B and the O. The HOMO–LUMO gap for the modelled (pinacol-protected) compound 3-BPin was calculated to be 1.96 eV (see Fig. 4). From the inspection of the modelled compound 3 (featuring an unprotected boronic acid group) it seems that the removal of pinacol group has a very little effect on the bond lengths (see Tables S5, S6 and S8 in ESI†) whilst the bond angles in the boronic acid unit are slightly altered possibly due to the absence of the bulky methyl groups attached to the pinacol group. The Bader charge on the B is slightly high as the nearest neighbour O atoms become more negatively charged (see ESI†). The larger negative charges on the O atoms may be due to the H atoms gaining more electrons than the C atoms in the pinacol groups. The HOMO–LUMO gap (1.97 eV) is almost unaltered in the absence of pinacol group (see Fig. 4), although it is evident that the energy of each of the frontier orbitals is lowered by corresponding 0.1 eV in modelled species 3*vs.* its pinacol-protected analogue, 3-BPin. We assigned this to the consequences of the removal of the steric bulk and some internal stabilisation of the B(OH)_2_ groups, as expected, occurring in compound 3.

**Fig. 5:**
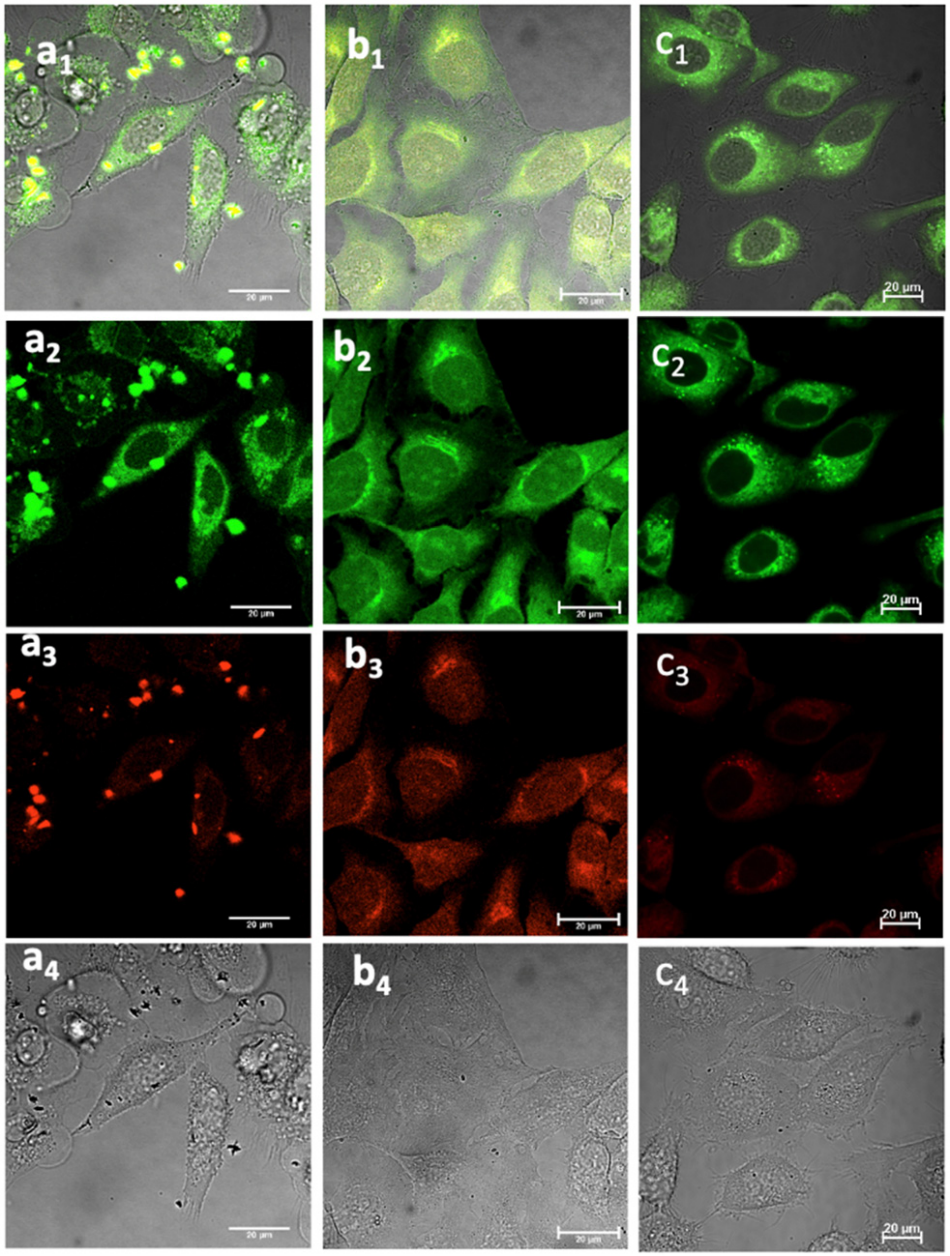
Laser confocal microscopy of live cells incubated with NI-based compounds under various conditions, with *λ*_ex_ 488 nm: (a) Compound 1-BPin, 100 μM (in 1 : 99% DMSO: RPMI) in HeLa cells at 37 °C, 15 min incubation imaged before PBS washing; (b) 1@glucan in HeLa cells, at 37 °C; (obtained *in situ* by mixing 100 μM of 1-BPin and 10 μg mL^−1^ in glucan in 0.5 : 99.5% DMSO: RPMI); (c) Deprotected Compound 1 in PC-3 cells, 1 μM concentration, 20 minutes incubation at 37 °C; (a_1_)–(c_1_) overlay images of the green channel, red channel and DIC; (a_2_)–(c_2_) *λ*_em_ 516–530 nm (green channel); (a_3_)–(c_3_) *λ*_em_ 615–650 nm (red channel); (a_4_)–(c_4_) is the DIC channel. ESI,† shows additional imaging micrographs.

### 2.3. Cellular uptake investigations in living cells

**Fig. 6:**
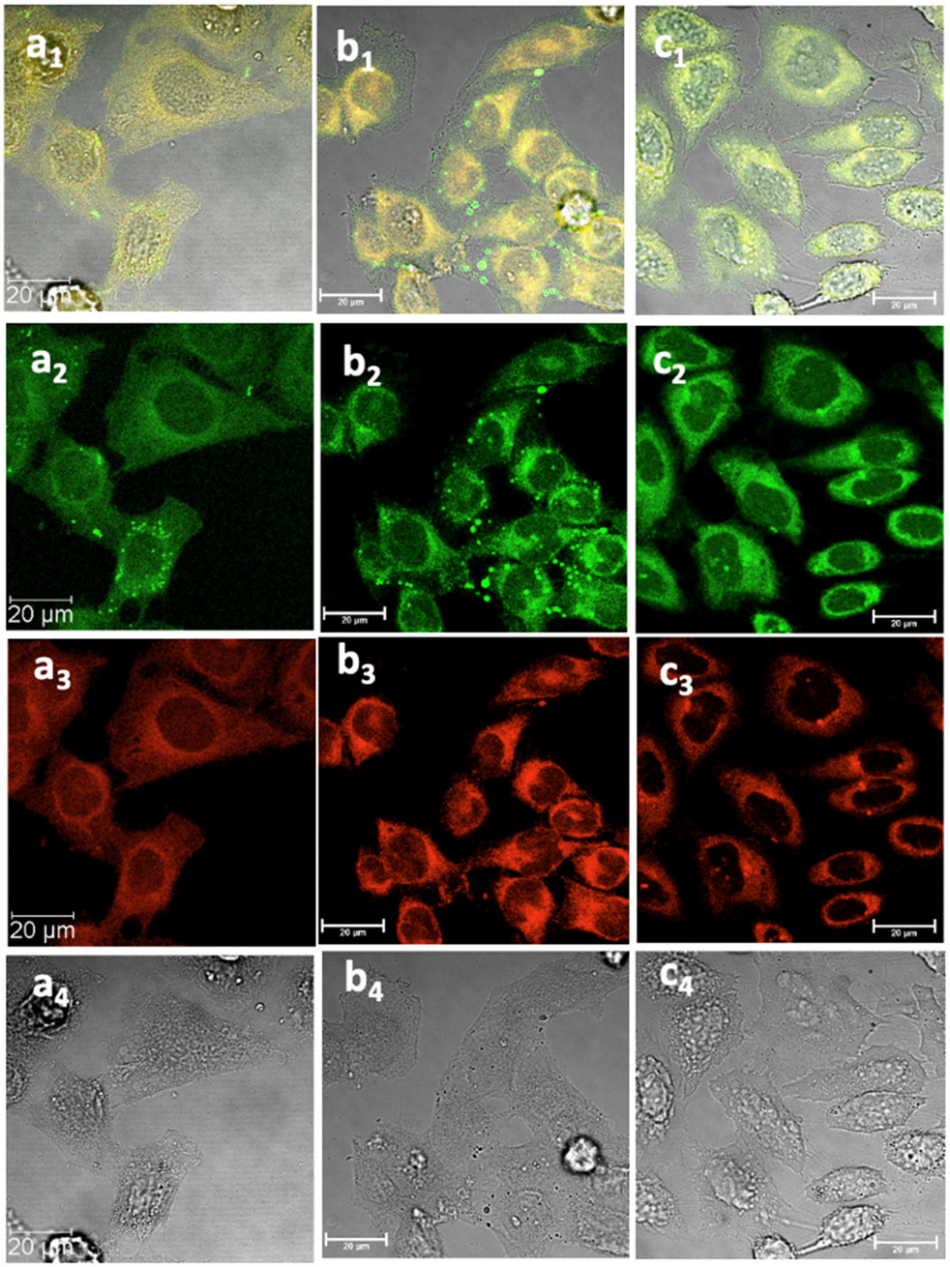
Laser confocal microscopy of live HeLa cells incubated with NI-based compounds in HeLa cells at 37 °C under 15 min incubation, *λ*_ex_ 488 nm, under various conditions: (a) compound 2-BPin, at 100 μM (in 1 : 99% DMSO : RPMI) imaged before PBS washing; (b) compound 2-BPin, 1% DMSO, at 37 °C, colocalization with ER dye; (c) 2@glucan at 37 °C; (obtained by mixing 100 μM of 2-BPin and 10 μg mL^−1^ in glucan, 0.5 : 99.5% DMSO: RPMI); (a_1_)–(c_1_) overlay images of the green channel, red channel and DIC; (a_2_)–(c_2_) *λ*_em_ 516–530 nm (green channel); (a_3_)–(c_3_) *λ*_em_ 615–650 nm (red channel); (a_4_)–(c_4_) is the DIC channel. Scale bar: 50 μm. ESI,† shows additional micrographs.

Laser scanning confocal fluorescence (LSCFM) and epi-fluorescence imaging techniques were first used to assess the morphology and fluorescence emission distribution of the probes 1–3 in cells treated with their BPin-protected precursors. These assays were followed by a 2P FLIM measurements to analyse the lifetime change of fluorescent sensors in living cells (*vide infra*). Fluorescence microscopy experiments showed considerable fluorescence of 1–3 in living HeLa and PC-3 cells (Fig. 5). To further assess this, cells were treated with either 1, 2 or 3, and images taken after incubation for 15–20 minutes, where images shown in Fig. 5 and 6 show treatment groups with 15–20 minutes incubation with dyes at 37 °C. Furthermore, as expected, the emission of 3-Bpin in PC3 cells after 488 nm excitation was observed to occur within two different channels recorded (green, 515–530 nm and red, 605–675 nm, as shown in Fig. S61 and S62, in ESI†), and consistent with the observations for compounds 1–2 (seen in Fig. 5, 6 and ESI,† Fig. S47–S60). The results suggest that in the presence of polysaccharides such as glucans, supramolecular interactions occur which lead to the emergence of only a slight, hypsochromically shifted absorption and emission profile and this was reported previously for cyanine dyes where Pascal *et al.* previously hypothesized that this type of emission stems from aprotic environments around the dye, and may be used to probe intracellular environments.^25^

Several different uptake conditions were investigated including: (a) different fluorescent dyes in cells, (b) the same fluorescent systems tested under different culture conditions, such as concentrations, incubation times and temperatures. Pinacol deprotection of 1-BPin, 2-BPin and 3-BPin is facile and proceeded to formation of corresponding compounds 1–3*in situ* in presence of diol groups in β-d-glucan at pH 8–9.5 giving the desired diol-boronate and covalently-bonded supramolecular biopolymer complexes, denoted 1–3@glucan. The mechanism of formation likely involves exchanges under thermodynamic control: boronic acid–diol recognition event coupled with a single coil/triple helix rearrangement of the glucan chains at the interface between DMSO/aqueous media. Previous studies have indicated that borate anions form covalently-bonded complexes with diol groups of the β-d-glucan at *ca.* pH 8–9.5.^12,13,32^

**Fig. 7:**
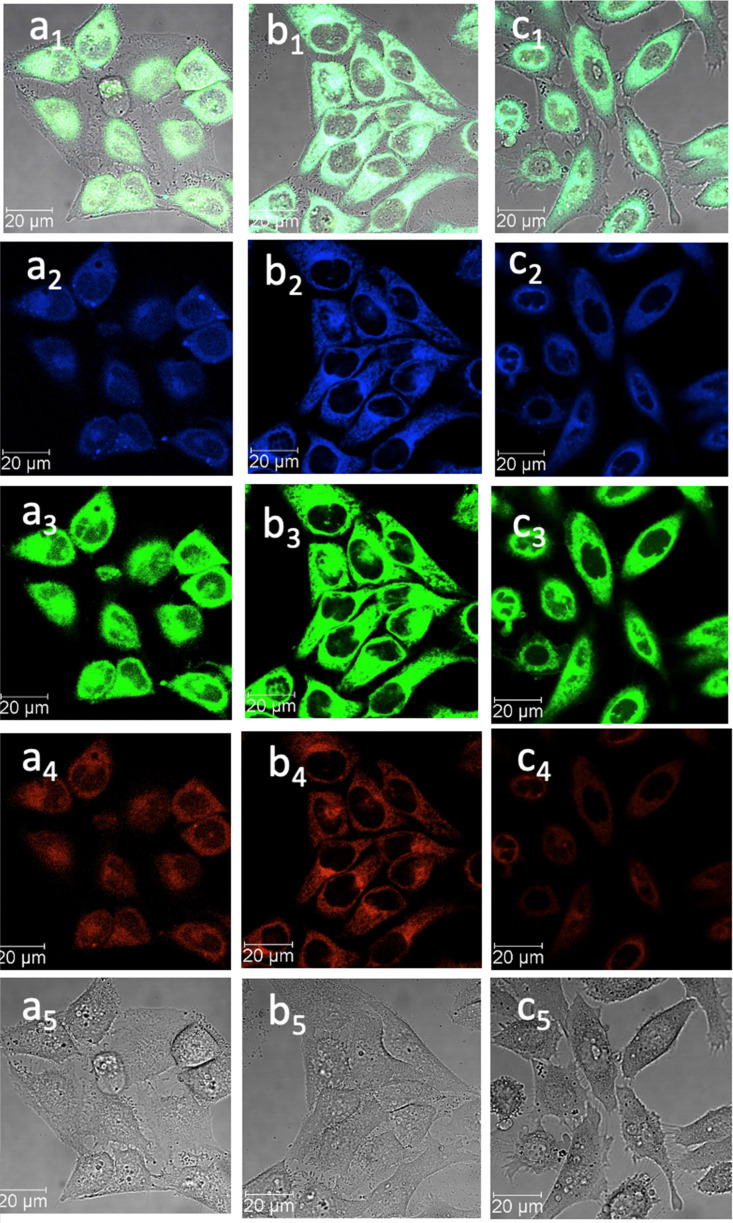
Confocal image of HeLa cells incubated with 1-BPin, 2-BPin and 2@glucan at 100 μM (2% DMSO) for 15 min at 4 °C *λ*_ex_ 405 nm; (a) 1-BPin; (b) 2-BPin, (c) 2@glucan, (obtained by mixing 100 μM of 2-BPin and 10 μg mL^−1^ glucan, 0.5 : 99.5% DMSO: RPMI); where (a_1_)–(c_1_) are the overlay images of the blue, green, red channels and DIC; (a_2_)–(c_2_) are the blue channels, *λ*_em_ 435–450 nm; (a_3_)–(c_3_) are the green channels, *λ*_em_ 516–530 nm; (a_4_)–(c_4_) are the red channels, *λ*_em_ 615–650 nm; (a_5_)–(c_5_) are the DIC channels. ESI,† shows additional micrographs.

Cancer cells were cultured according to our previously used and established methods^27,33^ and detailed imaging assays at either 37 °C or 4 °C were carried out. Fig. 5–7 show confocal images of HeLa cells incubated with either compound 1 or 2 under various laser excitations and temperature conditions and ESI,† shows additional images in living cancer cells and control experiments. Cells incubated with compound 1 showed morphologies that seem to suggest that their membranes were damaged because of the presence of this fluorophore at the higher concentrations (100 μM), yet imaging showed cells that were relatively unaffected in their morphology within 15–20 min of incubation with compounds 1–3 (as pinacol-protected or free boronates) in concentrations below 2 μM. For cells incubated with compound 2 (added as the BPin analogue), micrographs show bright, broad emissions both in green as well as red emission channels, and the morphology of cells appeared unchanged in that the cells were firmly attached, and membrane of cells appeared to remain undamaged.

This feature was consistent with the images obtained for compounds 3 and its *ortho*-substituted variant, compound III.^27^ Compounds 2 and 3 were introduced in cells as their pinacol-protected variants which prevented aggregation and/or dimerization prior to uptake, consistent with previously reported protocols.^12^ Most of the fluorescence emission appeared in the cytoplasm and there was no significant staining in the nucleus (Fig. 5, 6 and ESI†). Similar imaging assays were carried out in the presence of excess of beta-d-glucan, which we considered to act here as an adjuvant. Consistently with previous studies, we found that normalised cell viability tests of β-d-glucan is entirely non-toxic when cells treated at high concentration up to 2 mg mL^−1^ in PC-3, in 48 h assays performed either by MTT, so we anticipated that its presence is not damaging to cells. Confocal images of 1@glucan and 2@glucan showed similar results to those recorded at the imaging of the pinacol-protected compounds 1-BPin and 2-BPin respectively, in terms of the cytoplasmic distribution yet as expected significantly lower level of aggregation on cellular membranes (Fig. 5c and d).

When β-d-glucan anchored hybrids (denoted 1–3@glucan, respectively) were incubated at 4 °C there were virtually no changes in the fluorescence distribution and morphology of cells (as shown in Fig. 5–7 and in ESI†), which seems to suggest that for these compounds, lowering the incubation temperature had only a limited impact on the cellular uptake, pointing towards passive diffusion uptake mechanisms. Laser confocal microscopy of live HeLa cells incubated with compound 1-BPin (100 μM for 15 min, 1% DMSO, at 37 °C) recorded after PBS washing and laser irradiation also seems to suggest that exposure of cells to blue laser light leads to the emergence of membrane damage after cellular cytoplasmic uptake, however blebbing occurred only after laser irradiation over 5 min at 405 nm.

Laser confocal microscopy of live HeLa cells incubated with compound 1 (100 μM for 15 min, 1% DMSO, at 37 °C, and PBS washing) and colocalised with nuclear staining dye (Hoechst) are given in ESI.† Some (small amount) of nuclear uptake was noted in all these images, and for compound 2-Bpin, some endoplasmic reticulum co-localisation is plausible (Fig. 6b_1_–b_4_, and ESI†). Results showed the solubility of both compounds improves in presence of β-d-glucan, and there was no precipitation that necessitated PBS washing (*e.g.* for 1-BPin) before imaging occurred. Imaging of living cells shown in micrographs shown in Fig. 7 appears to indicate healthier cells for the duration of experiment (up to 1 h), and general much improved cellular morphology by comparison with that observed in micrographs 5-a. In the case depicted in the Fig. 6 set of micrographs, the cellular morphology itself seemed unchanged with respect to controls, and, for the case of compound 2-BPin incubated in the presence of excess β-d-glucan there was a slight decrease in the fluorescence intensity in all channels investigated after incubation at 4 °C, which indicated that the cellular uptake may well be driven by a combination of active and passive uptake rather than just by a simple diffusion. An analogous situation was determined following the imaging of compound 3-BPin in living cells, where a consistent behaviour was observed the for previously studied compounds III^27^ (ESI) and IV.^33^

Here, fluorescence lifetime imaging microscopy was utilized as an analytical tool aiming to probe the abilities of the NI probes 1–2 to respond to their environment. For compounds 1 and 2, either as free boronic acid compounds, as their BPin-protected precursors or glucan-formulated hybrids, the uptake in HeLa cells was visualised by combining laser scanning confocal microscopy and FLIM^13,34^ in the same field of view. We applied the well-known advantages of using lifetime imaging as ‘hyphenated’ (*e.g.* in a combined mode) due to expected improvements in single-photon specificity and sensitivity,^35^ sub-cellular resolution, reduced cytotoxicity by using multiphoton excitation are obtainable in the same field of view with single- as well as multi-photon excitation modes for the life-cells imaging. We particularly used 2P FLIM with either *λ*_ex_ = 810 or *λ*_ex_ = 910 nm excitation, due to the unique advantage presented especially since it allows sub-cellular diffraction limited mapping of the environment and interaction of probes in living cells.^36^ The additional advantage offered by this cellular imaging modality is that of environment sensing achievable when monitoring the cellular uptake of fluorophores in living cells, as opposed to simply using fluorescence emission intensity alone for the same probe. This bypasses limitations due photobleaching, spectral overlap, bleed through of different channels and co-localisation artifacts which do not necessarily infer molecular interactions. Excited state lifetime measurements evaluate an absolute length scale (on the nanometre range),^37^ and, as such, imaging assays using FLIM techniques are less vulnerable to factors such scattered light, photobleaching, non-uniform illumination of the probe, light path length, or laser intensity variations as these can normally be ‘gated’ out or easily corrected for. Therefore, 2P FLIM has been widely used in imaging cellular protein interactions^38–40^ and conformational changes,^41^ viscosity,^42,43^ temperature,^44^ pH,^45^ ions^46^ and oxygen concentrations.^47,48^ FLIM generated images based on the differences in the exponential decay rate of the fluorescence from a fluorescent sample on a pixel-by-pixel basis, whereby very high resolution at the diffraction limited level can be obtained. The lifetime decay of the fluorophore (rather than its emission intensity) was used hereby to generate the image or FLIM map (lifetime distribution across a field of view) for compounds 1–2 in cells. The average fluorescence lifetime of each of these the fluorophore was calculated at each pixel of a microscope image and fitted either as a single, double, or triple exponential (ESI†).

The nanosecond scale, excited-state, lifetime found for the NI cores of compounds 1–2 investigated hereby was independent of the probe concentration or light path length but may be dependent on the excited-state reactions. Therefore, 2P FLIM was applied as a reliable method for determining the lifetime of a fluorescent probe in each environment, and previously it has been applied to report on cellular viscosity as well as the degree of rigidity in the structural design of fluorescent probes. It has been shown that the magnitude of the emission lifetime may changes when a fluorescent probe binds to a target that could affect its environment.^45^

**Fig. 8:**
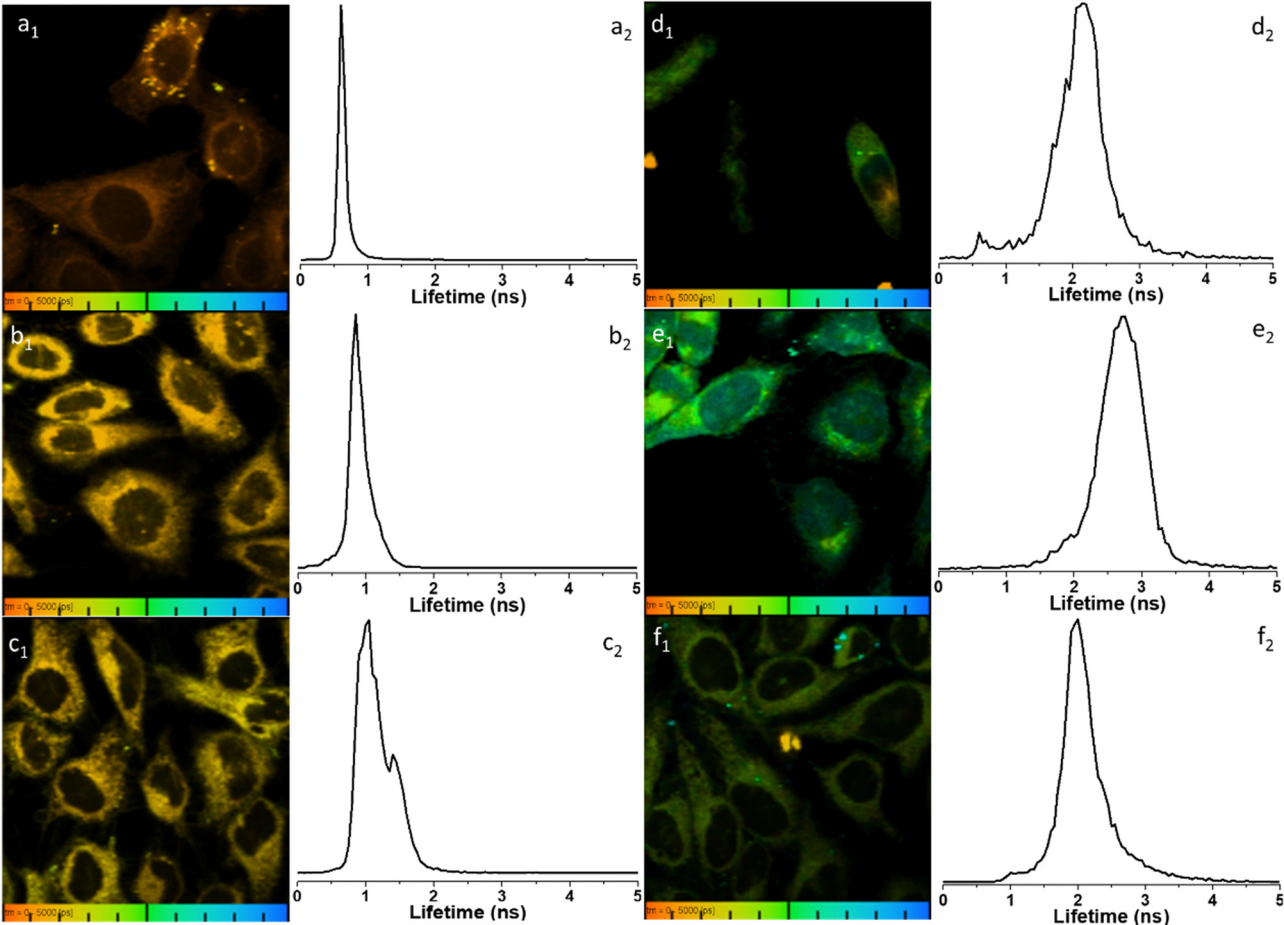
Representative micrographs for the two-photon fluorescence imaging including lifetime microscopy mapping of 1-BPin, 2-BPin (100 μM in 1 : 99% DMSO : RPMI) and corresponding hybrids 1@glucan and 2@glucan (100 μM in 1 or 2, and 10 μg mL^−1^ β-d-glucan, 0.5 : 99.5% DMSO : RPMI) in living HeLa cells, recorded immediately after 15 min incubation. Field of view (100 μm) is presented with corresponding fluorescence lifetime distribution curves 2P FLIM imaging of living cells treated with 100 μM of compounds in 1% DMSO, cells were incubated at 37 °C or 4 °C, scaled to 5 ns for comparison, where: (a) 2-BPin, 37 °C (b) 2@glucan, 37 °C (c) 2-BPin, 4 °C (d) 1-BPin, post PBS-washing, 37 °C (e) 1@glucan, 37 °C; (f) 1-BPin, 4 °C. (a_1_)–(f_1_) represent the 2P fluorescence lifetime map of compounds in HeLa cells, *λ*_ex_ = 910 nm and (a_2_)–(f_2_) curves represent *t*_m_, an average of *t*_1_ and *t*_2_ lifetime distribution of compounds in cells, *λ*_ex_ = 910 nm. Scale bar: 0–5 ns. Additional micrographs and as-fitted data are given in ESI.†

**Fig. 9:**
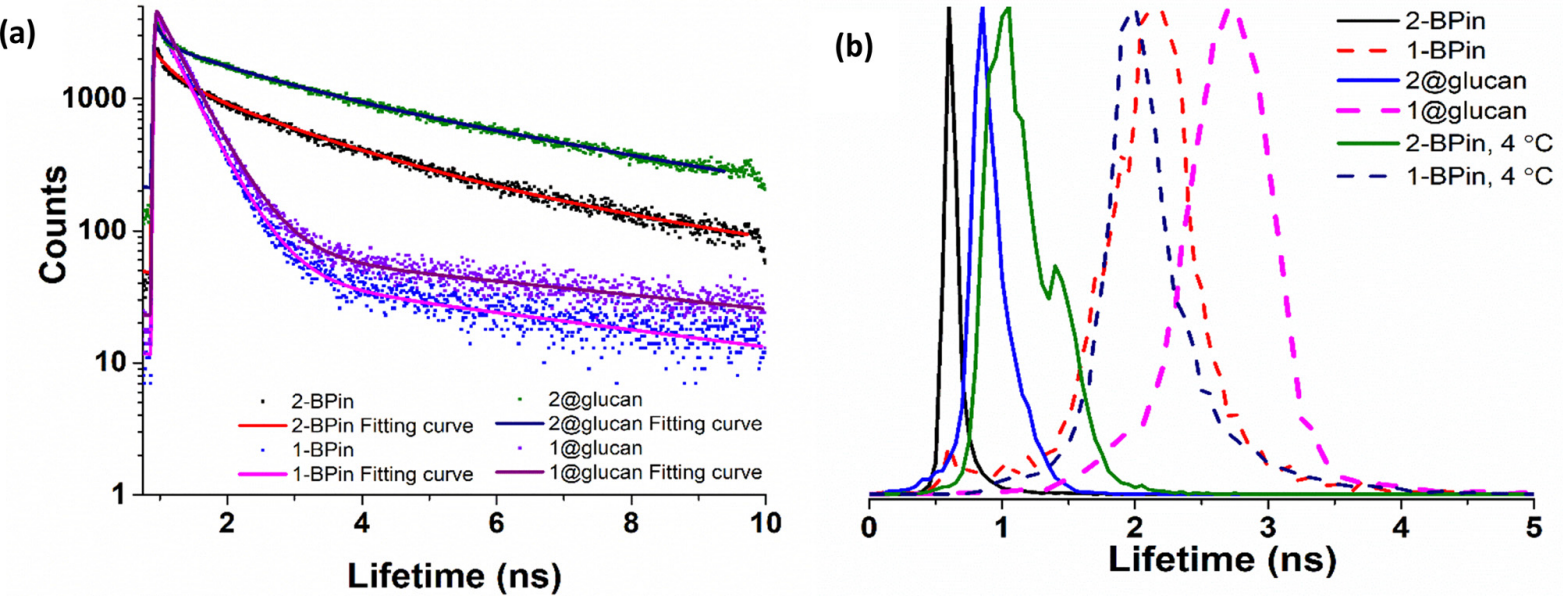
TCSPC and 2P FLIM comparisons: (a) overall TCSPC decay curves for compounds in DMSO, two-photon *λ*_ex_ 910 nm: (a) 2P TCSPC for compounds 1-BPin 1@β-d-glucan, **2-BPin**, 2@β-d-glucan where compounds 1-BPin and **2-BPin** were each incubated in presence of excess beta-d-glucan forming 1@β-d-glucan and 2@β-d-glucan, respectively *in situ*. (b) Overall lifetime distribution of compounds in cells, multiphoton *λ*_ex_ = 910 nm. Fitted data are given in ESI.†

To further elucidate the cellular uptake and biodistribution within cells, compounds 1-BPin and 2-BPin and the corresponding β-d-glucan hybrids of compounds 1–2 were investigated by two photon fluorescence lifetime imaging correlated with single photon confocal imaging, and results are depicted in Fig. 8, 9 and Table 1. Living cells treated with 100 μM of compounds in 1% DMSO incubated at 37 °C or 4 °C showed mainly cytoplasmic distribution. Interestingly, as seen in Fig. 7, micrograph e1, there appears to be some (minor) nuclear localisation of the probe. Imaging seems to indicate longer lifetime in the nucleus with respect to the cytoplasm, by *ca.* 1 ns. This is surprising considering that these images were recorded in samples of 1 incubated at 4 °C, and warrant further consideration, in future work. Some limited nuclear uptake was also seen for same compound, 2, at room temperature (Fig. 8 micrographs b1–b2, with lifetime of *ca.* 1 ns throughout the cells) lifetime distributions correlate well with solution measurements. The ‘rainbow’ coloured representation (Fig. 8, and ESI,† Fig. S66–S68) provides a direct correlation between the lifetime maps and the lifetime histograms (0–5 ns). Solution lifetime data (measured in 10 mM concentration solutions DMSO) by TCSPC (Table 1, and Fig. 9a) was then compared with data emerging from FLIM images (Fig. 9b and Table 1). Image 9-a shows an overlay of the measured TCSPC decay curves for compounds in DMSO (100 μM of compound 1-BPin and 2-BPin, respectively) measured in the absence, or presence, of 1 mg mL^−1^ beta-d-glucan, *λ*_ex_ = 910 nm. Behavior is comparable to the overall observed lifetime distribution of compounds in cells (*λ*_ex_ = 910 nm), as shown in figures (a_1_)–(f_1_) (which illustrate the fluorescence lifetime map of compounds in HeLa cells, *λ*_ex_ = 910 nm) and (a_2_)–(f_2_) (the lifetime distribution of compounds in these cells, for the corresponding field-of-view, *λ*_ex_ = 910 nm). These micrographs show that 1-BPin, 2-BPin, 1@glucan and 2@glucan species all penetrate the cell membrane, although the lower temperature reduces the extent of cellular internalisation, significant uptake is still noted at 4 °C. A closer inspection of FLIM micrographs and corresponding fitted lifetimes indicated that compound 2 showed an average lifetime of 1.96 ns in DMSO and this decreased to *ca.* 0.5 ns in HeLa cells at 37 °C. The average cellular lifetime increased to *ca.* 1.65 ns when the temperature decreased to 4 °C. Fluorescence lifetime changed to *ca.* 0.89 ns when compound 2 was anchored to β-d-glucan in DMSO, and, inside cells, 2@glucan again shows a shorter average lifetime, *i.e.* 1.23 ns in HeLa cells at 37 °C. Interesting, compound 1-BPin has a shorter average lifetime in DMSO, *i.e.* 0.48 ns and this compares well with that of the corresponding hybrid 1@glucan, which was 0.50 ns. These lifetimes increase significantly to 2.86 ns in HeLa cells at 37 °C and upon temperature change to 4 °C, a minor reduction in average lifetime to *ca.* 2.34 ns was observed. These variations may appear rather small considering the change in environment however they seem to suggest that presence of large biomolecular species influences the lifetime of the fluorophore, whilst the order of magnitude remains comparable. Although both compounds 1 and 2 have in common the same naphthalimide core, the corresponding peripheral functional groups and therefore their overall aggregation capabilities in solution differ considerably: these structural differences are reflected in the lifetime data in solution (in the presence and absence of a biopolymer) and in the corresponding lifetime features of the probe when present in cellular environments.

**Table 1:** Fitted parameters for the two-photon excitation time-correlated single photon counting decay curves at 910 nm excitation (1-BPin and 2-BPin solutions were 10 mM in DMSO and corresponding 1–2@β-d-glucan hybrid was in 1 : 1 DMSO : PB Buffer. Corresponding 2P FLIM data associated to micrographs of living HeLa cells depicted in Fig. 9, and ESI. The full width at half maximum (FWHM), calculated from the lifetime distribution curve within the focal area was used to assess the 2P lifetime distribution error

2P TCSPC (*λ*_ex_ 910 nm)							2P FLIM (*λ*_ex_ 910 nm, 15 min in HeLa, peak ± error, ns)	
	*χ* ^2^	*τ*_1_ (ns)	*a*_1_ (%)	*τ*_2_ (ns)	*a*_2_ (%)	*τ*_m_ (ns)		
1-BPin	1.24	0.41	98.9	5.94	1.1	0.48	1-BPin, 37 °C	2.86 ± 0.37 ns
							1-BPin, 4 °C	2.34 ± 0.23 ns
1@glucan	1.38	0.43	98.5	5.62	1.5	0.50	1@glucan, 37 °C	3.49 ± 0.48 ns
2-BPin	1.35	0.60	49.2	3.27	50.8	1.96	2-BPin, 37 °C	0.50 ± 0.07 ns
							2-BPin, 4 °C	1.65 ± 0.24 ns
2@glucan	1.46	0.89	41.2	4.47	58.8	2.99	2@glucan, 37 °C	1.23 ± 0.18 ns

These changes also imply that for these compounds the impact on conformational changes induced by the presence of glucan (in solution, at the room temperature) is comparable, in terms of magnitude of fluorophore's lifetime, to the impact on the cellular uptake that lowering the temperature as indicated by the magnitude of the corresponding fluorescence lifetime. These observations map well onto our earlier studies on the behaviour of common boronic-acid functionalised dyes and β-d-glucan, where we investigated the lifetime of fluorescein and coumarin-related compounds under similar assays conditions.^12,13^

As the cellular penetration abilities and cytoplasmic uptake of the (pinacol-protected) compounds 1–3 was significant (albeit without significant nuclear uptake), we next investigated their cytotoxicity properties in PC-3 cells using standard 48 h MTT assays cytotoxicity assay. Experiments at each of the concentrations used were repeated six times, error bars stand for standard error calculated from six repeated measurements. Notably, compound 1-BPin displayed severe cytotoxicity against this malignant cancer cell line with an IC_50_ value of IC_50_ = 2.06536 × 10^−7^ ± 9.7116 × 10^−8^ M, showing that this compound is significantly more toxic than both compounds 2-BPin (IC_50_ = 1.83593 × 10^−5^ ± 3.6745 × 10^−6^ M) and 3-BPin (IC_50_ = 1.06607 × 10^−5^ ± 0.22857 × 10^−6^ M) in PC-3 cells. The 48 h toxicities of the (**BPin** protected) NI-boronic acids compounds **1–3** were also comparable to that seen in the same assay, carried out hereby, for cis-platin in PC-3 (IC_50_ = 3.06415 × 10^−5^ ± 3.258816 × 10^−6^ M).

## 3. Conclusions

We investigated and report hereby three novel naphthalimide-based fluorophores and derivatised these further with beta-d-glucan, as model polysaccharides structurally related to the glycan-based receptor overexpressed on surface of cancer cells, and glycated proteins. These new NI-boronic acid conjugates were shown to have potential in cellular imaging applications. A tailor-made design aimed to maximise the ability of the boronic acid unit to interact with polysaccharides, whether in solution, or those present on the surface of cancer cells, whilst allowing the NI unit to report on the cellular uptake in an unhindered way. We also carried out new investigations into the potential of boronic acids derived from the naphthalimide core and the formation of their hybrids with a non-toxic, naturally occurring biopolymer, β-d-glucan. We then explored the potential of such bioconjugates to interact with cancer cells to gain an insight into their potential as cellular translocation entities and biocompatible imaging agents using multiphoton fluorescence imaging techniques as an analytical tool. Images of different cell lines (recorded under varying environmental conditions such as temperature changes and using laser scanning confocal microscopy together with FLIM, with single photon and two photon excitations) showed notable differences in the way these structurally related probes report on their environment. Using steady state confocal microscopy alone, all compounds investigated show a good uptake across the cell membrane however for compound 1 cellular morphology seem to be affected additionally to the fact that this compound also showed significant aggregation on the cellular membrane, which remains in place even after extensive washing with PBS. Such complex behaviour at uptake appears to be favourably mediated by anchoring these compounds onto β-d-glucan. Thus, introducing glucan as a cellular uptake mediator improves the uptake of compound 1 and significantly enhance the solubility of all compounds 1–3, although images recorded in its presence also showed some quenching of the 1–3 fluorescence emissions throughout the cytoplasm. Multiphoton FLIM investigations at varying temperatures showed that by incubating cells at lower temperatures (4 °C) does not fully inhibit the uptake of these fluorescent compounds but minimises the cellular damage caused by these compounds. The fluorescence lifetime observed under variable temperature conditions changed subtly indicating different environments are being sensed by the probes. Solution phase TCSPC measurements of the fluorescence lifetime showed multi-exponential fitting, consistent with aggregation in solution. Within cells, and in the complex environment of the polysaccharides such as β-d-glucan, it is possible to envisage that binding and changes of conformation in the compounds, including aggregation, present multi-exponential decay characteristics in terms of emission lifetime. These are of the same order of magnitude with the values observed in solution, yet subtle changes occur, allowing us to propose that these probe report successfully on changes in their environments. These investigations permitted us to observe how the subtle structural differences between compounds can lead to behaviour differences in both solution and cellular environments, including changes in cellular uptake and viability.

## Data availability

Data for this research are given in the ESI† and available from the authors upon request.

## Author contributions

S. I. P.: conceptualisation; methodology; validation; investigation; project administration; funding acquisition; supervision; writing – reviewing & editing; visualization. T. D. J.: conceptualisation; methodology; supervision; writing – reviewing, funding acquisition. S.-Y. X.: conceptualisation; methodology; investigation; writing – reviewing. S. W. B.: validation; investigation; supervision; writing – reviewing; visualization. C. P.: validation; investigation; supervision; writing – reviewing; S. E. F.: methodology; investigation; formal analysis; supervision; M. J. G.: methodology; investigation; formal analysis; visualisation, writing – reviewing; H. G.: methodology; investigation; formal analysis; visualisation, writing – reviewing; N. K.: methodology; investigation; M. Li: methodology; investigation; H.-C. W.: methodology; investigation; G. K.-K: investigation; resources; writing – editing.

## Conflicts of interest

There are no conflicts to declare.

## Supplementary Material

CB-004-D3CB00112A-s001

CB-004-D3CB00112A-s002

CB-004-D3CB00112A-s003

CB-004-D3CB00112A-s004
