# Supplementary material for: Fluorescent naphthalimide boronates as theranostics: structural investigations, confocal fluorescence and multiphoton fluorescence lifetime imaging microscopy in living cells
Source: RSC Chem Biol. 2023 Sep 18;4(12):1082–95. doi: 10.1039/d3cb00112a (PMC10685793; doi:10.1039/d3cb00112a)
Supplement: CB-004-D3CB00112A-s003 [file CB-004-D3CB00112A-s003.pdf]

Electronic Supplementary Material (ESI) for RSC Chemical Biology.
This journal is © The Royal Society of Chemistry 2023

Supplementary Materials

Fluorescent Naphthalimide Boronic Acid-Based Theranostics: Structural Investigations and Multiphoton Fluorescence Lifetime Imaging Microscopy

Megan J. Green,[a, #] Haobo Ge, [a, #] Stephen E. Flower, [a] Charareh Pourzand, [b,c] Stanley W. Botchway,[d] Hui Chen Wang, [a] Navaratnarajah Kuganathan,[e] Gabriele Kociok-Köhn,[a,f] Meng Li,[a,g] Suying Xu, [a,h]* Tony D. James [a] * and Sofia I. Pascu [a,c] *

[a] Megan J. Green, Dr. Haobo Ge, Dr. Stephen E. Flower, Dr. Hui Chen Wang, Dr. Gabriele Kociok-Köhn, Dr. Meng Li, Dr. Suying Xu, Prof. Tony D. James, Prof. Sofia I. Pascu
Department of Chemistry, University of Bath, Claverton Down Rd., BA2 7AY, Bath, United Kingdom
E-mail: s.pascu@bath.ac.uk, t.d.james@bath.ac.uk

[b] Dr. Charareh Pourzand
Pharmacy and Pharmacology, Department of Life Sciences, University of Bath, BA2 7AY, Bath, UK

[c] Prof. Sofia I. Pascu and Dr. Charareh Pourzand
Centre of Therapeutic Innovations, University of Bath, Claverton Down Rd., BA2 7AY, Bath, UK

[d] Prof. Stanley W. Botchway,
Research Complex at Harwell, Central Laser Facility, Science and Technology Facilities Council, Rutherford Appleton Laboratory, Harwell Science and Innovation Campus, Harwell, Oxfordshire OX11 0QX, UK

[e] Dr. Navaratnarajah Kuganathan,
Department of Materials, Imperial College London, London, SW7 2AZ

[f] Dr. Gabriele Kociok-Köhn,
Materials and Chemical Characterisation Facility (MC2), University of Bath, Calverton Down, Bath, BA2 7AY

[g] Dr. Meng Li,
State Key Laboratory of Chemical Resource Engineering, Beijing University of Chemical Technology, Beijing 100029, PR China

[h] Dr. Suying Xu,
Hebei Key Lab of Power Plant Flue Gas Multi-Pollutants Control, Department of Environmental Science and Engineering, North China Electric Power University, Baoding 071003, P. R. China
E-mail: syxu@mail.buct.edu.cn

Content:

1. General Experimental Methods..……………………………………….……………S3
2. Synthetic protocols……………………………………………………………...S9
3. Spectroscopic data: isolated intermediates, Compounds 1-3 and III……….……S15
4. Cellular Imaging and MTT assays……………………………….….…….....S46
5. Structural Investigations of Compound 3-BPin by Gas Phase DFT Calculations…..S63
6. Structural Investigations of 3-BPin by Single Crystal X-ray Diffraction Analysis…S68

1. General Experimental Methods.

Solvents and reagents were reagent grade unless stated otherwise and were purchased from Acros Organics, Avocado Research Chemicals Ltd, Fisher Scientific UK, Frontier Scientific Europe Ltd, Alfa Aesar, TCI, Lancaster Synthesis Ltd and Sigma-Aldrich Company Ltd and were used without further purification, unless stated otherwise. Anhydrous solvents were collected from the SPS system. Purification of products by flash chromatography was carried out using CH_2Cl_2:CH_3OH, 50:1, v/v.

Nuclear magnetic resonance spectra were performed in DMSO-d_6, MeOD-d_4 or $CDCl_3$. ^1H, ^{11}B and ^{13}C spectra were measured using a Bruker AVANCE 250, Bruker AVANCE 300, Bruker AVANCE 400 or Bruker AVANCE 500 spectrometer. ^1H spectra were recorded at 300.22 MHz or 500.13 MHz, ^{11}B spectra at 96.32 MHz and $^1H\{^{13}C\}$ at 69.5 or 75.5 MHz. Some titration experiments were recorded Chemical shifts (δ) are expressed in parts per million and are reported relative to the residual solvent peak or to tetramethylsilane as an internal standard in ^1H and $^1H^{18}$ spectra, or boron trifluoride diethyl etherate as an external standard in ^{11}B spectra. The multiplicities and general assignments of the spectroscopic data are denoted as: singlet (s), doublet (d), triplet, (t), quartet (q), doublet of doublets (dd), doublet of triplets (dt), triplet of triplets (tt), unresolved multiplet (m), apparent (app), broad (br) and aryl (Ar). Coupling constants (J) are expressed in Hertz.

High resolution mass spectra were recorded by the EPSRC National Mass Spectrometry Service Centre, Swansea. Low resolution EI and CI measurements were performed on a Micromass Quattro II triple quadrupole instrument, with ammonia as the CI reagent gas. Electron impact (EI) and chemical ionisation (CI) analyses were performed in positive ionisation mode. Electrospray ionisation measurements were performed in both positive and negative ionisation modes (ESI + and ESI - respectively). High resolution ESI + and ESI - measurements were conducted on the micrOTOFQ electrospray time-of flight (ESI-TOF) mass spectrometer (Bruker Daltonik GmbH) at the University of Bath. The micrOTOFQ spectrometer was coupled to an Agilent Technologies 1200 LC system. 10 μL of sample was directly injected into the mass spectrometer. The nebulising gas used was nitrogen, which was applied at a pressure of 1 bar. Nitrogen was also used as a drying gas, supplied at a flow rate of 8 L/min and a temperature of 110 °C.

HPLC was performed using a Gilson HPLC system using a Dionex C18 Acclaim column (5 μM, 4.6 x 150 mm). A 15 minute gradient method was applied using H_2O/ MeCN containing 0.1 % TFA as mobile phases with the following conditions: flow rate 1 mL/min, 0 min 20 % MeCN; 1 min 20 % MeCN; 4 min 95 % MeCN; 11.5 min 95 % MeCN; 13.5 min 15 % MeCN , 15 min 15 % MeCN. Absorbance was measured at 254 nm and 350 nm.

Steady-state fluorescence measurements were performed on a Perkin-Elmer Luminescence Spectrophotometer LS 50B, utilising Starna Silica (quartz) cuvettes with 10 mm path lengths, four faces polished. Data was collected via the Perkin-Elmer FL Winlab software package. All pH measurements taken during fluorescence experiments were recorded on a Hanna Instruments HI 9321 Microprocessor pH meter which was routinely calibrated using Fisher Chemicals standard buffer solutions (pH 4.0 - phthalate, 7.0 – phosphate, and 10.0 - borate). All solvents used in fluorescence measurements were HPLC or Fluorescence grade and Milli-Q water was used throughout. Relative quantum yields were determined by comparison with fluorescein in 0.1 M NaOH using the following formula: $\Phi_S = \Phi_R(D_S/D_R)(A_R/A_S)(I_R/I_S)(\eta_S/\eta_R)^2$, where Φ is the relative quantum yield, D is the integrated area of the fluorescence emission peak, A is the absorption of the solutions at the excitation wavelength, I is the flux at the excitation wavelength used and η is the solution refractive index. R and S subscripts refer to the reference and sample respectively. For stability

studies, stock solutions of complexes were prepared as 7.5 or 2.5 mM solutions in DMSO and diluted with RPMI serum, giving solutions of final concentration 7.5 μM or 2.5 μM concentrations. The solutions were kept at 37 °C and UV/Vis and fluorescence spectra were recorded at time points 1 min, 1 h, 2 h, 4 h, and 24 h.

Two photon (690-1000 nm) wavelength laser light was obtained from the mode locked titanium- sapphire laser Mira (Coherent Laser Co., Ltd.) produced by 180 femtosecond pulse frequency of 75 MHz. This laser-pumped solid-state continuous wave 532 nm laser (Verdi V18, Coherent Laser Co., Ltd.). This can also be used for the fundamental output of the oscillator 915 ± 2 nm. The laser beam was focused to a diffraction-limited spot by the water immersion UV calibration target (Nikon VC × 60, NA1.2) and the specimen on a microscope stage of the modified Nikon TE2000-U, with UV illumination optical emission. The focused laser beam raster scanning used an X - Y galvanometer (GSI Lumonics Corporation). Fluorescence emission was collected without de-scanning, bypassing the scanning system and passed through a coloured glass (BG39) filter. In normal operation mode and line scan frame and pixel clock signal was generated with an external fast microchannel plate photomultiplier tube as detector (R3809 - U, Hamamatsu, Japan) synchronisation. These were linked to a Time Correlated Single Photon Counting (TCSPC) PC module SPC830 for the lifetime measurements with 910 nm excitation and emission in the range between 360 and 580 nm. Lifetime calculations were obtained using SPCImage analysis software (Becker and Hickl, Germany) or Edinburgh Instruments F900 TCSPC analysis software. Preliminary single-photon FLIM investigations were conducted using the Becker and HicklDCS120 system with a 40 ps 473 nm diode laser.

Compound@glucan hybrid preparation:
A stock solution of compound (10 mM, 1 mL) dissolved in DMSO was added to b-D-glucan (1 mg/mL, 1 mL) in DMSO, the pH was adjusted to 7.4 using PBS buffer and the mixture was stirred at room temperature overnight, in a vial protected from light. This was used directly for 2-photon TCSPC and cellular imaging experiments.

Cell culture:

Cell lines used in live cell imaging include cervical cancer cells (HeLa), and prostate cancer cells (PC-3). All cells lines were from American Type Cell Culture (ATCC). Cells were normally frozen at -196 °C in liquid nitrogen until required, then thawed quickly and incubated after the addition of fresh media at 37 °C under 5% carbon dioxide environment. Eagle's Minimum Essential Medium (EMEM), Park Memorial Institute (RPMI) medium and Dulbecco's Modified Eagle's Medium (DMEM) were used as culture media. All media contained activated foetal calf serum (FCS) (10% for HeLa, PC-3), 0.5% penicillin/streptomycin (10,000 IU mL^{-1}/10,000 mg mL^{-1}) and 2.5% L-glutamine.

Cell subculture was performed once or twice per week depending on the confluence of the cells in the flask, the supernatant was aspirated. All attached cells were washed twice using phosphate buffered saline (PBS, 2 x 10 mL), (all solvents, buffer solutions and media were warmed to 37 °C in the water bath prior to addition) 5 mL of 0.25% trypsin (in PBS) was subsequently loaded and incubated at 37 °C for 5 minutes. After trypsinisation, 6 mL serum medium was added to neutralise the excess trypsin and solution was centrifuged at 1000 rpm for 5 minutes. Afterwards, the supernatant was aspirated and resuspended with 5 mL serum medium. Cells were counted in a haemocytometer and seeded appropriately for different applications.

Cellular viability assays:

After cell splitting, cells were seeded on a sterile 96 well plate (7×10^3 cells per well) and incubated for 48 hours to adhere. Compounds 1, 2 and 3 were subsequently loaded at different concentrations into wells and cultured for another 48 hours. The concentration used were 250 μM (1% DMSO, 99% culture media (10% FCS)), 100 μM, 50 μM, 10 μM, 1 μM, 0.5 μM, 0.1 μM and 1 nM. Subsequently, cells were washed with PBS and 3-(4, 5-dimethylthiazol-2-yl)-2, 5-diphenyltetrazolium bromide (MTT) was added (100 μL, 0.5 mg/ mL, 1:9 PBS: serum-free medium (SFM)) followed by a two-hour incubation. After aspiration, 100 μL of DMSO was added and 96 well plates were read by an ELISA plate reader, Molecular Devices Versa Max (BN02877). The absorption wavelength was at 570 nm and 630 nm wavelength was used as a reference.

Fluorescence microscopy

Method 1. For confocal- or epi-fluorescence microscopy cells were plated in glass-bottomed dishes as 1.5 x 10^5 cells per dish and incubated for 24 h for HeLa. Cells were washed 5 times with PBS, before adding SFM (2 mL). Subsequently, a small volume of SFM was removed (e.g. 10 μL) and compound in DMSO was added in equal volume to what was removed, to obtain a final volume of 2 mL and the desired concentration (500 mM, 5 % DMSO, 50 mM, 0.5 % DMSO or 2 mM, 0.5 % DMSO). After minimum 20 min cells were washed 3 times with PBS and incubated in SFM prior to imaging

Fluorescence microscopy images were captured on a Nikon Eclipse TE2000-E epifluorescence or a Zeiss LSM510META microscope at University of Bath, whereas at the Rutherford Appleton Laboratory a modified Nikon TE2000-U was utilised. HeLa cells were cultured as above and plated in a glass bottomed petri dish (MaTek, 35 mm diameter and 1.5 mm thickness) at 1.5×10^5 -2.5×10^5 cells per dish depending on the cell line and culture time. Briefly, HeLa cells required 12 hours to attach onto the dish, whereas CHO and PC-3 needed 48 hours to adhere. Prior to the microscopy observation, cultured cells were washed with Hank's solution (HBSS) (5×1 mL) and refilled with serum-free medium (990 μL). Prior to the probe labelling, a small volume of compound (10 mM or 1 mg/mL stock solution, 10 μL) in DMSO was loaded to make the final volume 1 mL at the appropriate concentration. The final concentration on the plate was usually at 100 μM with 1% - 2% DMSO depending on the compound solubility. After 15 min to 1-hour incubation at 37 °C or 4 °C, cells were washed with HBSS three times and refilled with fresh SFM (1 mL). Confocal images were captured immediately afterwards.

Method 2: Confocal fluorescence microscopy images were captured on modified Nikon TE2000-U at the OCTOPUS Facility, Research Complex at Harwell. HeLa or PC3 cells were cultured as above and plated in a glass bottomed petri dish (MaTek, 35 mm diameter and 1.5 mm thickness) at 1.5×10^5 -2.5×10^5 cells per dish depending on the cell line and culture time. Briefly, HeLa cells required 12 hours to attach onto the dish, whereas PC3 needed 48 hours to adhere. Prior to the microscopy observation, cultured cells were washed with Hank's solution (HBSS) five times and refilled with 990 μL of serum-free medium. Prior to the probe labelling, a small volume of compound (10 mM or 1 mg/mL stock solution, 10 μL) in DMSO was loaded to make the final volume 1 mL at the appropriate concentration. The final concentration on the plate was usually at 100 μM with 1% - 2% DMSO depending on the compound solubility. After 15 min to 1-hour incubation at 37 °C or 4 °C, cells were washed with HBSS three times and refilled with fresh SFM (1 mL). Confocal images were captured immediately afterwards.

Colocalization tests:
Several commercial (Invitrogen) cellular organelles tracking dyes were used to localise distribution of samples via confocal microscopy; all dyes in the following studies were purchased from Invitrogen. Cells were cultured in Eagle's Minimum Essential Medium (EMEM) or Roswell Park Memorial Institute (RPMI)-1640 medium using standard protocols described above. Prior to addition of any commercial co-localisation dye, cells were washed five times with HBSS. Protocols were adapted from Invitrogen.

i) Staining with Hoechst solution. The Hoechst co-localisation dye solution (10 mg/mL) was prepared by dissolving 10 mg Hoechst 33342 (as supplied by Invitrogen) in 1 mL of sterile MilliQ water, 10 µL of the stock solution was dissolved into 990 µL sterile water to make a 100 µg/mL stock solution. Cells were then washed five times with HBSS, and then refilled with 990 µL SFM. 10 µL of stock solution was then added to the 990 µL SFM. Cells were then incubated for 20 to 30 minutes. Subsequently, cells were washed three times with HBSS and refilled with 1 mL SFM to take control images. Then, cells were washed and the compounds of interest were loaded. After 15 min incubation with the compound, cells were washed three times with HBSS and refilled with fresh SFM (1 mL). Confocal images were recorded immediately afterwards.

ii) Staining with endoplasmic reticulum ER tracker. The commercially available ER tracker Red was used as received from Invitrogen, as a 1 mM aqueous solution. From this 10 µL was diluted with 90 µL DMSO to obtain 100 µM stock solutions. Then, cells were washed five times with HBSS and plates were refilled with 990 µL SFM. Subsequently, 10 µL of the diluted stock solution (prepared as above) was added to make 1 µM solutions on the cell plate. Cells were incubated for 15 to 30 minutes and subsequently cells were washed three times with HBSS. The plates were refilled with 1 mL SFM to take control images cells stained with co-localisation dyes. Finally, cells were washed and the compound of interest for co-localisation studies was loaded. After 15 min incubation with the compound, cells were washed three times with HBSS and refilled with fresh SFM (1 mL). Confocal images were recorded immediately afterwards.

iii) Staining with Nile Red. A stock solution of 10 µg/mL Nile Red dye from Invitrogen was prepared in DMSO. To 10 µL of stock solution, a 990 µL aliquoted was added, resulting in a final concentration of 100 ng/mL. After cells were incubated with this solution for 10 min a further 3 rounds of washings with HBSS were carried out, before the addition of 1 mL of serum free medium (SFM). Subsequently, a small volume of SFM was removed (e.g. 10 µL) and the desired compound dissolved in DMSO was added in a volume equal to that which was removed previously, to obtain a final volume of 1 mL and the desired concentration. After 20 min incubation with the compound, cells were washed 3 times with PBS and subsequently fresh serum free medium was added (1 mL). Images were recorded immediately afterwards.

Fluorescence lifetime Measurements:

Time-correlated single-photon counting (TCSPC): Lifetime measurements were recorded using a Becker and Hickl SPC830 TCSPC PCI module with an excitation wavelength of 910 nm and the emission measured at 360-580 nm (BG39, Comar Optics, UK). The measurement of compounds was carried out in DMSO/ water solvent with concentration of 10 mM or less. Emission spectral detection was carried out using an acton Research Component 275 spectrograph and an Andor iDus 740-BU CCD camera.

Two-photon Fluorescence lifetime imaging microscopy (FLIM): Cell uptake studies were performed using HeLa and PC3 cells. Cells were plated on a glass petri dishes and left cells to attach for 12-24 hours depending on the growth rate. Control FLIM were recorded before the addition of compound. Plates were then mounted on the microscope stage and kept at 37 ºC. Compounds were dissolved in DMSO and added to cells to achieve final concentration of 100 μM in EMEM/RPMI medium containing 1-2% of DMSO. Cells were incubated for 15 minutes prior to confocal imaging, 2P FLIM was carried out immediately after the confocal imaging with the same area as the confocal imaging. The fluorescence decay curves were fitted to a multiexponential function as:

$$I(t)=\sum_{i} a_i exp(\frac{-t}{\tau_i})$$

Where I(t) is the fluorescence decay, τ_i is the lifetime of component i and a_i its intensity contribution to the fluorescence decay. For 2P FLIM, the full width at half maximum (FWHM), calculated from the lifetime distribution curve within the focal area was used to assess the error. The percentage of components τ1 (major) and τ2 (minor) in cells was obtained from the respective amplitudes a1 and a2 calculated using SPCImage software, which models the data for each individual pixel to Equation below, where F is fluorescence, a0 is background and t is

$$F(t) = a_0 + a_1 e^{-t/\tau_1} + a_2 e^{-t/\tau_2}$$

time:

Saccharide titrations

The boronic acid moiety on compound **III** was protected by a pinacol group, giving **III-BPin**. The dynamic equilibrium process between boronic acid and diol can lead to the formation of more stable boronate ester if two different types of diols existed, as the binding constant between pinacol and boronic acid is far less than that with saccharide. All the tested monosaccharides induced a steady decrease of fluorescent intensity of compound **III**. The estimated binding constants indicated rather weak binding interactions, following the well-established selectivity orders for the binding of boronic acid derivatives to D-fructose > D-galactose > D-mannose > D-glucose.

Fluorescence titration of Compound III with β-D-glucan

A stock solution of 'receptor' or 'host' (10 mM) was made by dissolving fluorophore in DMSO. β-D-glucan (from barley, Aldrich) was completely dissolved in pH 8.21 aqueous buffer by heating (50 °C), which generated this 1 mg/mL solution (considered the 'guest'). Increasing volumes of the guest were transferred to a 1 mL receptor solution in a fluorescent spectroscopy cuvette and were left for 5 minutes to allow complete guest and receptor binding. Next, the same volume of the mixture was removed maintaining the total volume unchanged after each titration. Fluorescence emission spectra were recorded after each titration. The observed stability constants (K) with a coefficient of determination (r^2) were calculated by the fitting emission intensity at a single wavelength versus polysaccharide concentration using custom written non-linear curve fitting (equation 1) within the Origin 9.0 software. The binding constant (K) was determined by fitting a 1:1 binding model to I_F/I_{Fmax}.

$$y = (1 + A_1 * A_2 * x) / (1 + A_1 * x) \quad (1)$$

y: I_F/I_{Fmax}

A_1: K (binding constant of the receptor with the guest)

A_2: I_{Fmin}/I_{Fmax} x: the concentration of β-D-glucan added to a fluorophore

I_{Fmin} is the final (minimum) fluorescence intensity;
I_{Fmax} is the initial (maximum) fluorescence intensity;
I_F is the fluorescence intensity after addition of β-D-glucan (considered as 'guest').

X-ray Crystallography

Intensity data for Compound **3-BPin** were collected at 150(2) K on a Rigaku SuperNova Dual EosS2 single crystal diffractometer using monochromated Cu-Kα radiation (λ = 1.54184 Å). Unit cell determination, data collection data reduction and absorption correction were performed using the CrysAlisPro software [1]. The structures were solved with SHELXT [2] and refined by a full-matrix least-squares procedure based on F2 (SHELXL-2018/3) [2]. All non-hydrogen atoms were refined anisotropically. Hydrogen atoms were placed onto calculated positions and refined using a riding model. Where possible hetero atom hydrogen atoms have been located in the difference Fourier map and were refined freely or with bond length restraints. Additional programmes used for analysing data and their graphical manipulation included: SHELXle [3], ORTEP3 for windows [4] and Mercury. [5]

Computational details

We used density functional theory (DFT) calculations as implemented in the Gaussian 09 package [6] to examine the structures, estimate the Mulliken charges on each of the atoms in the relaxed structures and construct molecular orbitals to predict the band gap that associated with the degree of charge transfer. The exchange correlation energy was modelled using the schemed parameterised by Perdew, Burke and Ernzerhof Perdew (PBE) [7]. We used PBEPBE exchange correlation functional and 6-31G** basis sets for all atoms (B, C, N, O and H) as implemented in the code. Mulliken analysis [8] was used to estimate the charges on the atoms in the relaxed optimised structures. GaussView 6.1.1 [9] was used to construct the corresponding frontier molecular orbital diagrams.

2. **Synthetic protocols**

2.1. Synthesis of Compound 1.

Step 1. Synthesis of 6-bromo-2-butyl-1H-benzo[de]isoquinoline-1,3(2H)-dione (Intermediate 1a)

4-Bromo-1,8-naphthalic anhydride (0.30 g, 1.08 mmol) was suspended in ethanol and warmed slightly to get a clear solution. One equivalent of butylamine (1.08 mmol) was then added slowly to the solution. After refluxing for 6 hours the pale-yellow suspension was collected by vacuum filtration, and the resulting yellow solid was washed with ethanol and used without further characterisation in Step (b).

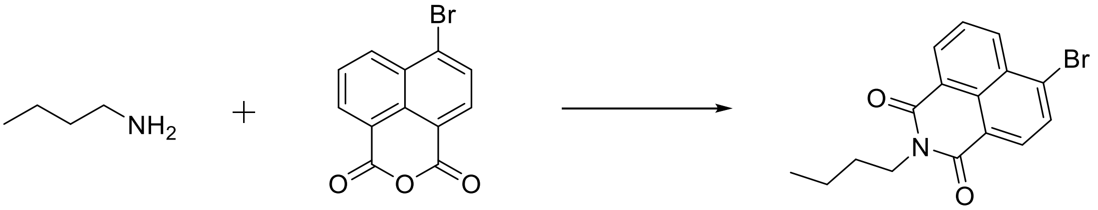

Scheme S1. The synthesis of Intermediate **1a**

Step 2. Synthesis of 2-butyl-6-((2-(methylamino)ethyl)amino)-1H-benzo[de]isoquinoline-1,3(2H)-dione (Intermediate 1b)

Intermediate **1a** (0.39 g, 1.17 mmol), N-Methylethylenediamine (0.09 g, 1.17 mmol) and triethylamine (0.12 g, 1.17 mmol) were suspended in 2-methoxyethanol (2 mL) and refluxed for 3 hours. The solvent was then removed, and the residue purified on silica gel by flash chromatography using dichloromethane/methanol (0-40%), to give a yellow powder (0.20 g, 52 %)

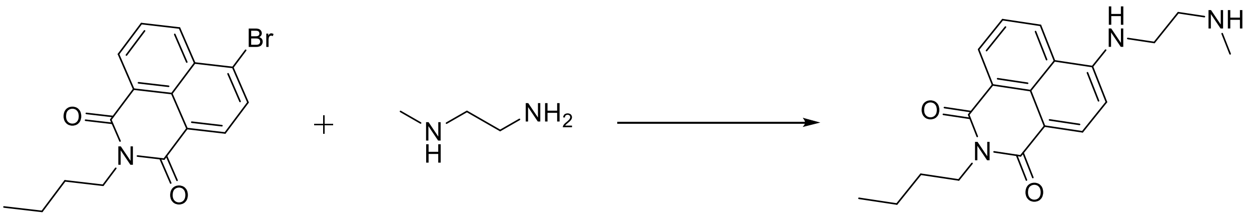

Scheme S2. The synthesis of Intermediate **1b**

^{1}H NMR (400 MHz, DMSO-d$_6$, 25 °C): δ 8.71 (dd, J = 8.5, 1.2 Hz, 1H), 8.45 (dd, J = 7.3, 1.0 Hz, 1H), 8.29 (d, J = 8.5 Hz, 1H), 7.79 (t, J = 5.6 Hz, 1H), 7.72 (dd, J = 8.4, 7.3 Hz, 1H), 6.89 (d, J = 8.6 Hz, 1H), 4.05 – 3.97 (m, 2H), 3.71 (q, J = 6.0 Hz, 2H), 3.26 (t, J = 6.1 Hz, 2H), 1.64 – 1.52 (m, 2H), 1.40 – 1.26 (m, 2H), 0.91 (t, J = 7.4 Hz, 3H).
^{13}C NMR (101 MHz, DMSO-d$_6$, 25 °C): δ 163.7, 162.9, 150.0, 134.0, 130.8, 129.2, 129.0, 124.5, 121.9, 120.4, 108.8, 104.1, 46.40, 32.65, 29.80, 19.82, 13.75.
ESI-MS: $[M + H]^+$ $C_{19}H_{23}N_3O_2$ calc. 326.1886, found 326.1875

Step 3. Synthesis of (2-(((2-((2-butyl-1,3-dioxo-2,3-dihydro-1H-benzo[de]isoquinolin-6-yl)amino)ethyl)(methyl)amino)methyl)phenyl)boronic acid (Compound 1)

Intermediate **1b** (0.1 g, 0.30 mmol), 2-bromophenylboronic acid (0.09 g, 0.30 mmol), and triethylamine (0.03 g ,0.30 mmol) were dissolved in acetonitrile (10 mL) and stirred for 1 hour at room temperature, after which time a precipitate crashed out. The precipitate was the collected by vacuum filtration and washed with acetonitrile (15 mL). This afforded the desired product as a yellow powder (0.09 g, 65 %). The analogous protocol was performed using 2-bromophenylboronic acid pinacol ester (0.30 mmol), or by treating compound **1** with excess pinacol, followed by semi prep HPLC purification. Compound 1.BPin was isolated in analytical scale (<5 mg) and characterised by mass spectrometry

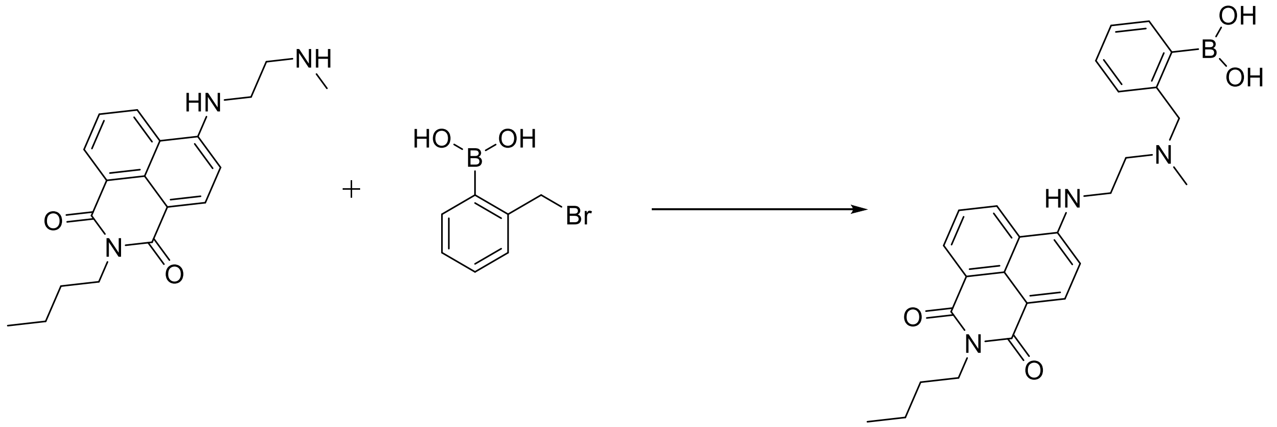

Scheme S3. The synthesis of Compound **1**

Characterisation data for Compound **1**:

^{1}H NMR (400 MHz, DMSO-d$_6$, 25 ˚C): δ 8.39 (dd, J = 8.7, 1.1 Hz, 1H), 8.29 (dd, J = 7.3, 1.0 Hz, 1H), 7.92 (d, J = 8.4 Hz, 1H), 7.57 – 7.45 (m, 3H), 7.20 – 7.11 (m, 2H), 6.97 (td, J = 7.4, 1.2 Hz, 1H), 6.48 (d, J = 8.6 Hz, 1H), 4.40 (s, 1H), 4.02 – 3.94 (m, 2H), 3.64 – 3.45 (m, 2H), 2.98 (d, J = 8.1 Hz, 2H), 2.48 (d, J = 1.9 Hz, 3H), 1.60 (dq, J = 14.9, 7.7 Hz, 2H), 1.37 (hept, J = 7.6 Hz, 2H), 0.96 (q, J = 7.0 Hz, 3H).

^{13}C NMR (101 MHz, DMSO-d$_6$, 25 ˚C): 163.5, 162.7, 149.8, 141.5, 133.7, 130.9, 130.3, 129.0, 128.0, 127.1, 126.1, 124.7, 124.0, 121.6, 119.9, 107.9, 103.5, 60.8, 56.0, 53.2, 42.4, 39.1, 29.8, 19.8, 18.6, 13.7.

ESI-MS: $[M + H]^+$ $C_{26}H_{30}BN_3O_4$ calc. 460.2406, found 460.2399

HPLC: 6.11 min

2.2 Synthesis of Compound 2.

Step 1. Synthesis of 6-bromo-2-propyl-1H-benzo[de]isoquinoline-1,3(2H)-dione (Intermediate 2a)

4-Bromo-1,8-naphthalic anhydride (1.0 g, 3.6 mmol) and *n*-propylamine (0.3 mL, 3.6 mmol) were dissolved in ethanol (50 mL), heated to at reflux for 6 six hours under a flow of nitrogen. After concentrating the reaction mixture under reduced pressure, the resulting suspension was transferred into ice water (100 mL). The crude product was filtered and purified by column chromatography. The desired compound, intermediate **2a,** was obtained as a grey solid (1.03 g, yield: 89%).

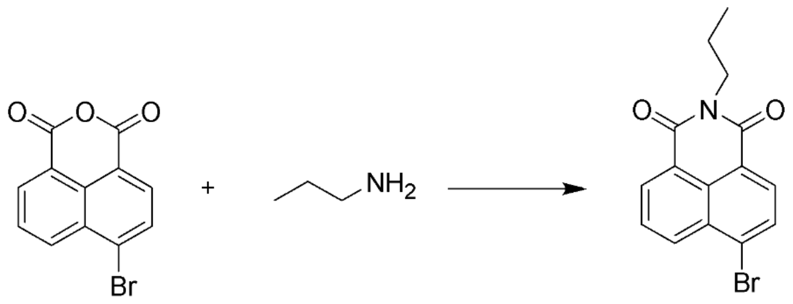

Scheme S4. The synthesis of Intermediate **2a**

^{1}H NMR (300 MHz, $CDCl_3$): 8.50 (d, J = 7.29 Hz, 1H), 8.39 (d, J = 8.7 Hz, 1H), 8.25 (d, J = 7.8 Hz, 1H), 7.89 (d, J = 7.5 Hz, 1H), 7.71 (t, J = 7.5 Hz, 1H), 4.03 (t, J = 7.5 Hz, 2H), 1.73–1.61 (m, 2H) and 0.93 (t, J = 7.5 Hz, 3H).
^{13}C NMR (75.5 MHz, $CDCl_3$): 163.5, 163.5, 133.1, 131.9, 131.1, 131.0, 130.5, 130.1, 128.8, 128.0, 123.0, 122.2, 42.1, 21.4, 11.6
ESI-MS $[M + H]^+$ $C_{15}H_{13}BrNO_2$ calc. 318.0124, found 319.1413

Step 2. Synthesis of 6-hydrazineyl-2-propyl-1H-benzo[de]isoquinoline-1,3(2H)-dione (Intermediate 2b)

Intermediate **2a** (1.03 g, 3.24 mmol) and hydrazine hydrate (0.3 ml, 0.31 g, 9.5 mmol) were dissolved in 2-methyoxylethanol (4 mL) and heated to reflux for 3 hours. After cooling to room temperature, precipitation was collected and washed with ethanol. Silica chromatography (95% MeOH in DCM) was employed to purify and a yellow solid **2b** was obtained (0.62 g, 71.3%).

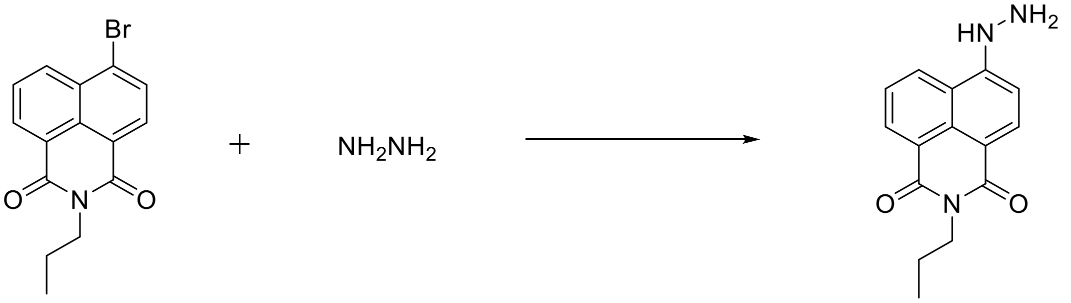

Scheme S5. The synthesis of Intermediate **2b**

^{1}H NMR (300 MHz, DMSO-d_6): 9.09 (s, 1H), 8.59 (d, J = 7.8 Hz, 1H), 8.40 (d, J = 7.8 Hz, 1H), 8.28 (d, J = 9 Hz, 1H), 7.60 (t, J = 7.2 Hz, 1H), 7.23 (d, J = 9 Hz, 1H), 3.96 (t, J = 6 Hz, 2H), 1.68–1.55 (m, 2H), 0.90 (t, J = 6 Hz, 3H)
^{13}C NMR (75.5 MHz, DMSO-d_6): 164.2, 163.3, 153.5, 134.6, 130.9, 129.7, 128.6, 124.5, 122.1, 118.8, 107.7, 104.4, 41.2, 21.3, 11.8
ESI-Mass $[M + Na]^+$ $C_{15}H_{15}N_3NaO_2^+$ calc. 292.1056, found 292.1031

Synthesis of 2-(1,3-dioxo-2-propyl-2,3-dihydro-1H-benzo[de]isoquinolin-6-yl)-N-(3-(4,4,5,5-tetramethyl-1,3,2-dioxaborolan-2-yl)phenyl)hydrazine-1-carbothioamide (Compound 2)

A mixture of Intermediate **2b** (0.202 g, 0.71 mmol) and 2-(3-isothiocyanatophenyl)-4,4,5,5-tetramethyl-1,3,2-dioxaborolane (0.186 g, 0.71 mmol) in DMF (20 mL) was left to stir at r.t. for 2 hours. The solid products were filtered and washed with diethyl ether to get the desired compound **2** in quantitative yield, which was then recrystalised further from dioxane.

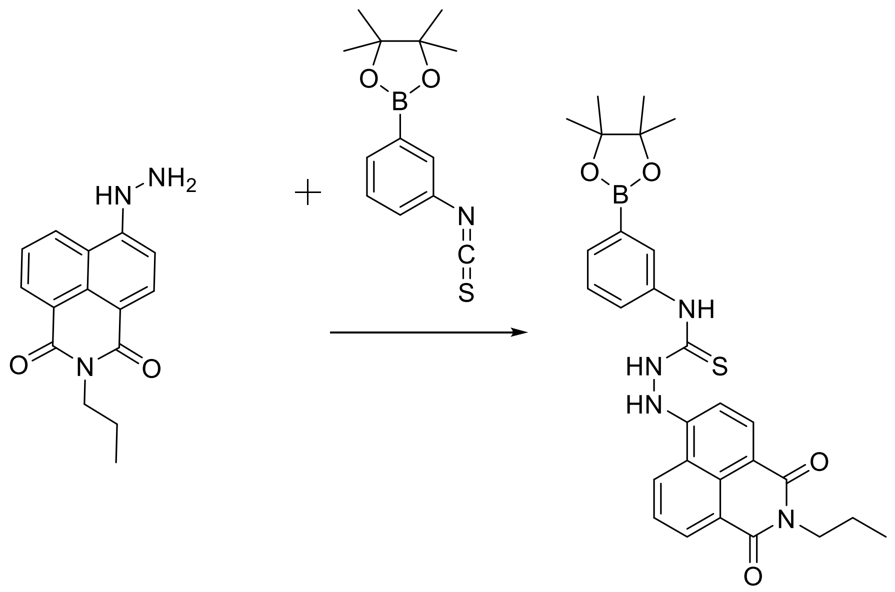

Scheme S6. The synthesis of Compound **2**

^{1}H NMR (250 MHz, CD_3OD) δ 8.32 (d, *J* = 8.5Hz, 1H), 8.19 (d, *J* = 8.3 Hz, 1H), 8.08 (d, *J* = 7.5 Hz, 1H), 7.87 (s, 1H), 7.73 (d, *J* = 7.9 Hz, 1H), 7.57 (d, *J* = 7.6 Hz, 1H), 7.46 (td, *J* = 7.0 Hz, 1H), 7.35 (td, *J* = 8.0 Hz, 1H), 7.05 (d, *J* = 8.5 Hz, 1H), 3.92 (t, 2H), 1.60 – 1.45 (m, 2H), 1.29 (m, 14H), 0.90 (td, *J* = 7.9 Hz, 3H).
^{13}C NMR (75.5 MHz, CD_3OD): 164.0, 163.7, 138.2, 133.4, 131.8, 130.6, 127.4, 124.8, 121.7 116.9, 112.6, 105.94, 83.72, 47.2, 46.9, 46.5, 39.5, 29.7, 23.7, 23.6, 19.8, 12.7
ESI-Mass $[M – H]^-$ $C_{29}H_{32}BN_4O_4S^-$ calc.543.2243, found 543.2258
HPLC: 7.89 min

2.2 Synthesis of Compound 3

Alternative method for the synthesis of 6-bromo-2-propyl-1H-benzo[de]isoquinoline-1,3(2H)-dione (3a):
4-bromo-1,8-naphthalic anhydride (1.0 g, 3.6 mmol) and *n*-propylamine (0.3 mL, 3.6 mmol) were dissolved in ethanol (30 mL) and heated to reflux for 6 hours. The suspension was then cooled to room temperature and precipitate collected and washed with ethanol to obtain a grey solid (1.03 g, 89 %).

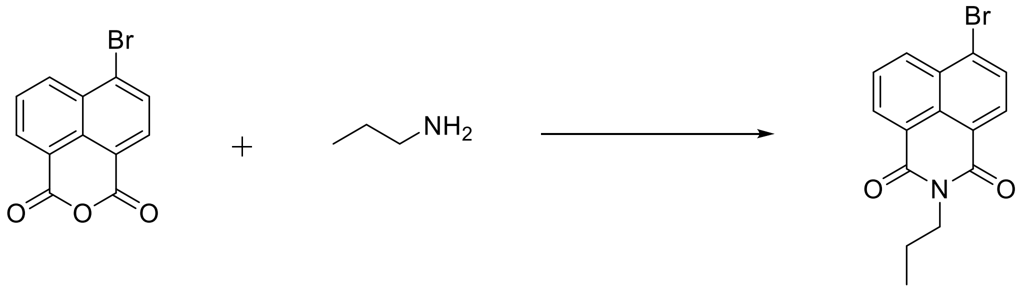

Scheme S7. The synthesis of Intermediate **3a**

^{1}H NMR (400 MHz, DMSO-d$_6$, 25 ˚C): δ 8.51 (dd, *J* = 7.2, 13.7 Hz, 2H), 8.29 (d, *J* = 7.8 Hz, 1H), 8.18 (d, *J* =7.8 Hz, 1H), 7.96 (t, *J* = 7.8 Hz, 1H), 3.98 (t, *J* = 7.4 Hz, 2H), 1.65 (h, *J* = 7.4 Hz, 2H), 0.92 (t, *J* = 7.4 Hz, 3H).
^{13}C NMR (101 MHz, DMSO-d$_6$, 25 ˚C): δ 162.9, 162.8, 132.5, 131.5, 131.2, 130.9, 129.8, 129.1, 128.8, 128.3, 122.7, 122.0, 41.3, 20.8, 11.3.
ESI-MS: $[M + H]^+$ $C_{15}H_{13}BrNO_2$ calc. 319.1728, found 319.1657

Synthesis of 6-hydrazineyl-2-propyl-1H-benzo[de]isoquinoline-1,3(2H)-dione (3b):
Intermediate **3a** (1.03 g, 3.24 mmol) and hydrazine hydrate (0.306 g, 9.5 mmol) were dissolved in 2-methoxyethanol (4 mL) and refluxed for 3 hours. The mixture was then cooled to room temperature and the precipitate collected and washed with cold ethanol. Product was obtained as an orange solid (0.62 g,71.3 %).

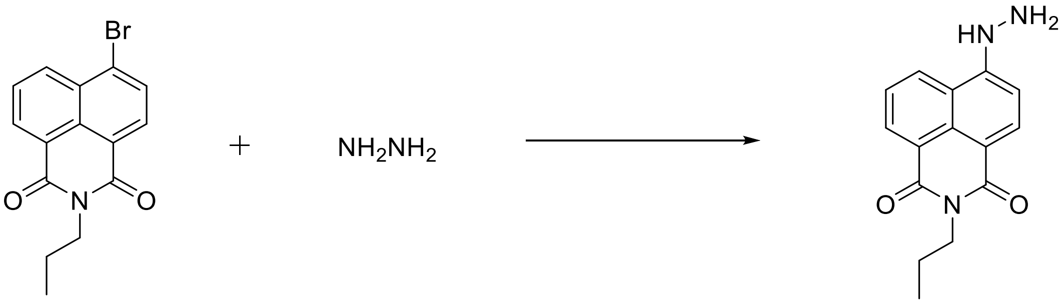

Scheme S8. The synthesis of Intermediate **3b**

^{1}H NMR (400 MHz, DMSO-d$_6$, 25 ˚C): δ 9.09 (s, 1H, NH), 8.58 (d, *J* = 8.5 Hz, 1H), 8.40 (d, *J* =7.3 Hz, 1H), 8.27 (d, *J* = 8.5 Hz, 1H), 7.61 (t, *J* = 7.4 Hz, 1H), 7.23 (d, *J* = 8.6 Hz , 2H), 4.66 (s, 2H, NH_2), 3.96 (t, *J* = 7.4 Hz, 2H), 1.62 (h, *J* = 7.4 Hz , 2H), 0.90 (t, *J* = 7.4 Hz, 3H).
^{13}C NMR (101 MHz, DMSO-d$_6$, 25 ˚C): δ 163.7, 163.0, 153.1, 134.2, 130.6, 129.3, 128.2, 124.1, 121.7, 118.4, 107.3, 104.0, 40.7, 21.0, 11.4.
ESI-MS: $[M + Na]^+$ $C_{15}H_{15}N_3O_2$ calc. 292.1062, found 292.1013

Synthesis of (E)-2-propyl-6-(2-(4-(4,4,5,5-tetramethyl-1,3,2-dioxaborolan-2-yl)benzylidene)hydrazineyl)-1H-benzo[de]isoquinoline-1,3(2H)-dione (Compound 3-BPin)

Intermediate **3b** (0.15 g, 0.56 mmol) and 4-formylbenzoic boronic acid pinacol ester (0.14 g, 0.6 mmol), were dissolved in ethanol and heated to reflux for 6 hours. The reaction was cooled

over ice and the resultant precipitate collected and washed with cold ethanol. The product was obtained as an orange solid (0.114 g, 40 %).

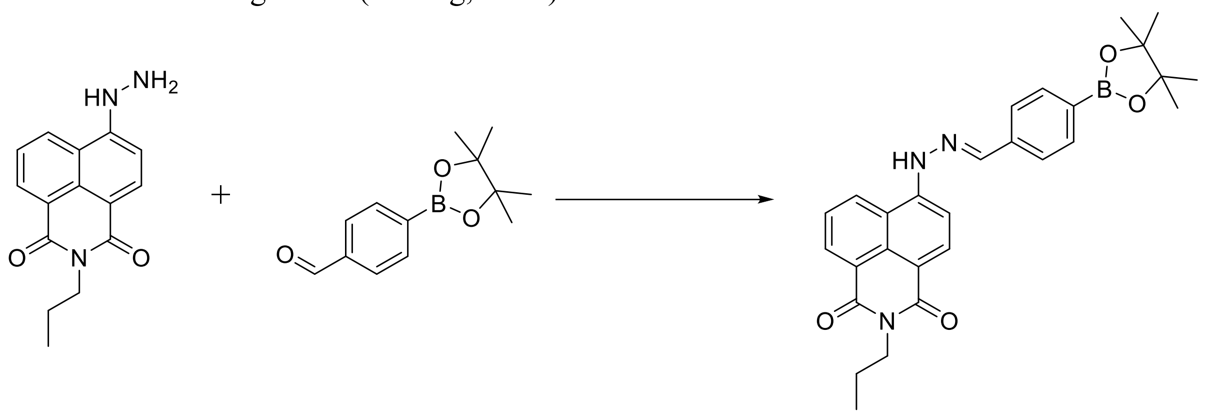

Scheme S9. The synthesis of Compound **3-BPin**

^{1}H NMR (400 MHz, DMSO-d$_6$, 25 ˚C): δ 11.5 (s, 1H, NH), 8.77 (d, *J* = 8.5 Hz), 8.46 (d, *J* =7.4 Hz, 1H), 8.42 (s, 1H), 8.37 (d, *J* = 8.5 Hz, 1H), 7.80-7.71 (m , 6H), 3.97 (t, *J* = 7.4 Hz, 2H), 1.63 (h, *J* = 7.4 Hz , 2H), 1.32 (s, 12H), 0.91 (t, *J* = 7.4 Hz, 3H).
^{13}C NMR (101 MHz, DMSO-d$_6$, 25 ˚C): δ 162.9, 146.2, 143.5, 137.3, 134.8, 133.4, 130.8, 129.1, 128.2, 126.0, 125.0, 122.0, 118.7, 111.3, 107.1, 83.8, 40.9, 24.7, 20.9, 11.4.
ESI-MS: $[M + H]^+$ $C_{28}H_{30}BN_3O_4$ calc. 484.2403, found 484.2407
HPLC: 9.51 min

2.3 Synthesis of Compound III-BPin:

The synthesis of **Compound III-BPin** was carried out according to the literature method. [10]

2-propyl-6-(2-(2-(4,4,5,5-tetramethyl-1,3,2-dioxaborolan-2-yl)benzylidene)hydrazinyl)-*1H*-benzo[*de*]isoquinoline-1,3(*2H*)-dione (Compound III)

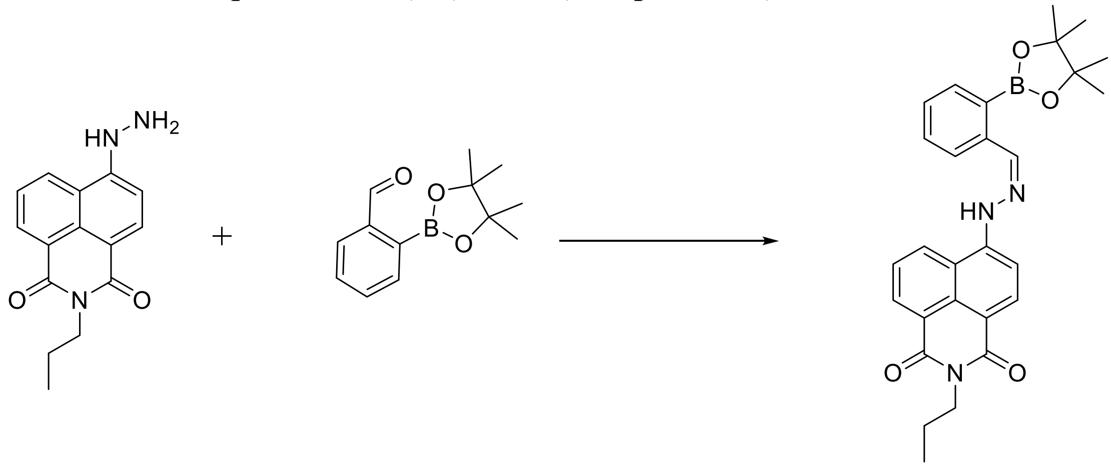

Scheme S10. The synthesis of Compound **III-BPin**

Briefly, intermediate **2b**, (0.287g, 1.06 mmol) was dissolved in MeOH / DMF and to this 2-aldehyde-pinacol-boronate ester, (0.252g, 1.08 mmol) was added, followed by stirring at room temperature for 12 hours. Solid was precipitated out after pouring the mixture into an ice-water mixture. The crude product was first recrystallized then advanced purification by flash column chromatography followed, leading to the isolation of compound **III** as its pinacol-protected boronate (0.04g, 8% yield). [Ref 10]

^{1}H NMR (300 MHz, DMSO-d$_6$) 11.67 (s, NH, 1H), 8.98 (s, 1H), 8.49 (d, J = 7.2 Hz, 1H), 8.37 (d, J = 8.5 Hz, 1H), 8.15 (d, J = 7.8 Hz, 1H), 7.78 (m, 3H), 7.57 (t, J = 7.4 Hz, 1H), 7.41 (t, J = 7.3 Hz, 1H), 3.98 (d, J = 7.3 Hz, 2H), 1.63 (m, 2H), 1.36 (s, 12H), 0.93 (t, J = 7.4 Hz, 3H)
^{13}C NMR (75.5 MHz, DMSO-d$_6$) 164.0, 163.4, 147.0, 144.9, 140.3, 136.0, 133.8, 131.5, 131.3, 129.5, 129.2, 128.8, 125.6, 125.3, 122.3, 119.1, 111.5, 107.7, 84.3, 25.0, 21.3, 11.8
ESI MS $[M + H]^+$ $C_{28}H_{30}B_1N_3O_4$ Calc.484.2363, found 484.2343;
HPLC: Rt = 8.86 min

3. Spectroscopic data: isolated intermediates, Compounds 1-3 and III

Intermediate 1a

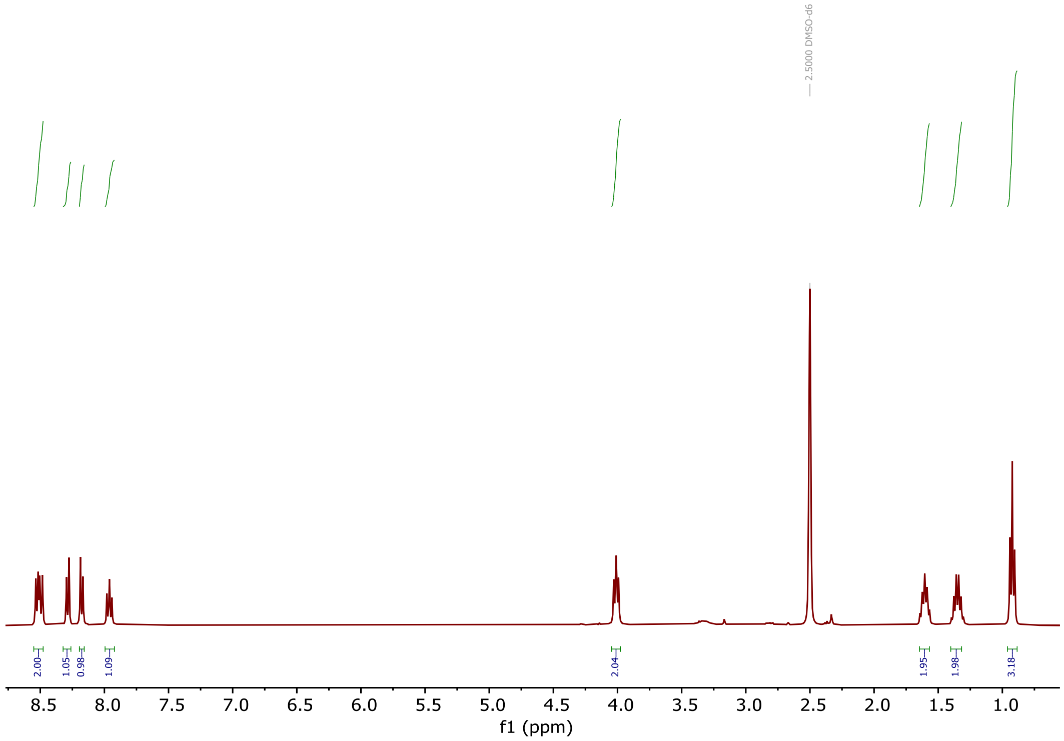

Figure S1. ^{1}H NMR (400 MHz, 25 ˚C, DMSO-d$_6$) of intermediate **1a**

Intermediate 1b

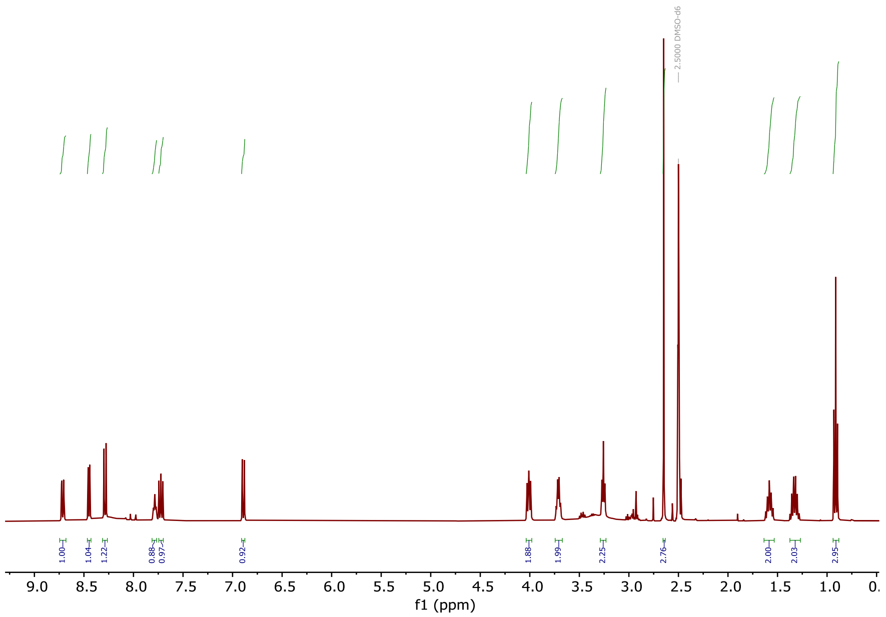

Figure S2. ^1H NMR (400 MHz, 25 ˚C, DMSO-d$_6$) of intermediate **1b**

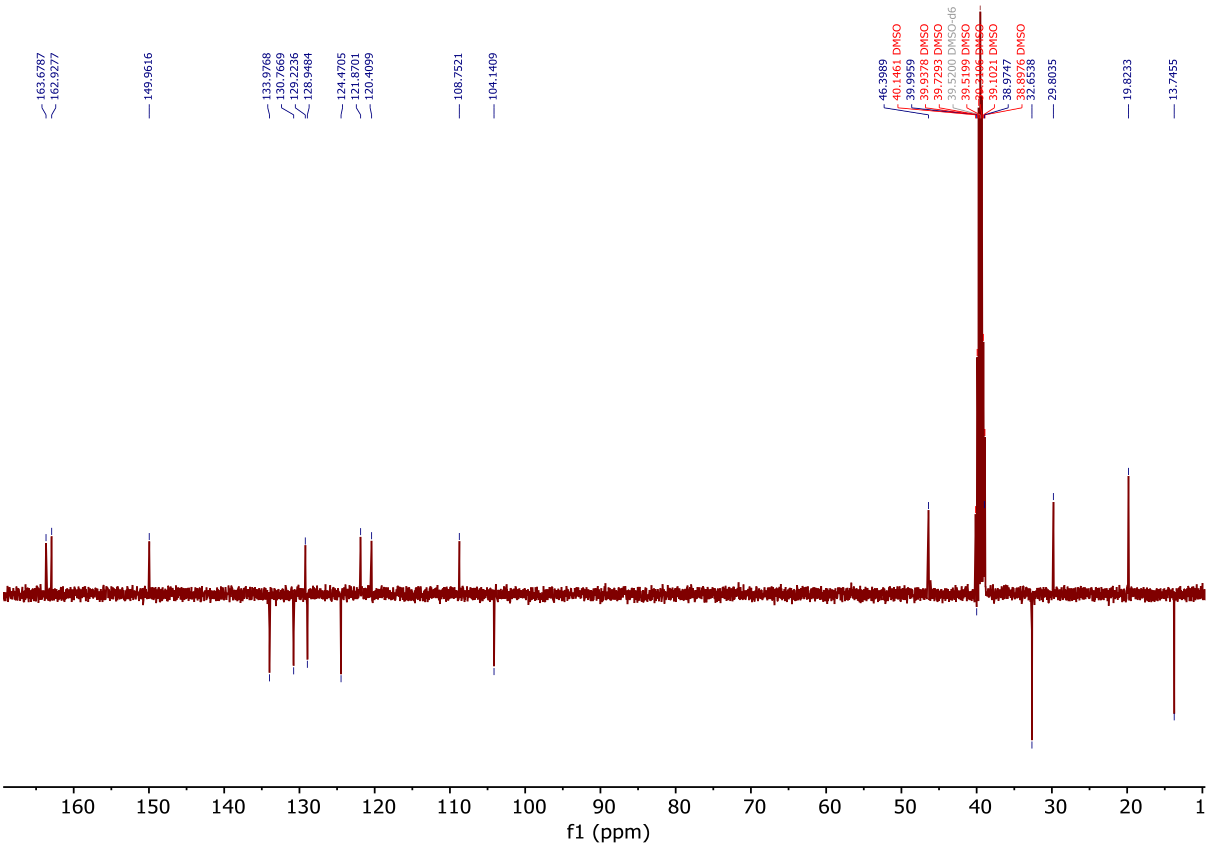

Figure S3. $^{13}C\{^1H\}$ NMR (DEPT) (101 MHz, 25 ˚C, DMSO-d$_6$) of intermediate **1b**

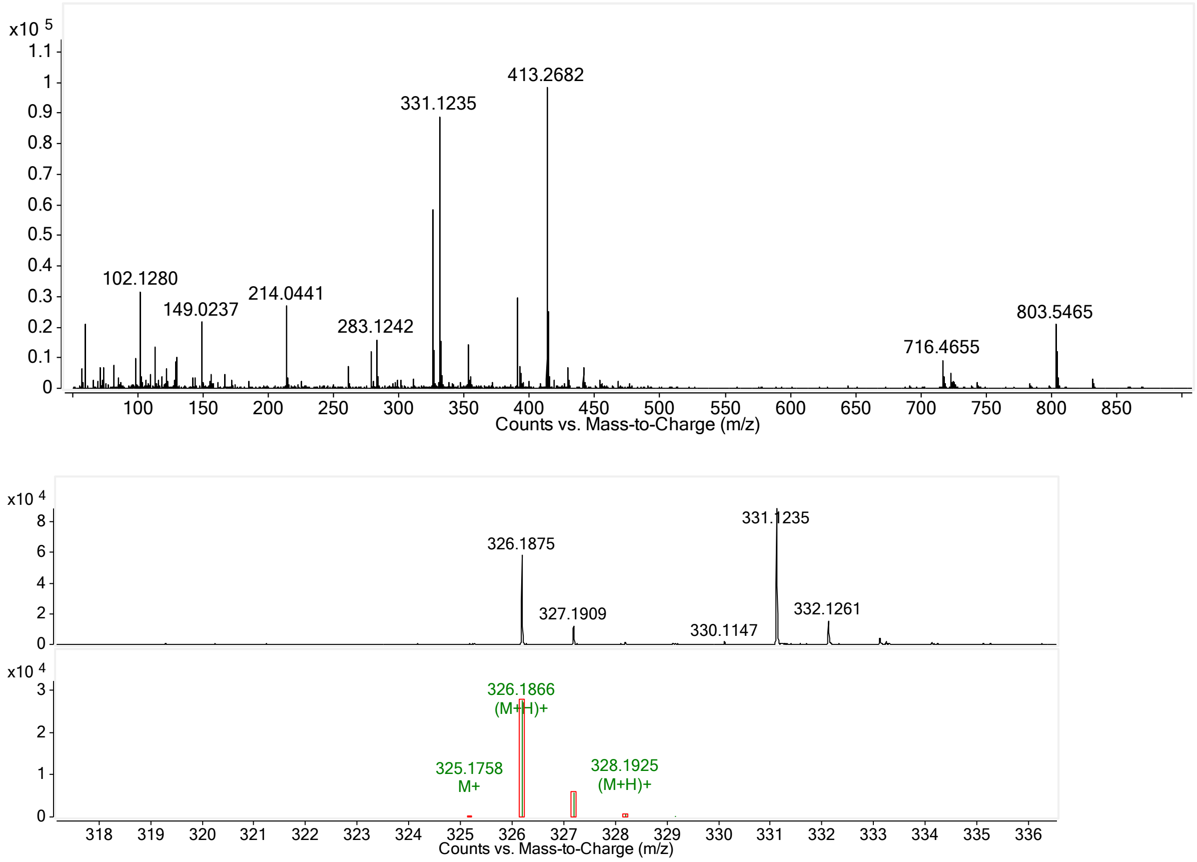

Figure S4. ESI-MS of intermediate **1b**

Compound 1

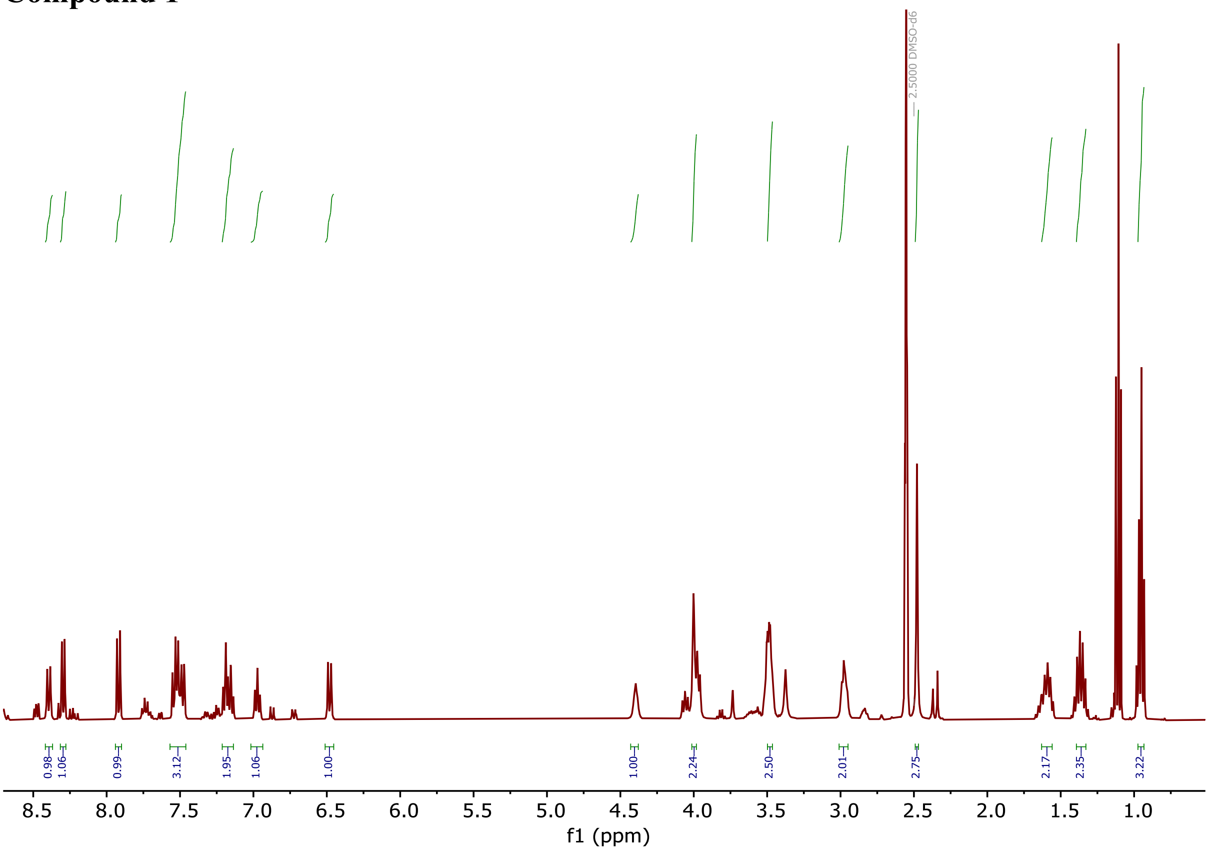

Figure S5. ^{1}H NMR (400 MHz, 25 °C, DMSO-d_6) of Compound **1.**

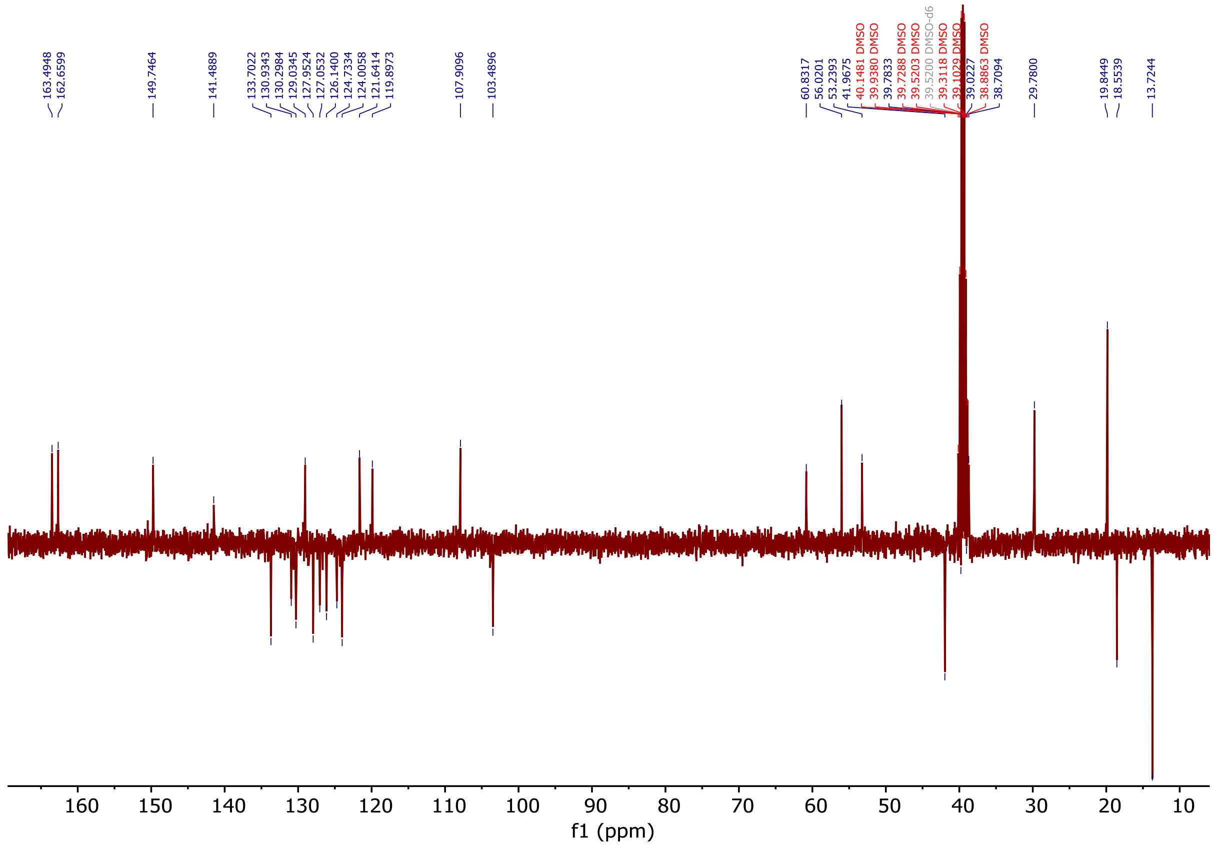

Figure S6. $^{13}C\{^{1}H\}$ NMR (DEPT) (101 MHz, 25 ˚C, DMSO-d_6) of compound **1.**

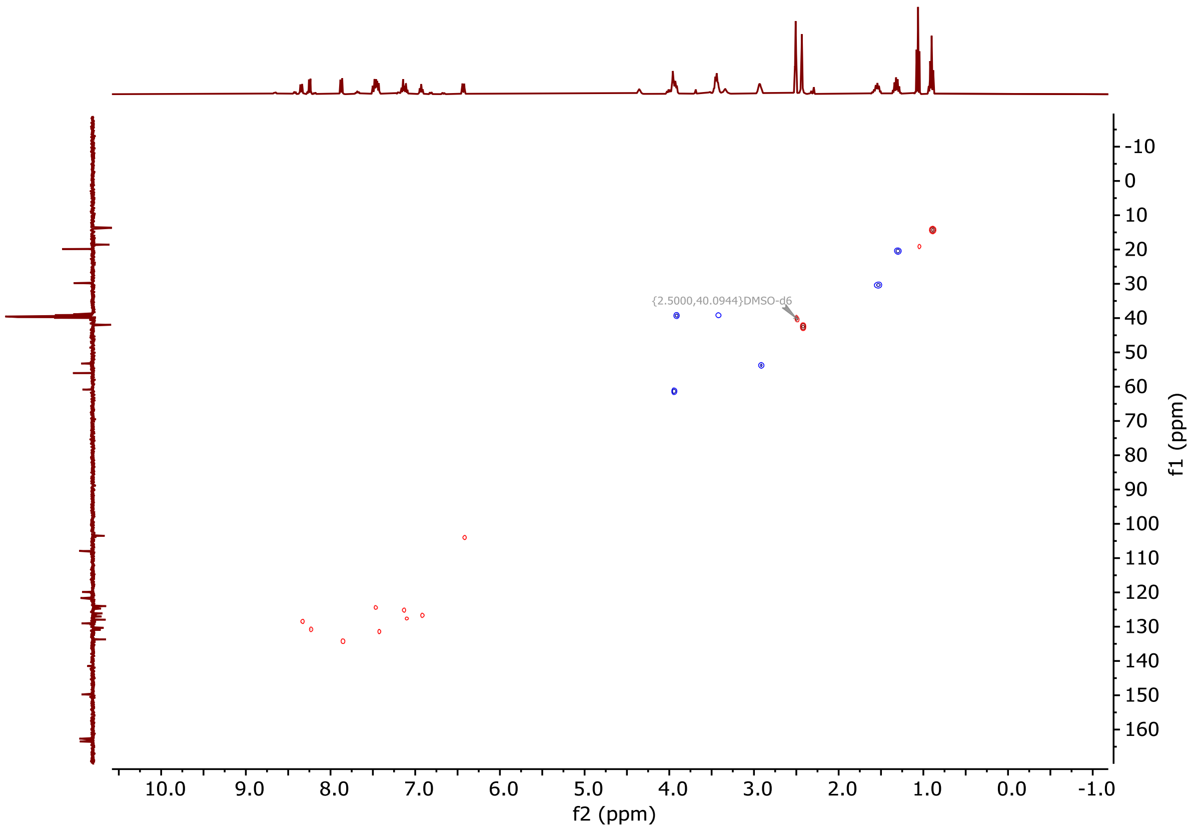

Figure S7. HSQC NMR (25 ˚C, DMSO-d_6) of compound **1.**

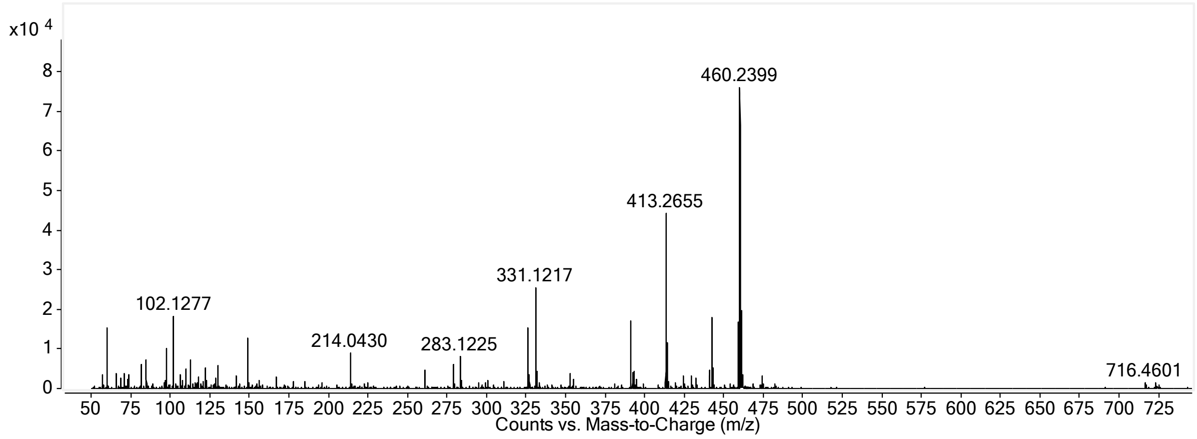

(a)

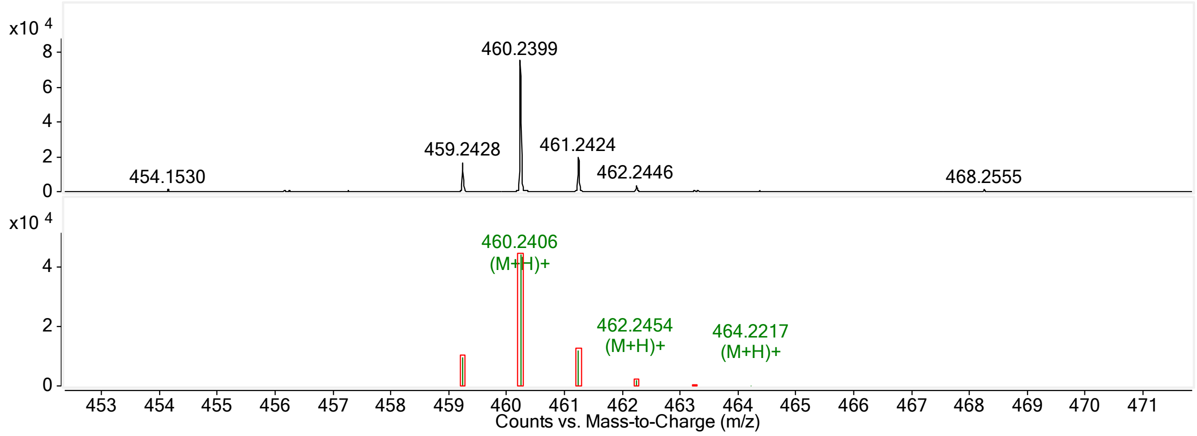

(b)

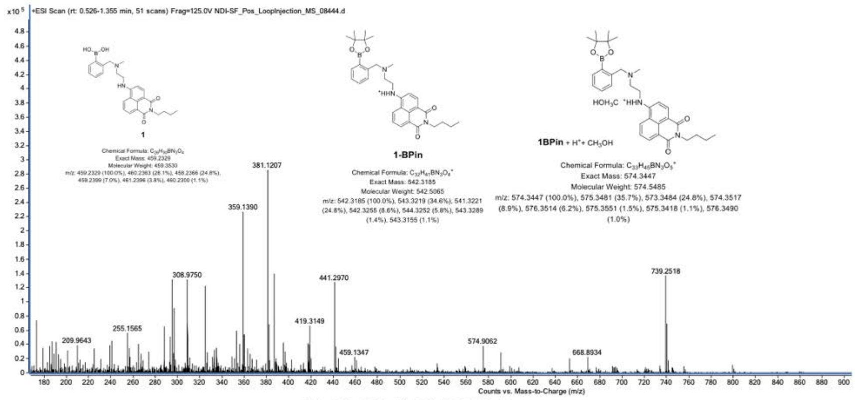

(M+CH_3OH+H): M+33.03349

(c)

Figure S8. (a-b) ESI$^+$-MS of Compound **1. (b)** ESI$^+$-MS of pinacol-protected sample of Compound **1.**

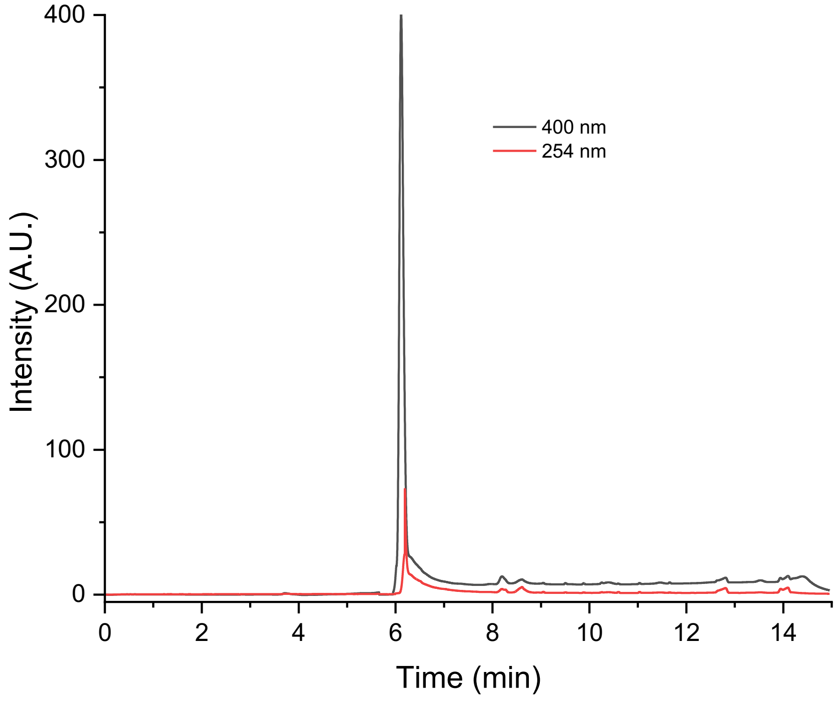

Figure S9. HPLC trace of Compound **1.**

Compound 2a

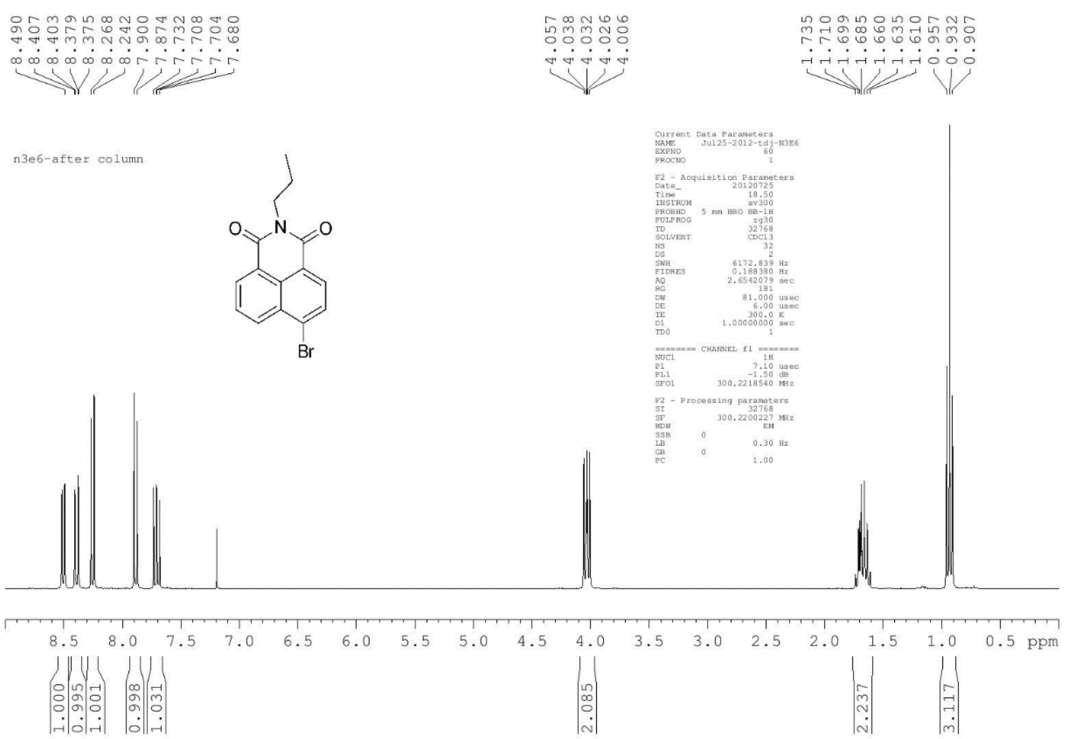

Figure S10. ^{1}H NMR (300 MHz, 25 ˚C, $CDCl_3$) of intermediate **2a**

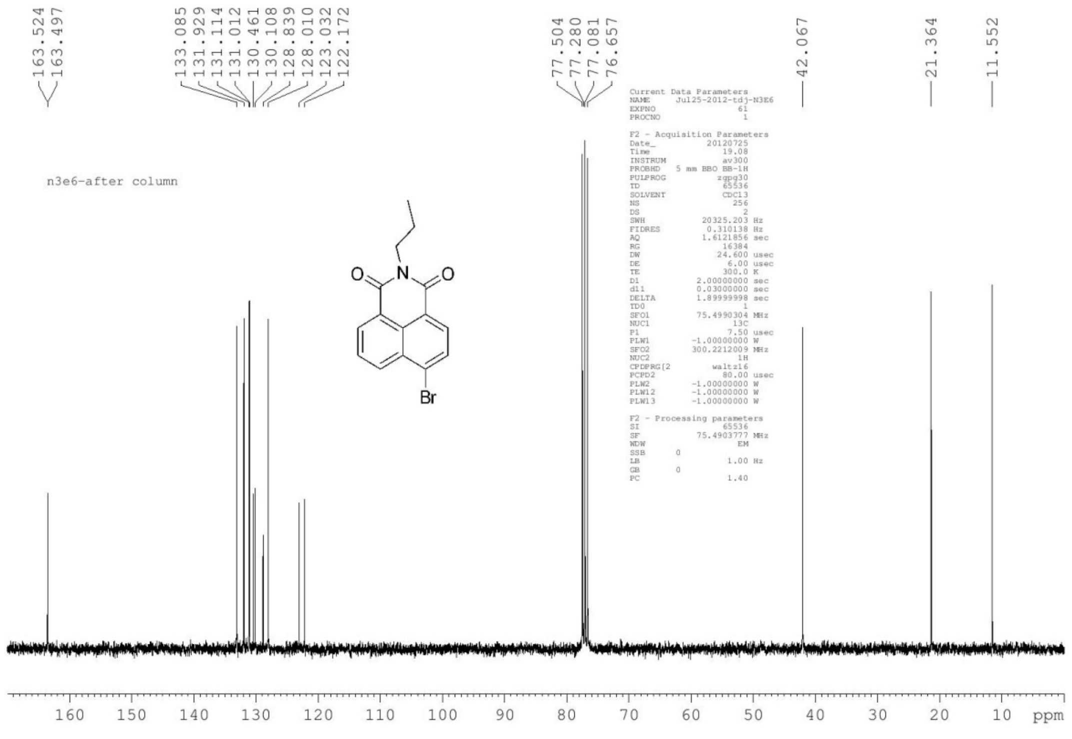

Figure S11. ^{13}C{^{1}H} NMR (75.5 MHz, 25 ˚C, DMSO-d_6) of intermediate **2a**

Intermediate 2b

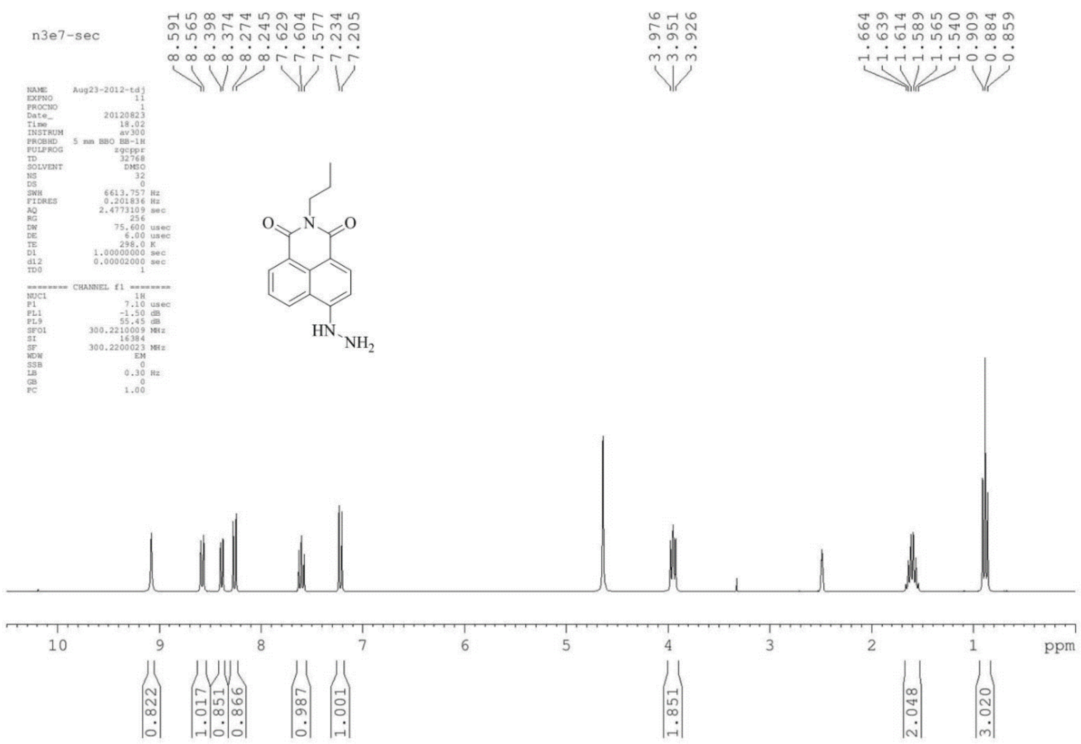

Figure S12. ^{1}H NMR (300 MHz, 25 ˚C, DMSO-d_6) of intermediate **2b**

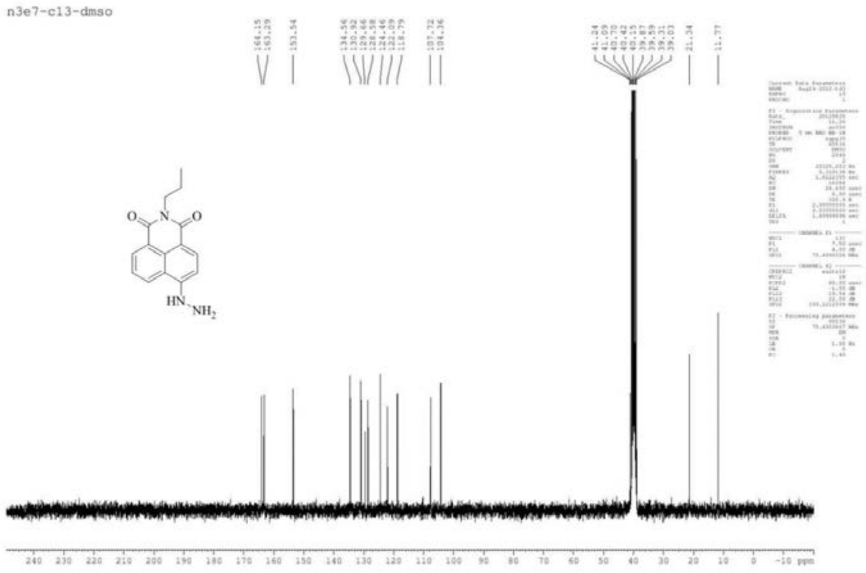

Figure S13. $^{13}C\{^{1}H\}$ NMR (75.5 MHz, 25 ˚C, DMSO-d_6) of intermediate **2b**

Compound 2-BPin

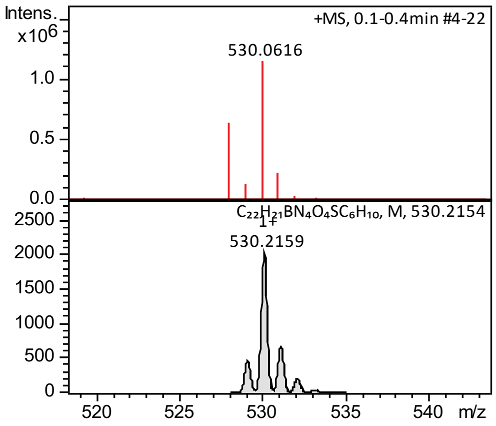

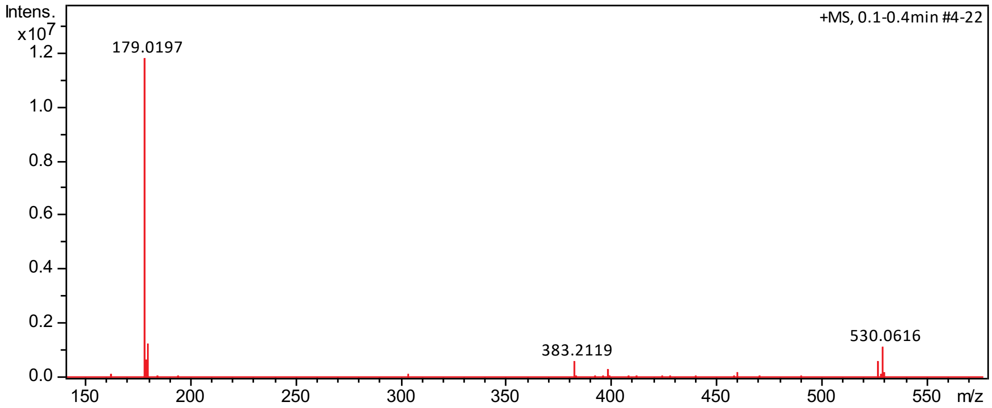

Figure S14. ESI-MS of Compound **2-BPin**

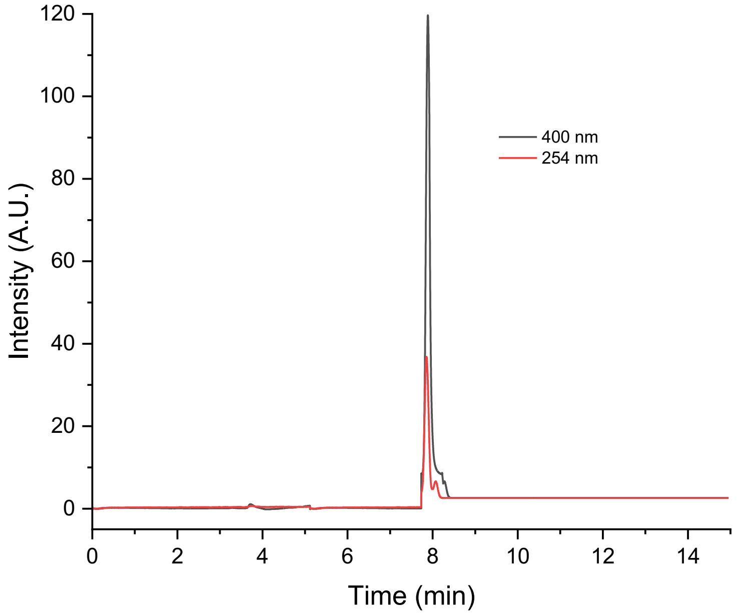

Figure S15. HPLC trace of Compound **2-BPin**

Intermediate 3a

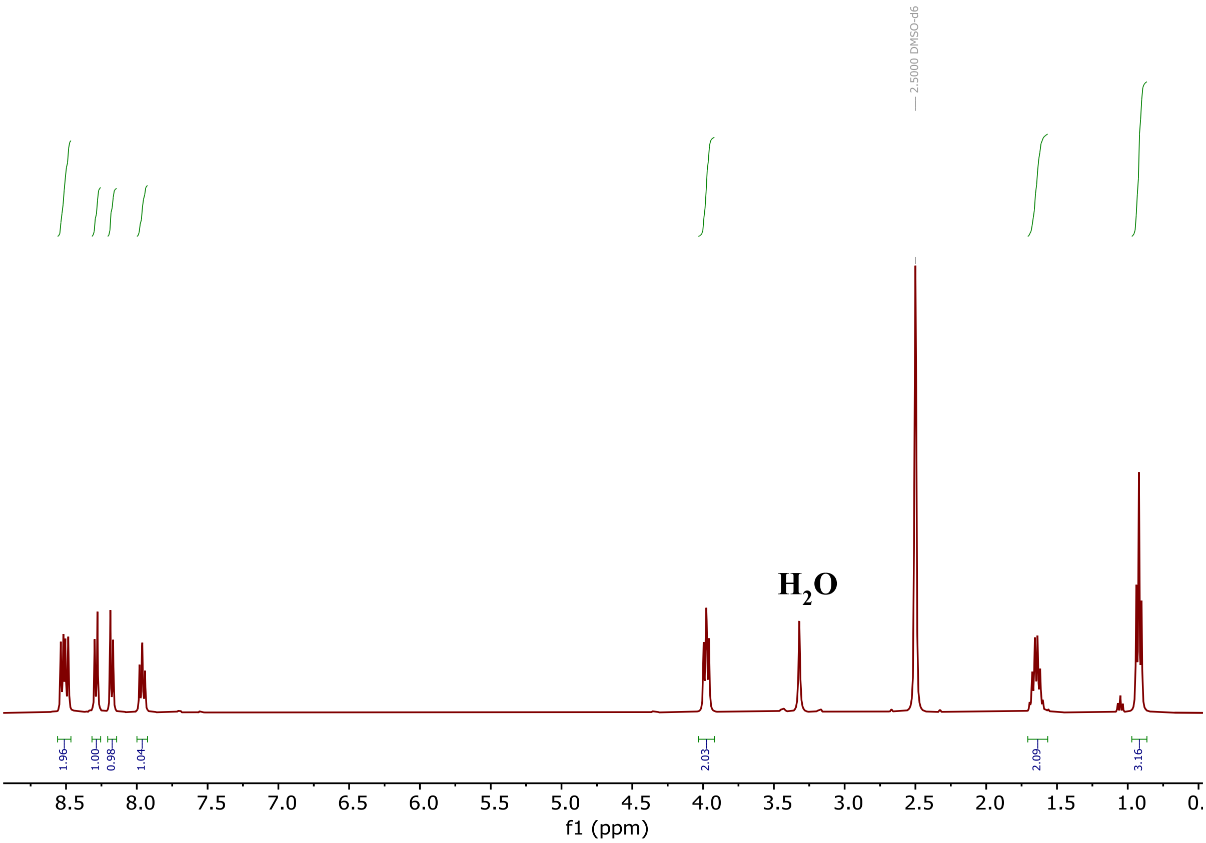

Figure S16. ^{1}H NMR (300 MHz, 25 ˚C, DMSO-d_6) of intermediate **3a**

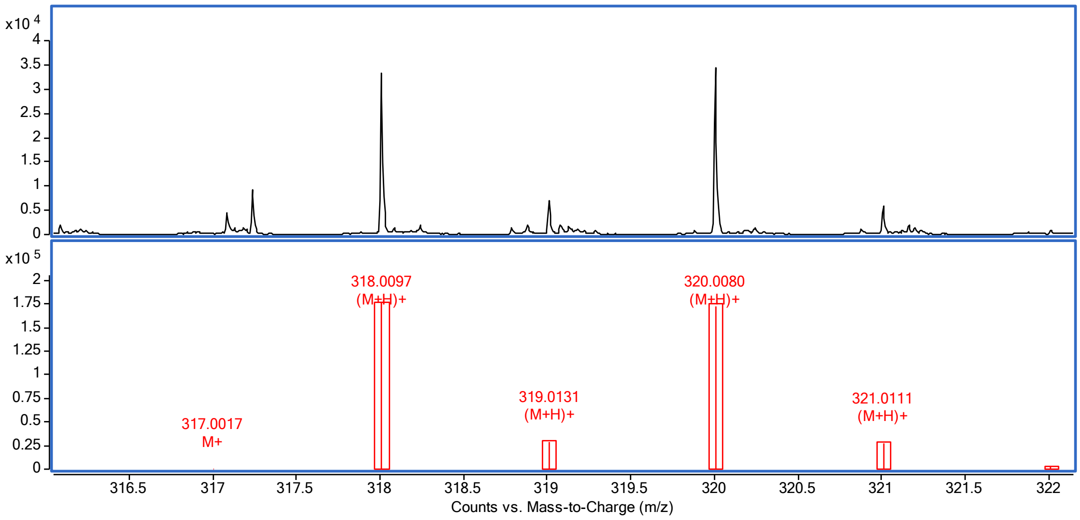

Figure S17. ESI-MS of Intermediate **3a**

Intermediate 3b

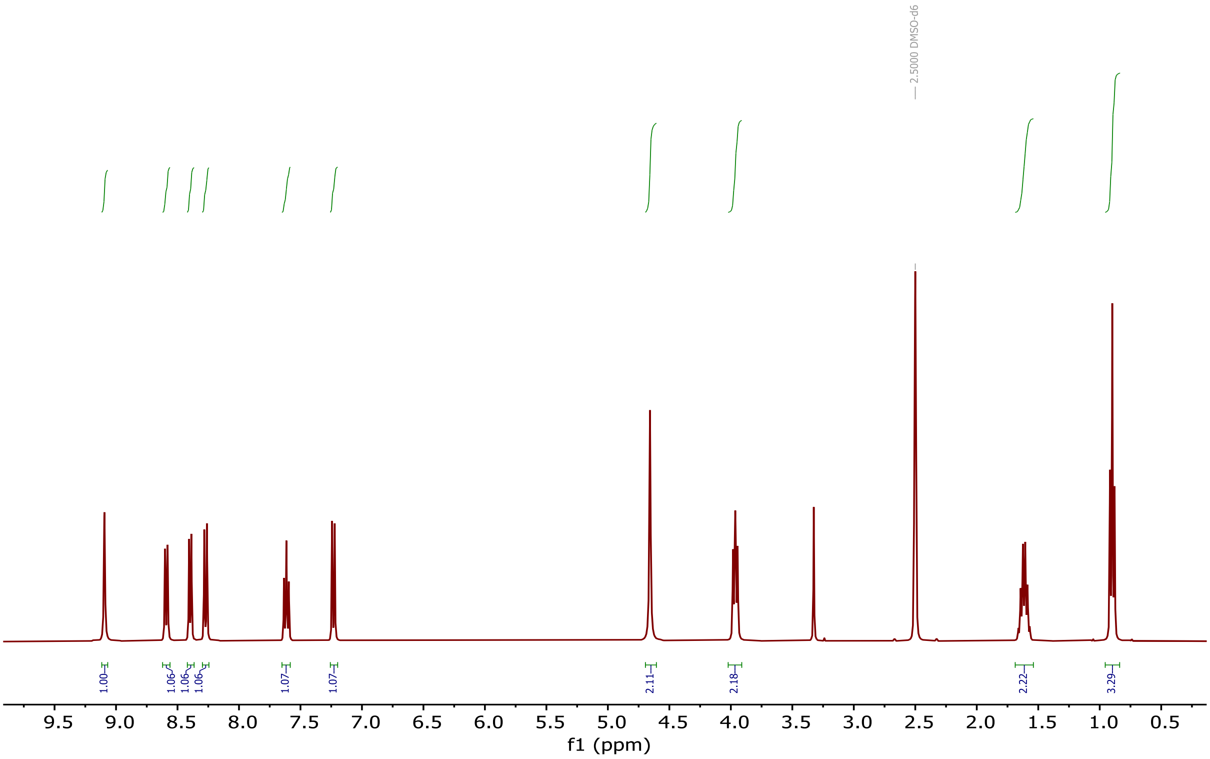

Figure S18. ^{1}H NMR (300 MHz, 25 ˚C, DMSO-d_6) of intermediate **3b**

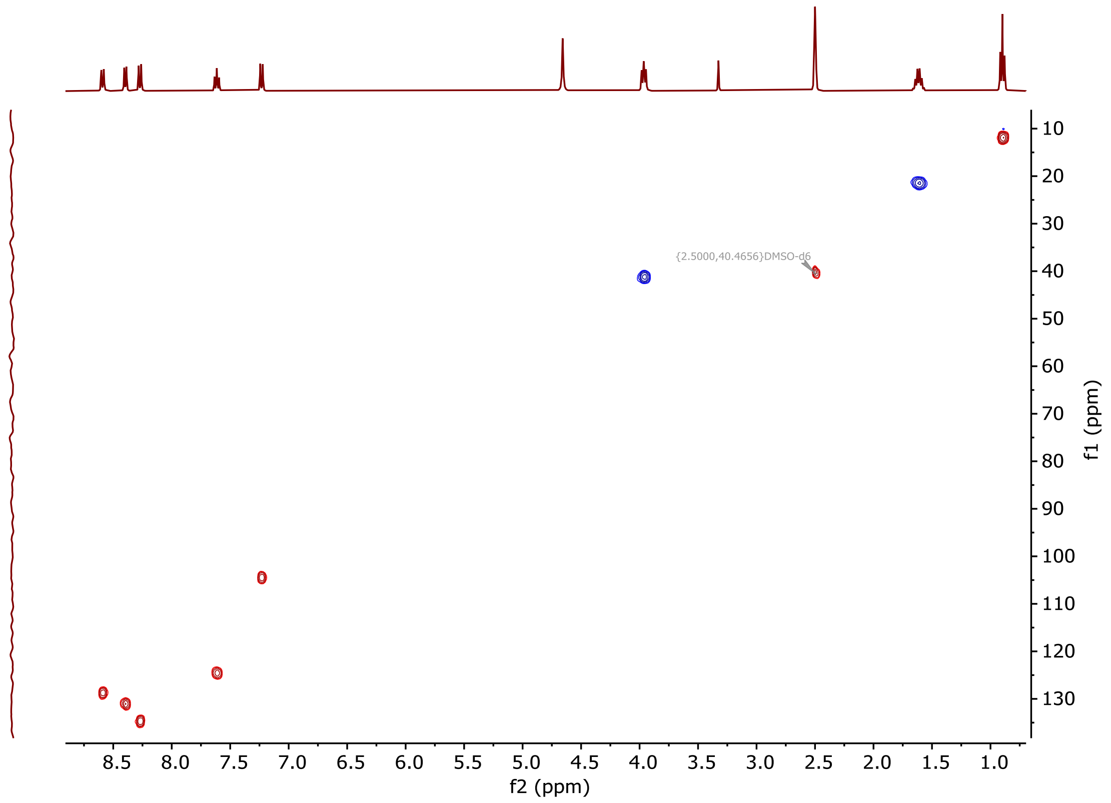

Figure S19. HSQC NMR (25 ˚C, DMSO-d$_6$) of intermediate **3b**

Compound 3

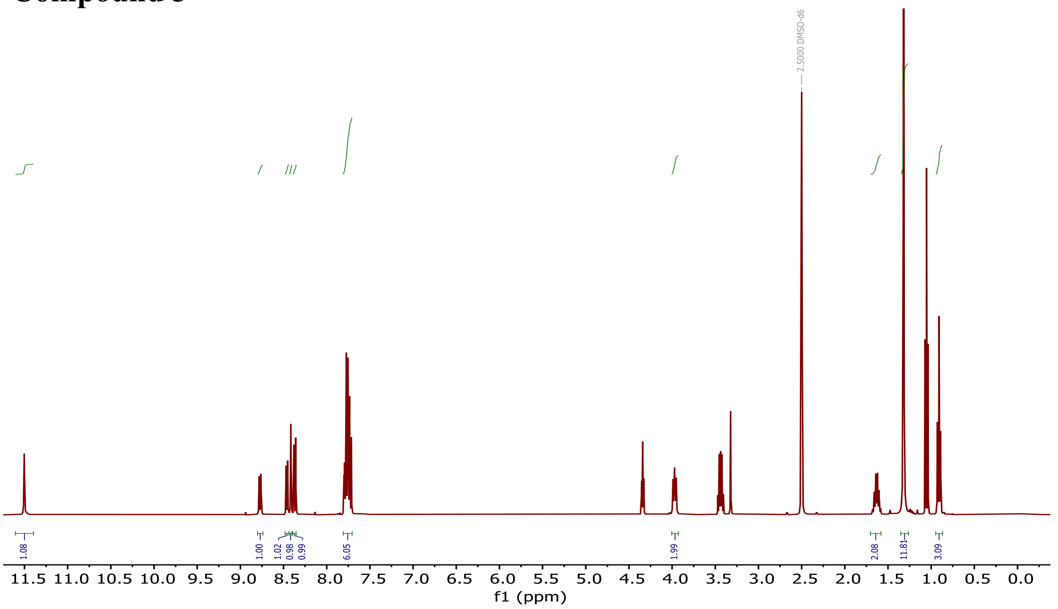

Figure S20. ^{1}H NMR (400 MHz, 25 ˚C, DMSO-d$_6$) of Compound **3-BPin**

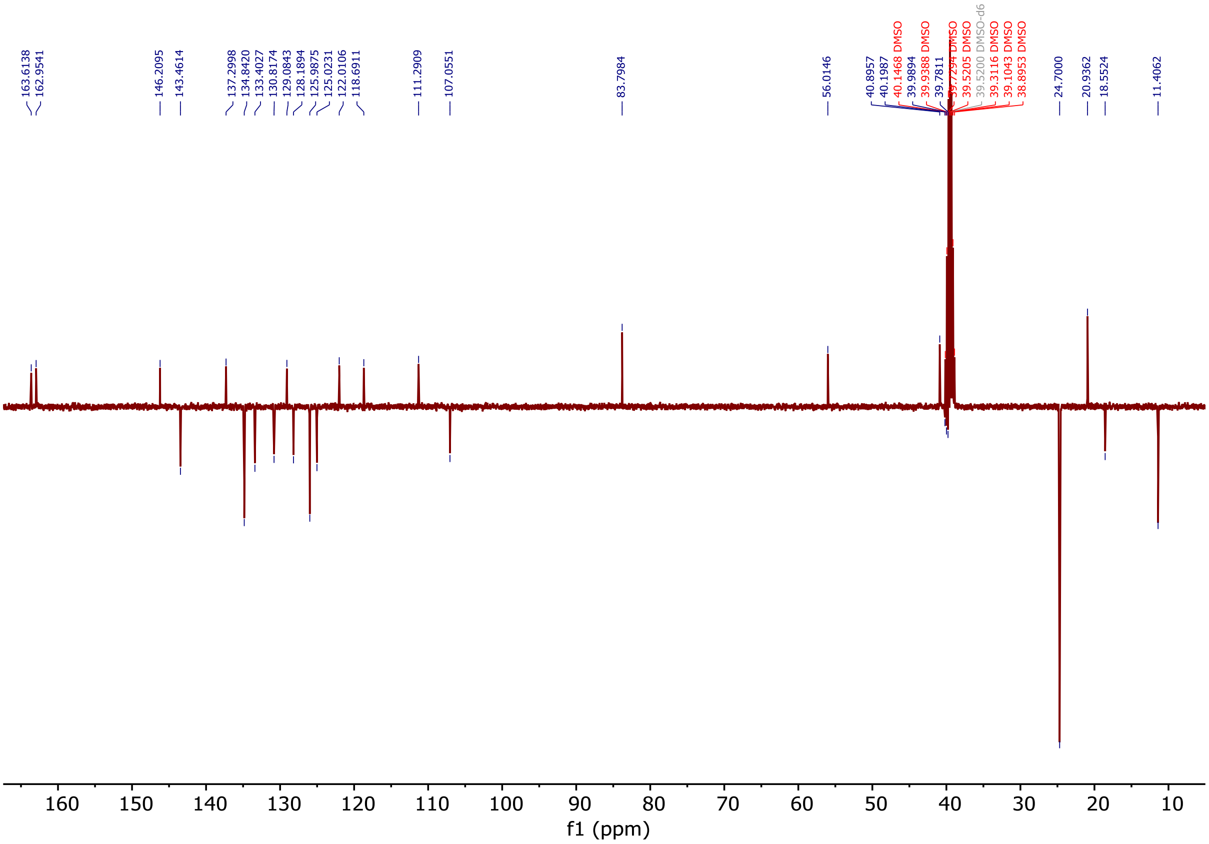

Figure S21. ^{13}C NMR (DEPT) (101 MHz, 25 ˚C, DMSO-d_6) of compound **3-BPin**

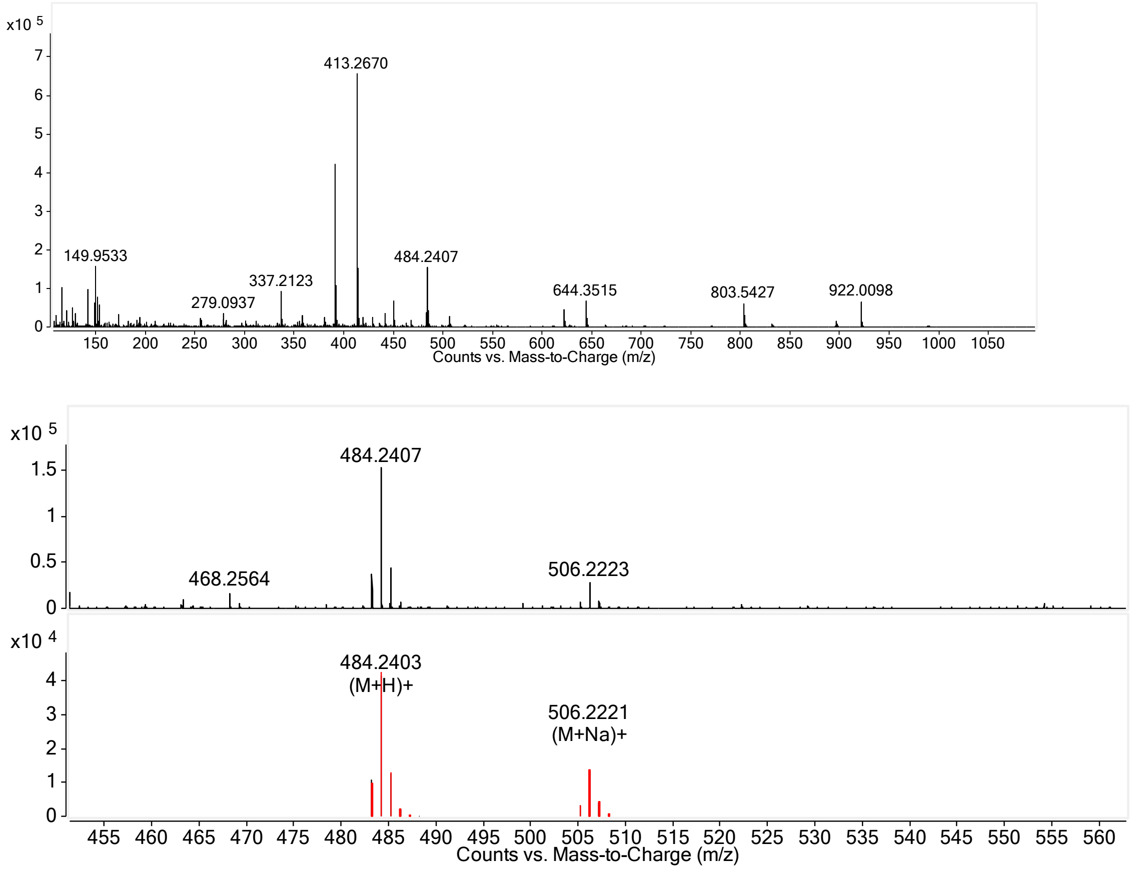

Figure S22. ESI-MS of Compound **3-BPin**

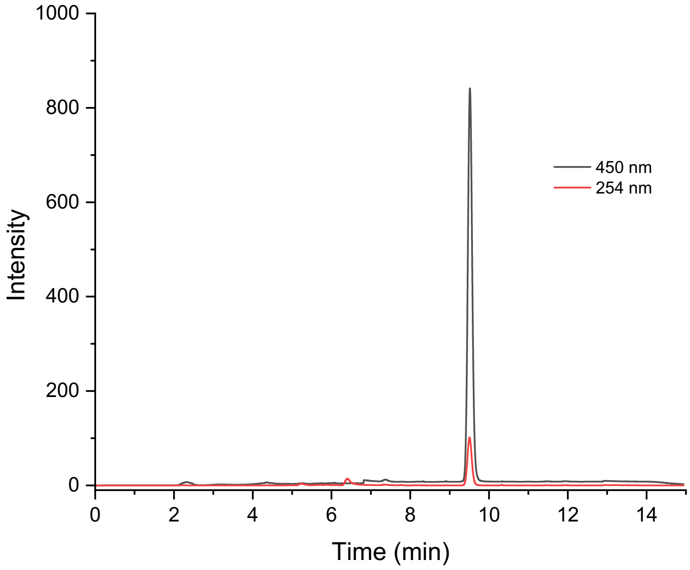

Figure S23. HPLC trace of Compound **3-BPin**

Compound III-BPin

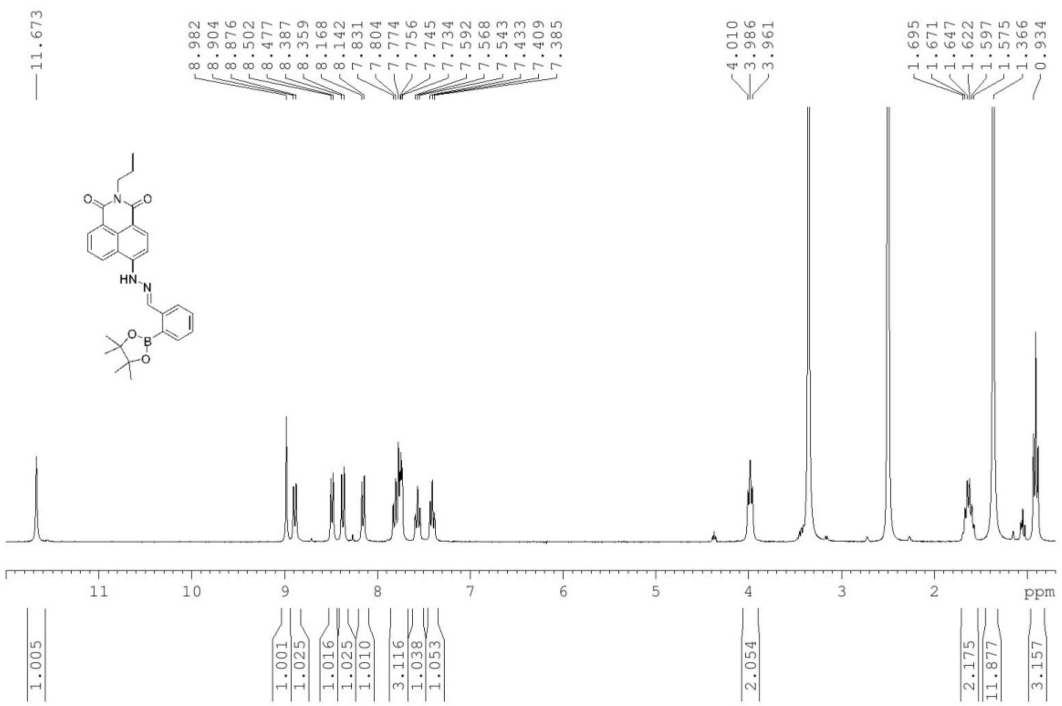

Figure S24. ^{1}H NMR (300 MHz, 25 ˚C, DMSO-d_6) of Compound **III-BPin**

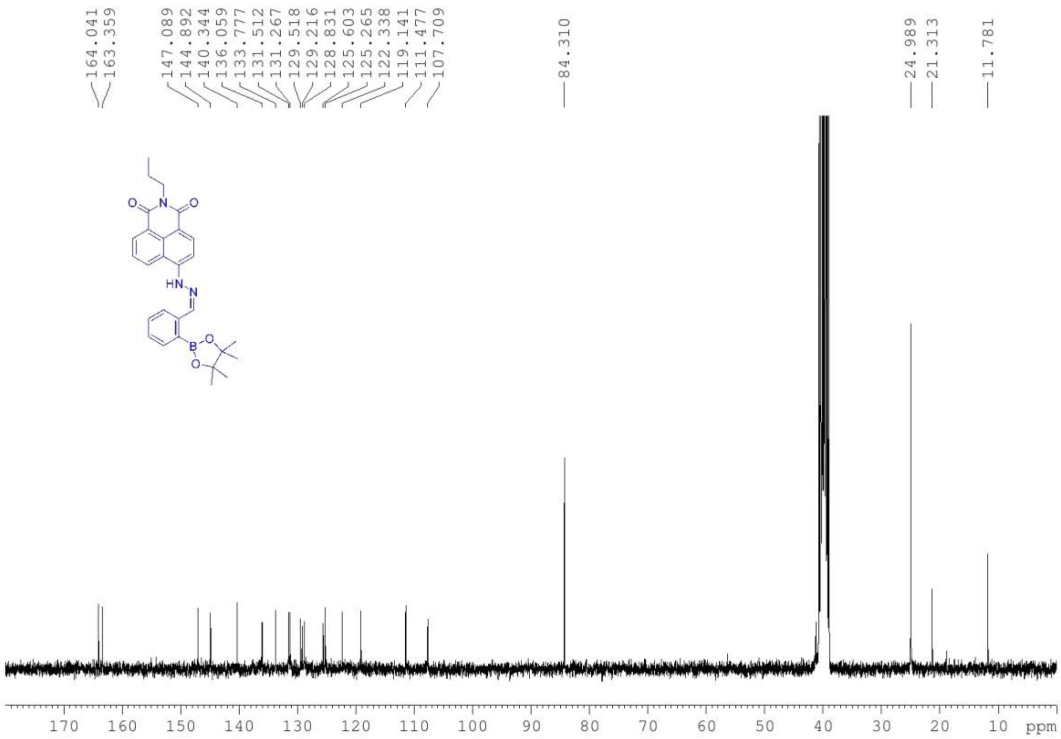

Figure S25. ^{1}H NMR (75.5 MHz, 25 ˚C, DMSO-d_6) of Compound **III-BPin**

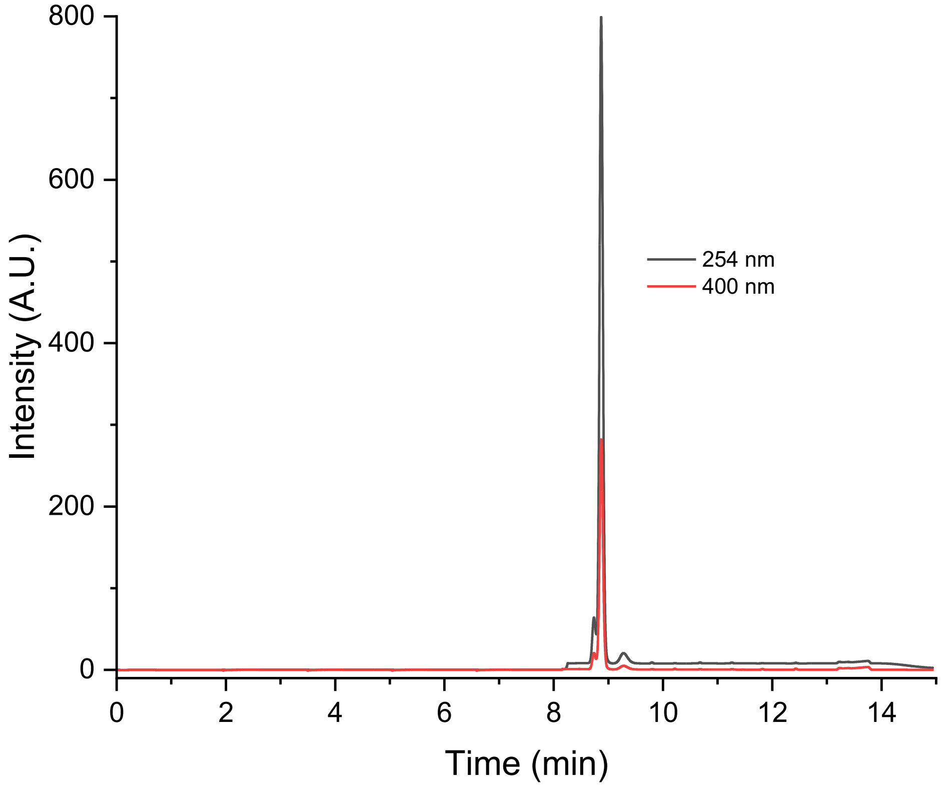

Figure S26. HPLC trace of Compound **III-BPin**

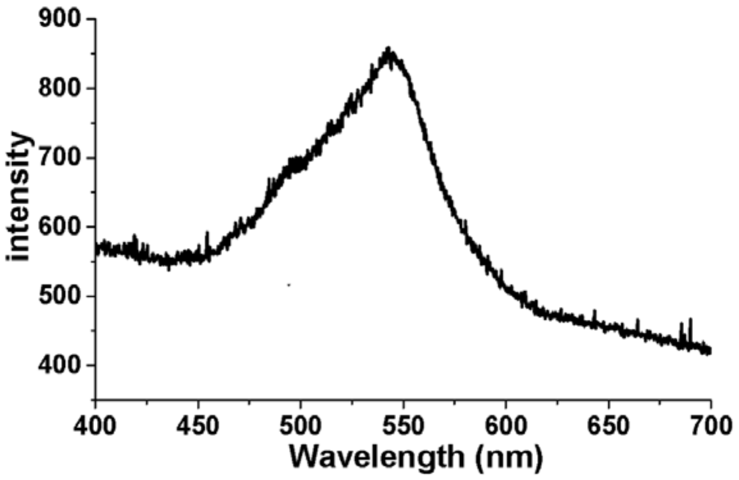

Figure S27: Two-photon fluorescence spectroscopy of compound **1** (10 mM) in DMSO at λ_{ex} = 810 nm.

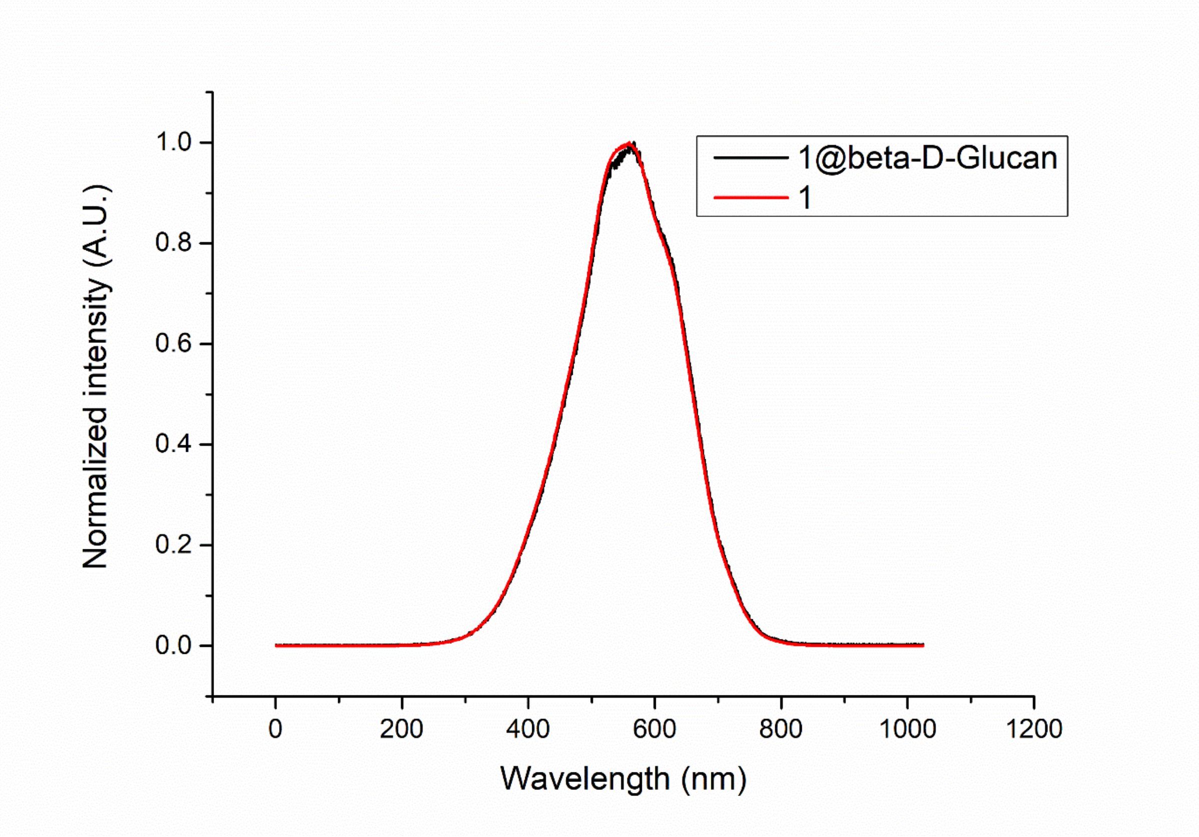

Figure S28: Normalised two-photon fluorescence spectroscopy of compound **1-BPin** (10 mM) in DMSO and corresponding **1@beta-D-glucan** hybrid (λ_{ex} = 910 nm) in 1:1 DMSO:PB Buffer.

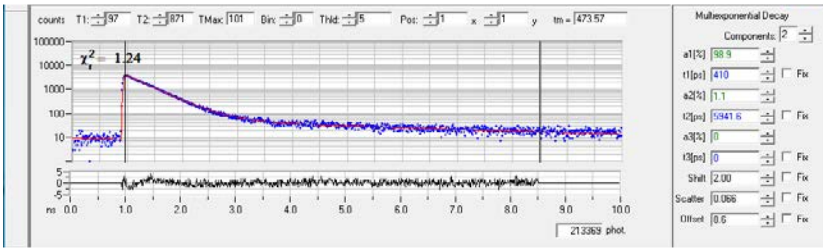

(a)

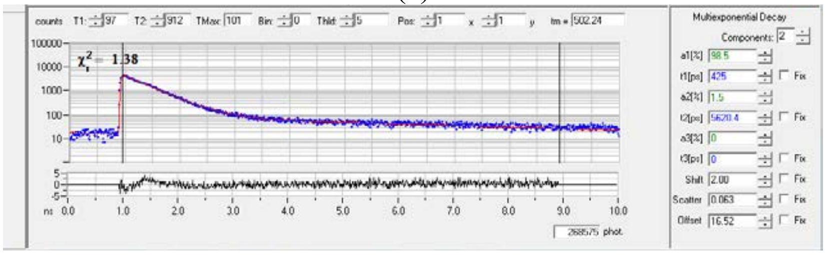

(b)

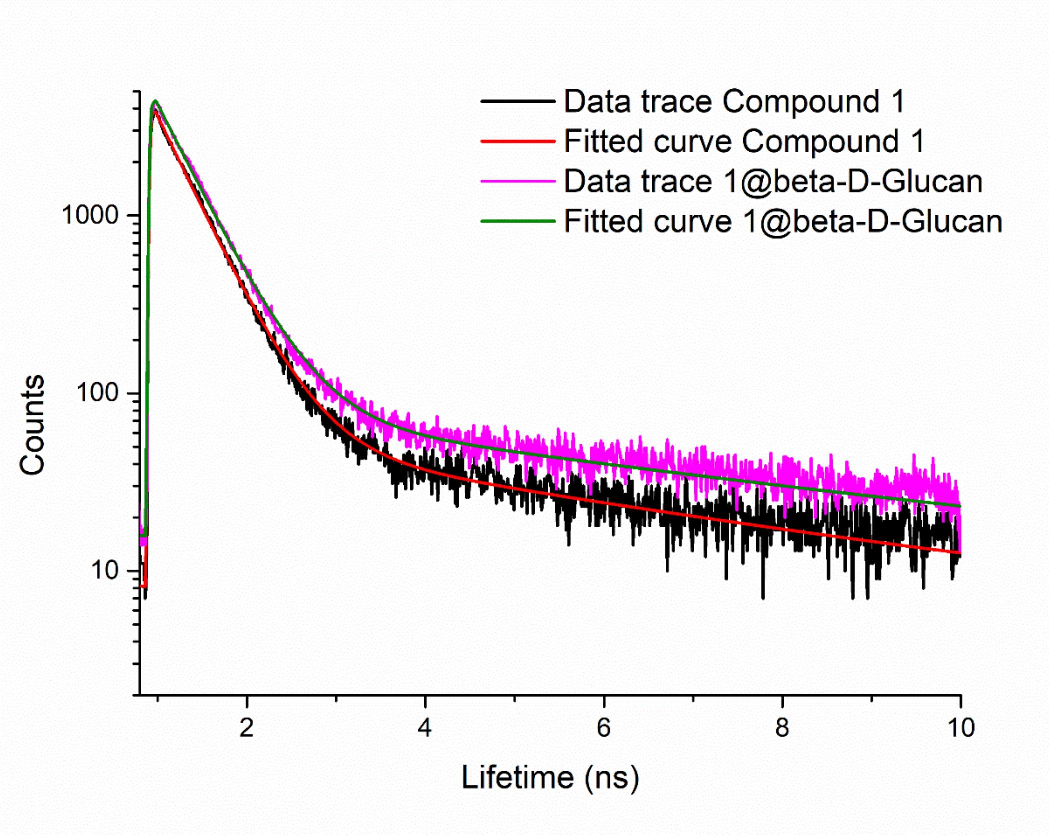

(c)

Figure S29: Fitted data for the two-photon fluorescence TCSPC spectroscopy of compound (a) Solution lifetime decay plot and data fit for **1-BPin** (10 mM) in DMSO**;** (b) Solution lifetime decay plot and data fit for **compound 1@beta-D-glucan** (in 1:1 DMSO:PB Buffer) at λ_{ex} = 910 nm; (c) overlay of the point decay data from (a) and (b).

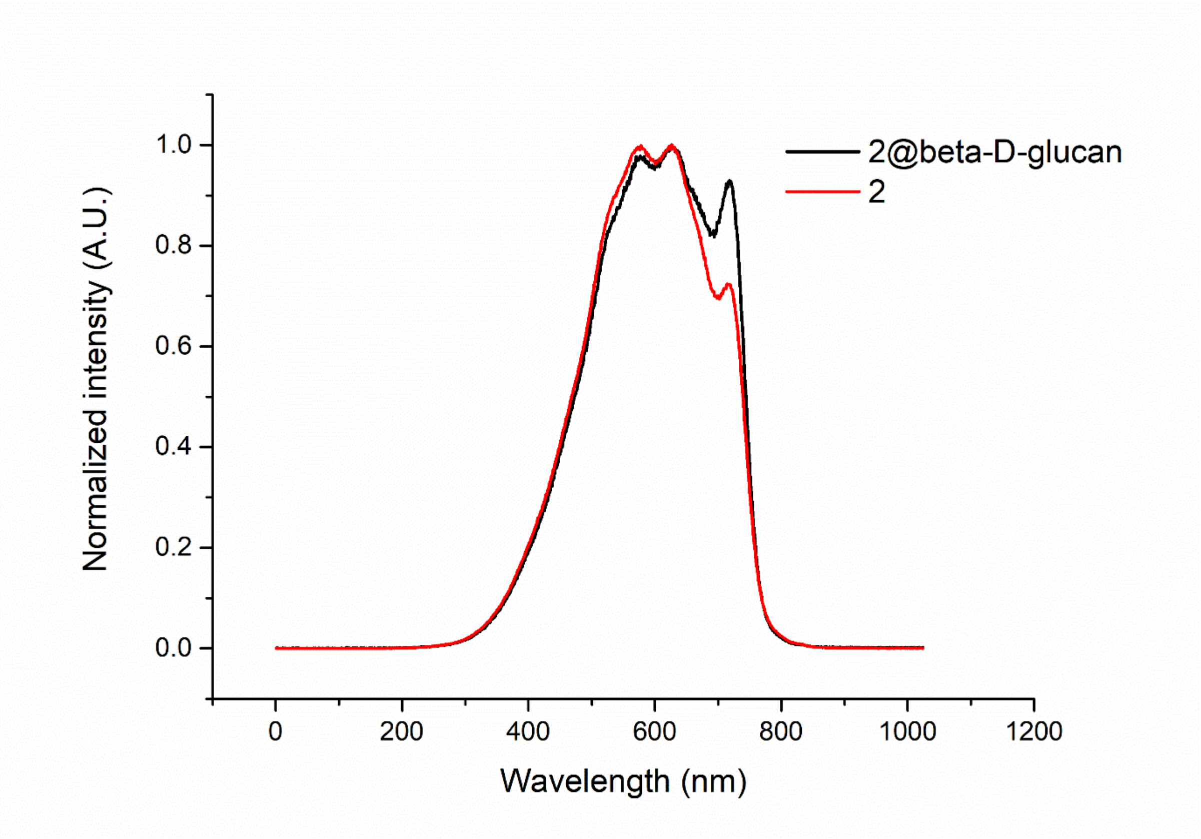

Figure S30: Normalised two-photon fluorescence spectroscopy of compound **2-BPin** (10 mM) in DMSO and corresponding **2@beta-D-glucan** hybrid (λ_{ex} = 910 nm) in 1:1 DMSO:PB Buffer.

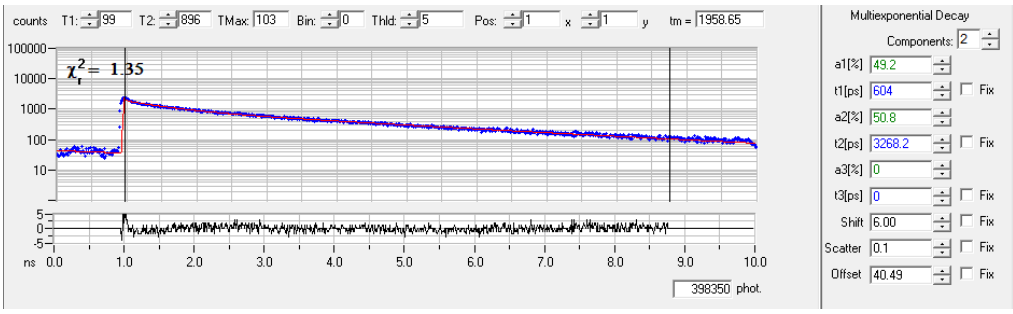

(a)

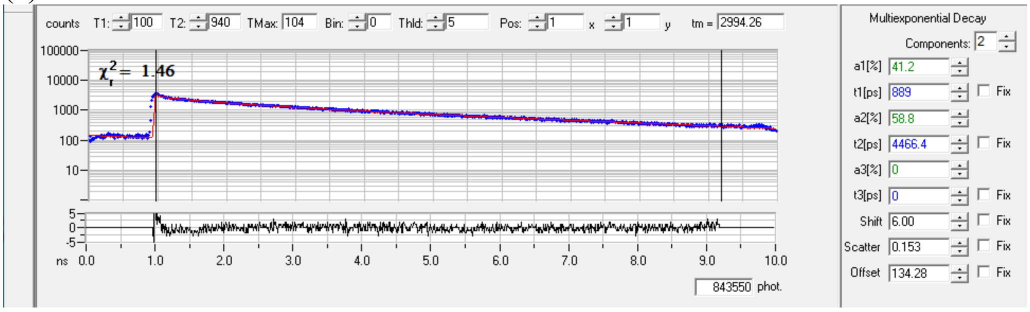

(b)

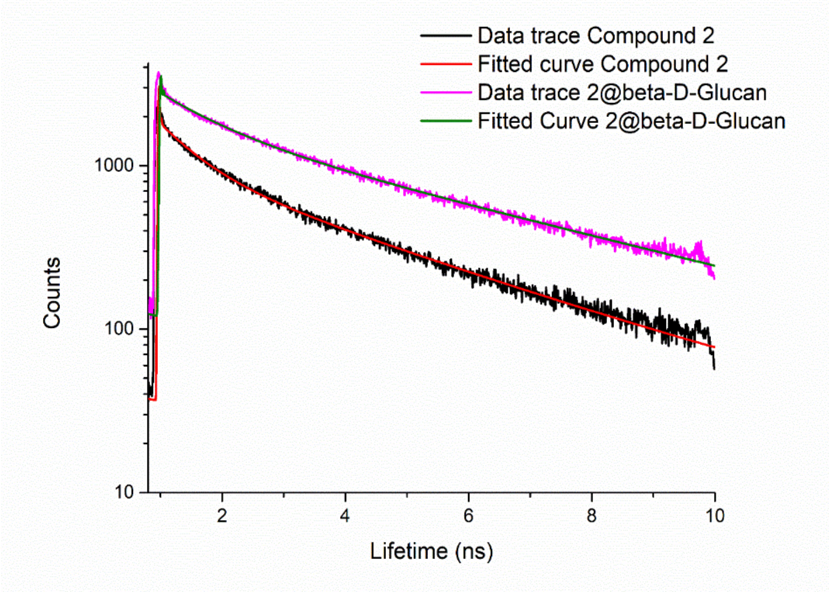

(c)

Figure S31: Figure S29: Fitted data for the two-photon fluorescence TCSPC spectroscopy of compound (a) Solution lifetime decay plot and data fit for **2-BPin** (10 mM) in DMSO**;** (b) Solution lifetime decay plot and data fit for **compound 2@beta-D-glucan** (in 1:1 DMSO:PB Buffer) at λ_{ex} = 910 nm; (c) overlay of the point decay data from (a) and (b).

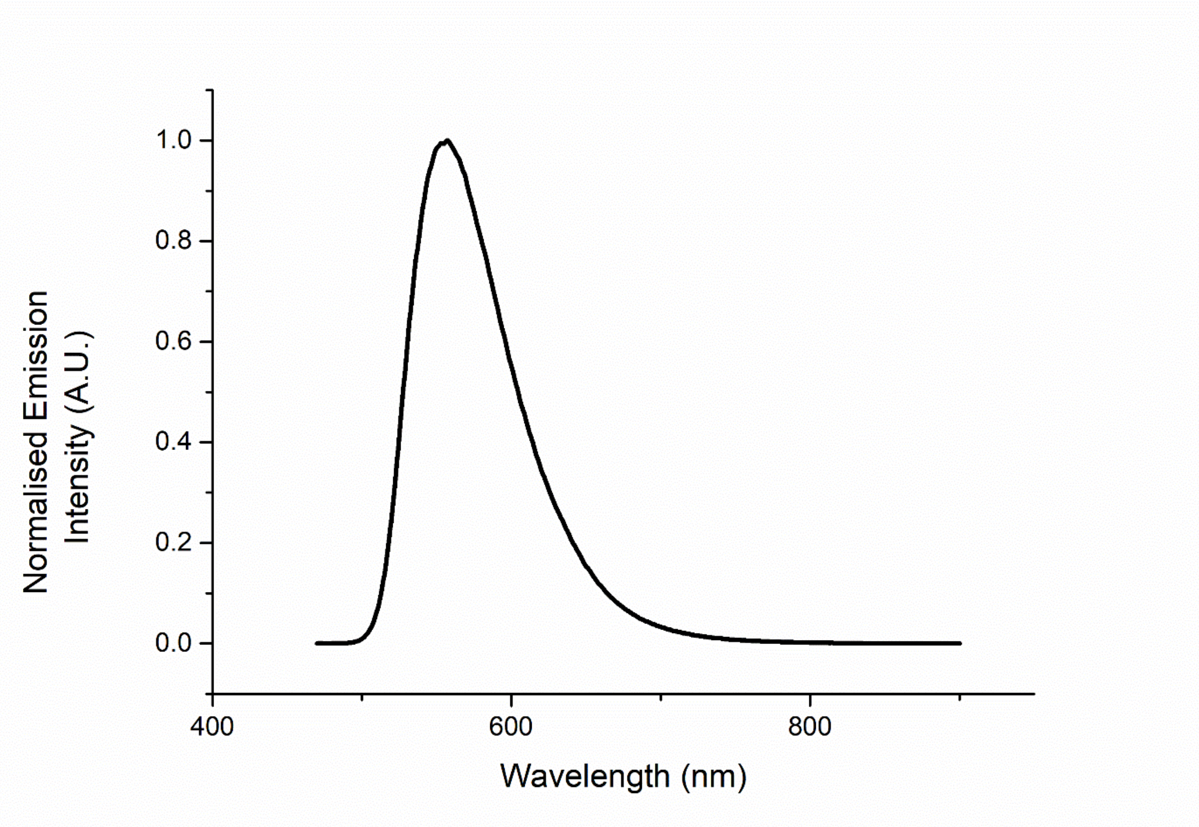

Figure S32: Single photon fluorescence emission of compound **III-BPin** (10 mM) in DMSO at λ_{ex} = 455 nm.

Table S1. Spectroscopic characteristics for compounds 1, 2-BPin and 3-BPin. Quantum yield was determined using the equation below in reference to fluorescein in 0.1M NaOH using the maximum excitation of each compound.

$$\phi_F = \phi_{ref} \cdot \frac{\eta^2{}_{sample}}{\eta^2{}_{sample}} \cdot \frac{E_{sample}}{A_{sample}} \cdot \frac{A_{ref}}{E_{ref}}$$

**Compound**	**λ_{ex} max (nm)**	**λ_{em} max (nm)**	**Φ_F (DMSO)**
1	440	524	0.231
2	427	522	0.153
3	463	540	0.489
III	466	540	0.334

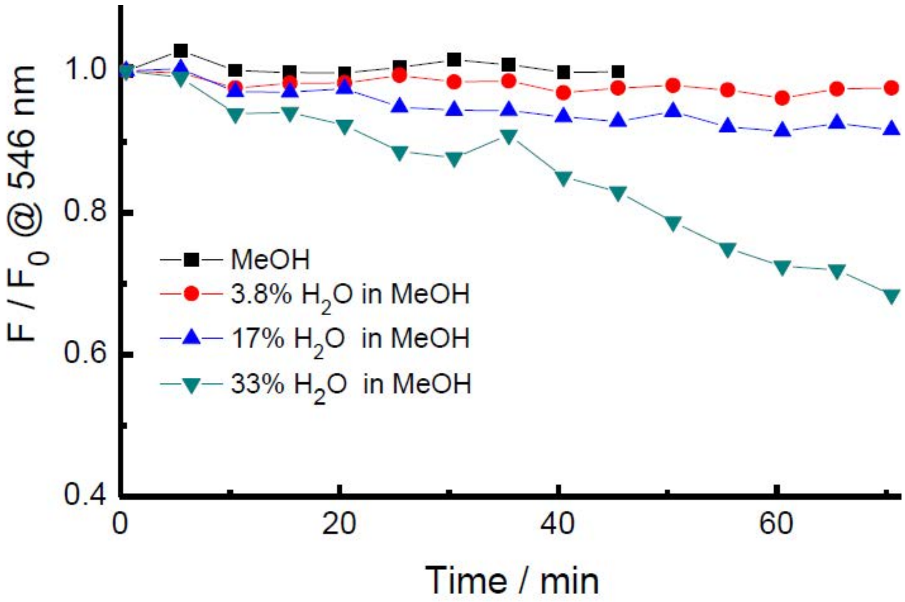

Figure S33. Evaluation of the kinetic stability of compound **III-BPin** in mixtures of solvents: Black (pure methanol), Red (3.8 % water in MeOH), Blue (17 % water in MeOH), green (33 % water in MeOH) Conc. [**III**] = 1 × 10^{-5} M; λex = 470 nm.

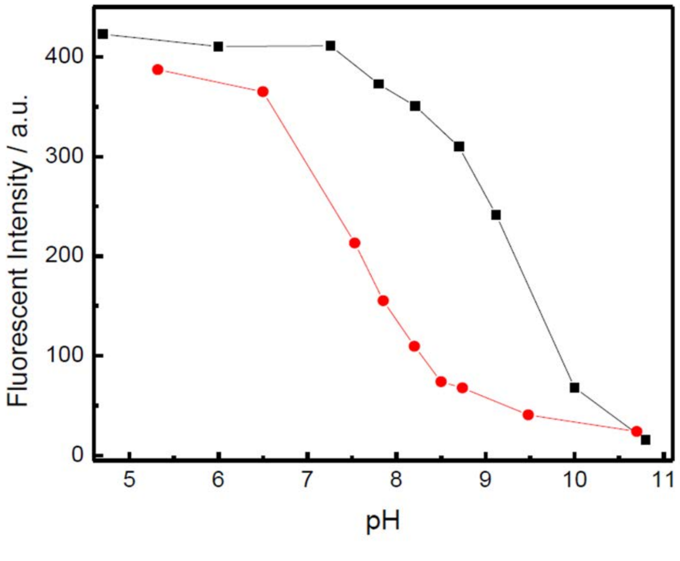

Figure S34: Fluorescence emission monitoring of the pH profiles characteristic of compound **III-BPin** behaviour in wet solvents, recorded with and without presence of D-fructose; [**III**] = 1 × 10^{-5} M; λex = 470 nm; Experiments were carried out in pH 7.4 PBS buffer solutions with 83 % *vol* content of methanol. To ensure full equilibration of the system, the solution was also left to stand for 30 minutes before being used for further experiments. Black line: compound **III-BPin** alone; Red Line: Compound **III:D-fructose** 1:1 mixture.

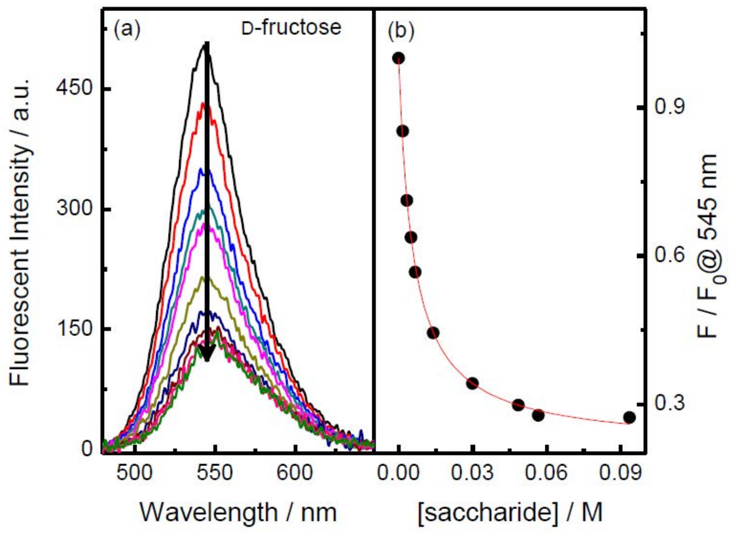

Figure S35: (a) Fluorescent spectral changes of compound **III-BPin** along with addition of D-fructose; (b) Ratio of F/ F0 at 545 nm of compound **III-BPin** *vs.* concentration of D-fructose; [**III**] = 1 × 10^{-5} M; λex = 470 nm; λem = 545 nm; experimented conducted in wet MeOH (3.8 % Milli-Q water).

Table S2. The observed binding stabilities and R2 of compound **III-BPin** with different saccharides

	K_{obs}	R^2
D-fructose	170.6 ± 7.52	0.997
D-galacotose	39.15 ± 2.33	0.998
D-mannose	19.04 ± 1.82	0.994
D-glucose	9.76 ± 2.79	0.981

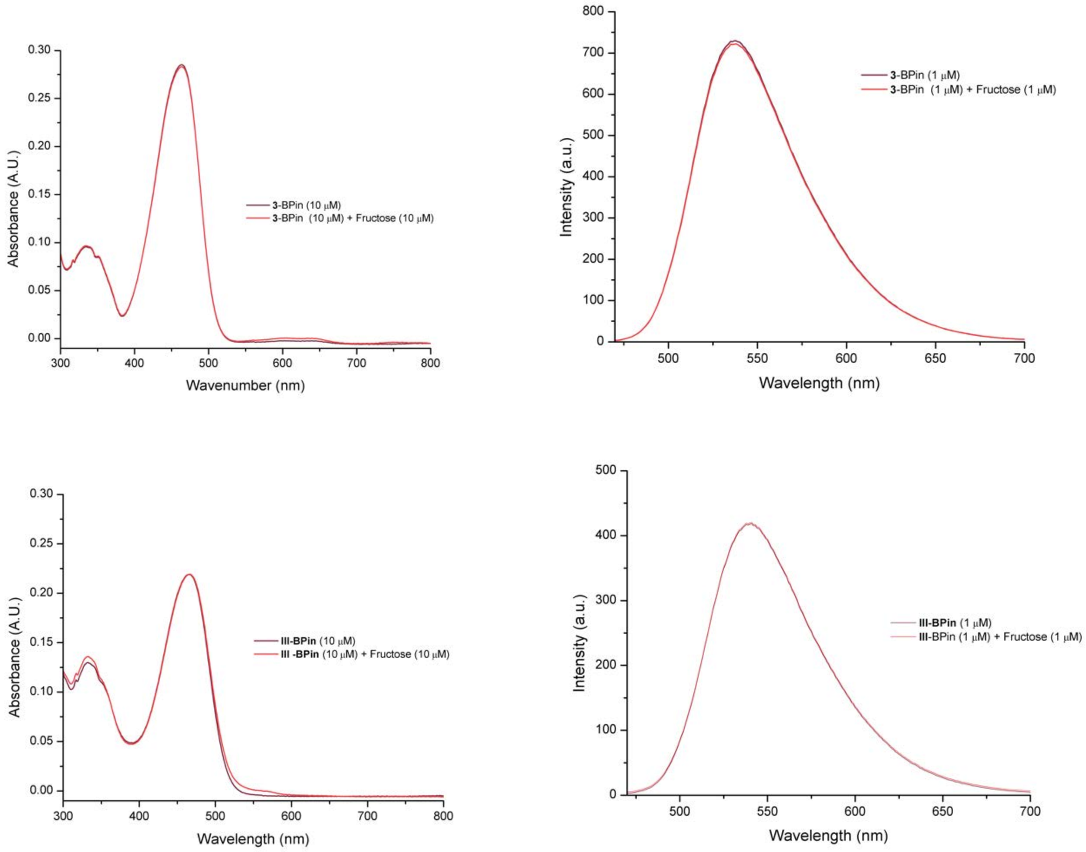

Figure S36. Representative fructose binding tests for compounds **3-BPin** and **III-BPin** in aqueous DMSO, monitored by UV-Vis and fluorescence spectroscopies (excitation at 405 nm). Stock solutions were made in DMSO and with D-fructose stock containing Milli-Q water (0.5% total end volume: this has prevented any crashing out of fructose from pure DMSO).

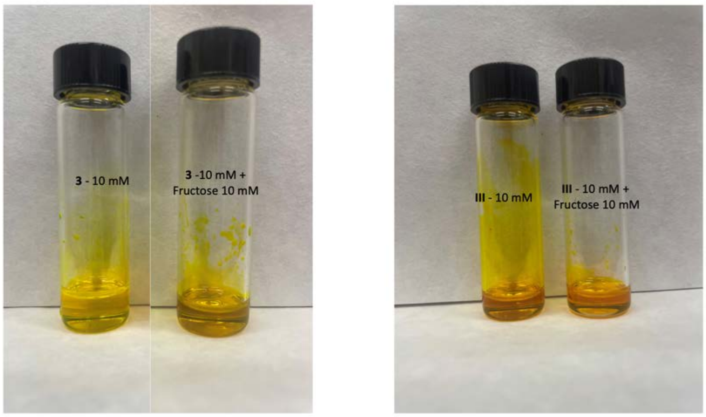

Figure S37. A visual comparison between the compound **3-BPin** and **III-BPin** with respect to their colorimetric response to addition of fructose. Stock solutions were made in DMSO and with D-fructose stock containing Milli-Q water to aid dissolving (0.5% total end volume: this has prevented any crashing out of fructose from pure DMSO).

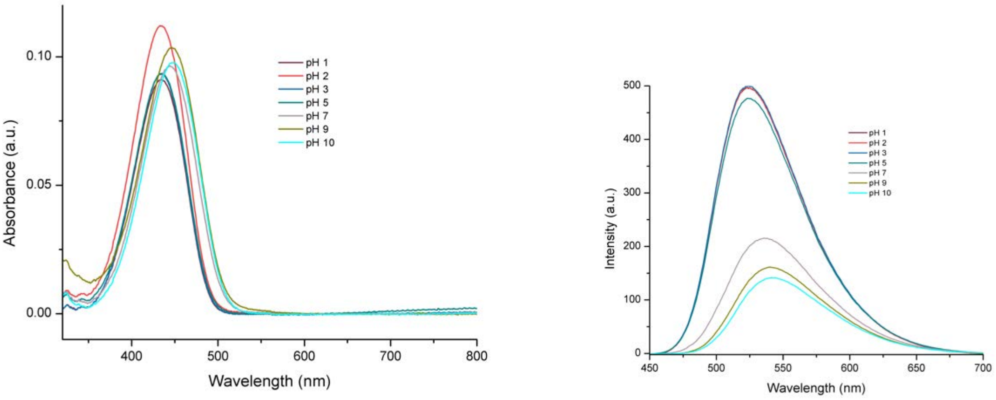

Figure S38. Spectroscopic investigations into the behaviour of compound **1** under variable pH conditions. Compound **1** final concentrations were 2.5 uM for fluorescence spectroscopy, 7.5 uM in DMSO for UV-Vis spectroscopy (1% DMSO in buffers). Conditions: Buffers from pH 1.1 to pH 10 were made using a Fisher brand Hydrus 600 pH meter in order to investigate the stability of compounds by fluorescence spectroscopy. The following buffer systems were used: pH=1.1 KCl/HCl; pH=2.0 KCl/HCl; pH=3.0 Glycine / HCl; pH = 5.0 Acetic Acid / Sodium acetate; pH = 7.0 Sodium phosphate Monobasic / Sodium phosphate Dibasic; pH = 9.0 Glycine/Sodium Hydroxide; pH= 10.0 Sodium Carbonate Anhydrous/Sodium Hydrogen Carbonate.

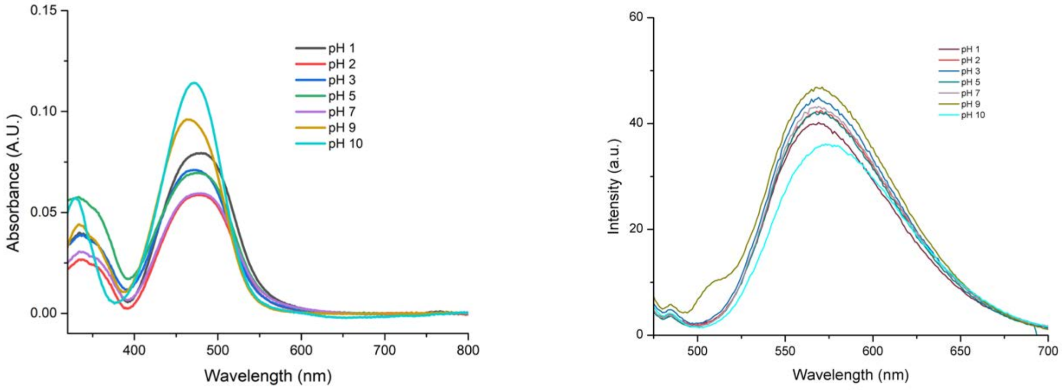

Figure S39. Spectroscopic investigations into the behaviour of compound **3-BPin** under variable pH conditions. Compound **1** concentration was 20 uM in DMSO, for fluorescence spectroscopy, 7.5 uM in DMSO for UV-Vis spectroscopy. Conditions: Buffers from pH 1.1 to pH 10 were made using a Fisher brand Hydrus 600 pH meter in order to investigate the stability of compounds by fluorescence spectroscopy. The following buffer systems were used: pH=1.1 KCl/HCl; pH=2.0 KCl/HCl; pH=3.0 Glycine / HCl; pH = 5.0 Acetic Acid / Sodium acetate; pH = 7.0 Sodium phosphate Monobasic / Sodium phosphate Dibasic; pH = 9.0 Glycine/Sodium Hydroxide; pH= 10.0 Sodium Carbonate Anhydrous/Sodium Hydrogen Carbonate.

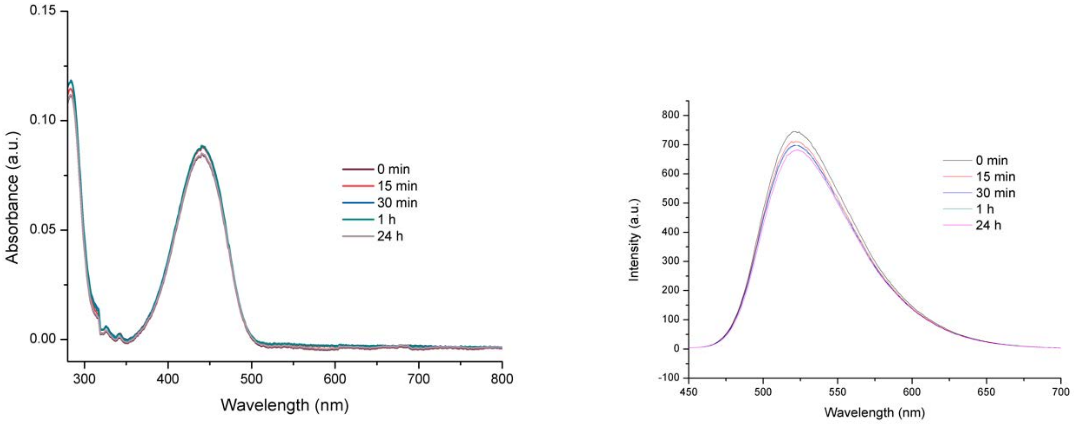

Figure S40. Spectroscopic investigations into the behaviour of compound **1** over 24 h in DMSO solutions. Compound 1 concentration was 7.5 uM in DMSO for UV-Vis spectroscopy, 2.5 uM in DMSO, for fluorescence spectroscopy,

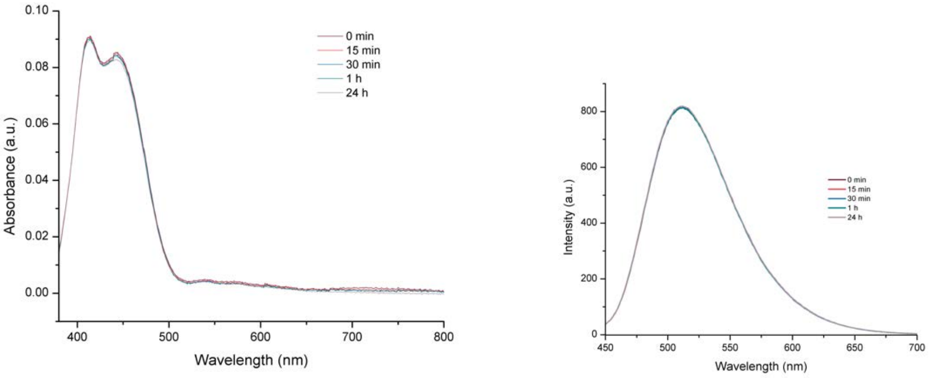

Figure S41. Spectroscopic investigations into the behaviour of compound **1** over 24 h in DMSO solutions. Compound 1 concentration was 7.5 uM in DMSO:RMPI cellular medium (1:1 v/v) for UV-Vis spectroscopy, 2.5 uM in DMSO:RMPI cellular medium (1:1 v/v), for fluorescence spectroscopy.

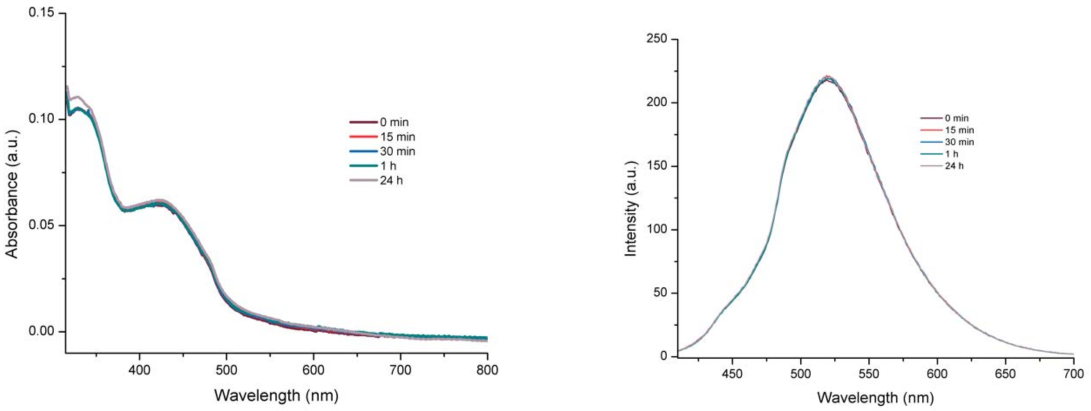

Figure S42. Spectroscopic investigations into the behaviour of compound **2-BPin** over 24 h in DMSO solutions. Compound 2-BPin concentration was 7.5 uM in DMSO for UV-Vis spectroscopy, 2.5 uM in DMSO, for fluorescence spectroscopy.

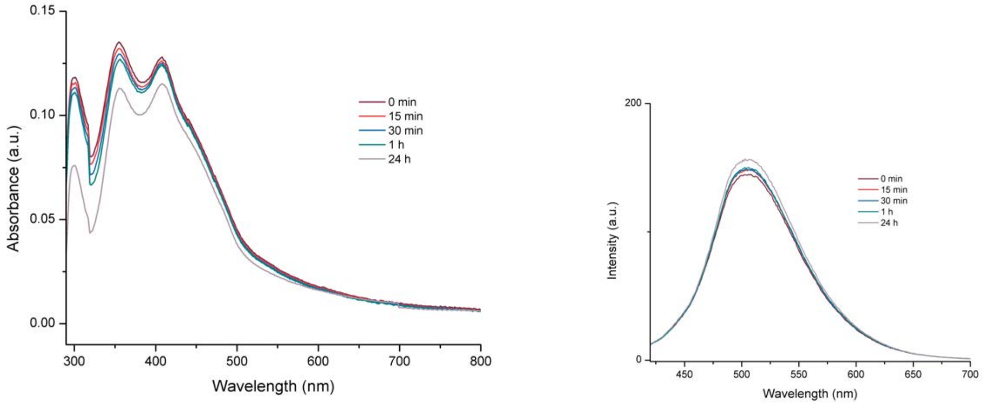

Figure S43. Spectroscopic investigations into the behaviour of compound **2-BPin** over 24 h in DMSO solutions. Compound 2-BPin concentration was 7.5 uM in DMSO:RMPI cellular medium (1:1 v/v) for UV-Vis spectroscopy, 2.5 uM in DMSO:RMPI cellular medium (1:1 v/v), for fluorescence spectroscopy.

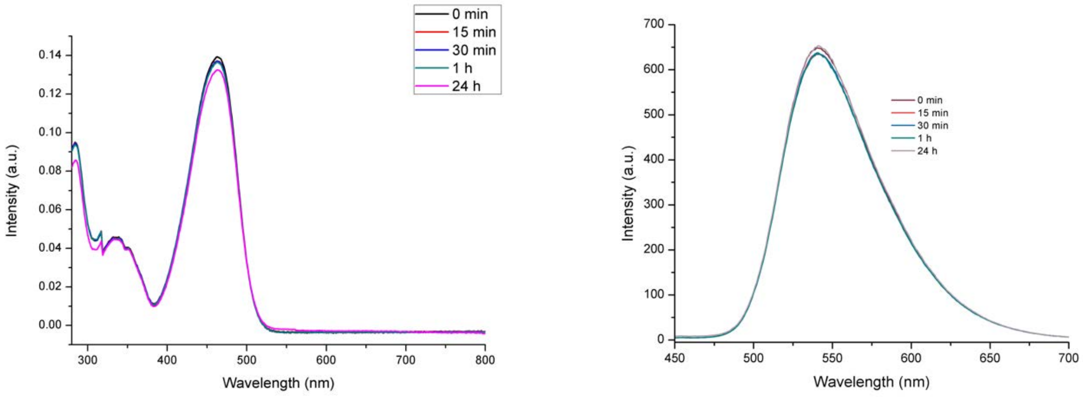

Figure S44. Spectroscopic investigations into the behaviour of compound **3-BPin** over 24 h in DMSO solutions. Compound 2-BPin concentration was 7.5 uM in DMSO for UV-Vis spectroscopy, 2.5 uM in DMSO, for fluorescence spectroscopy.

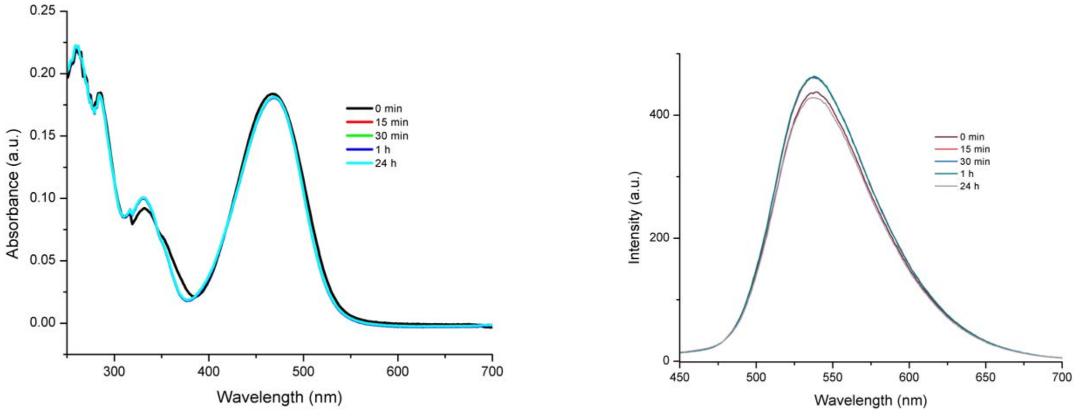

Figure S45. Spectroscopic investigations into the behaviour of compound **3-BPin** over 24 h in DMSO solutions. Compound 2-BPin concentration was 7.5 uM in DMSO:RMPI cellular medium (1:1 v/v) for UV-Vis spectroscopy, 2.5 uM in DMSO:RMPI cellular medium (1:1 v/v), for fluorescence spectroscopy.

4. Cellular Imaging Assays

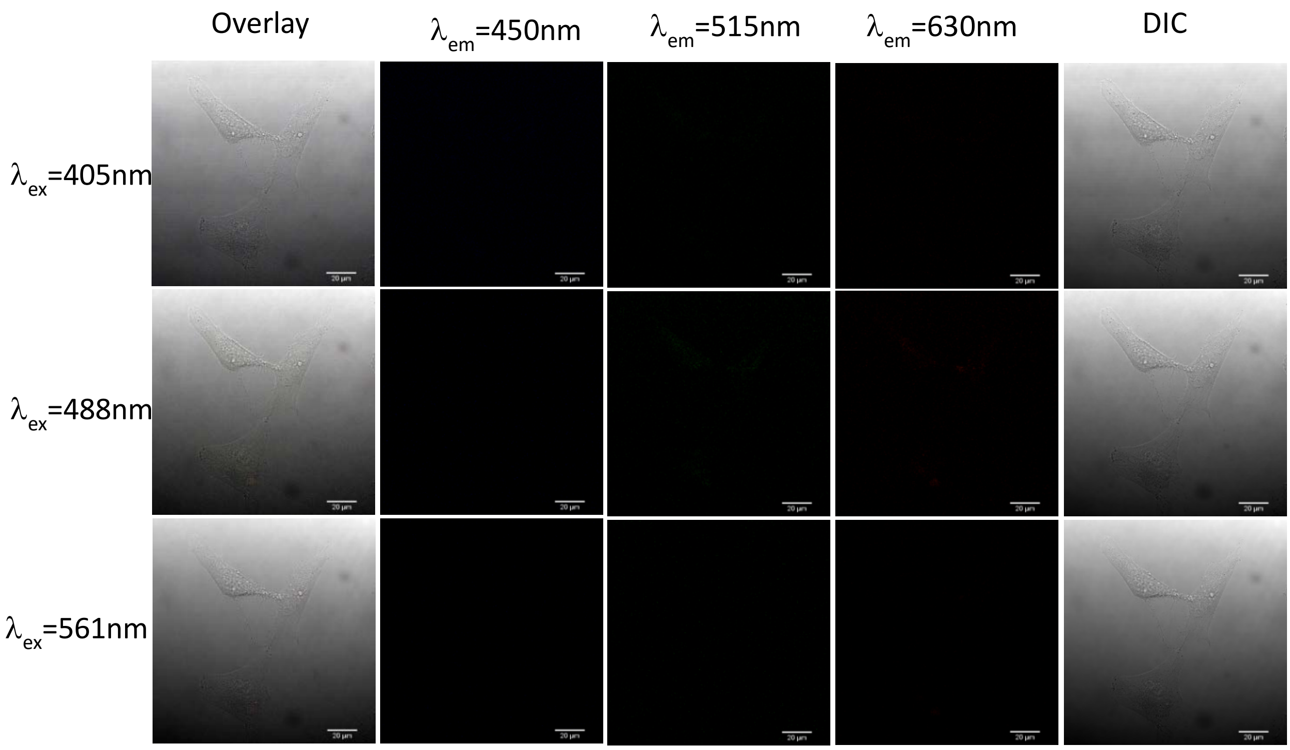

Figure S46. Control experiments: Confocal images of living HeLa cells incubated with 1% DMSO for 15 min at 37 °C

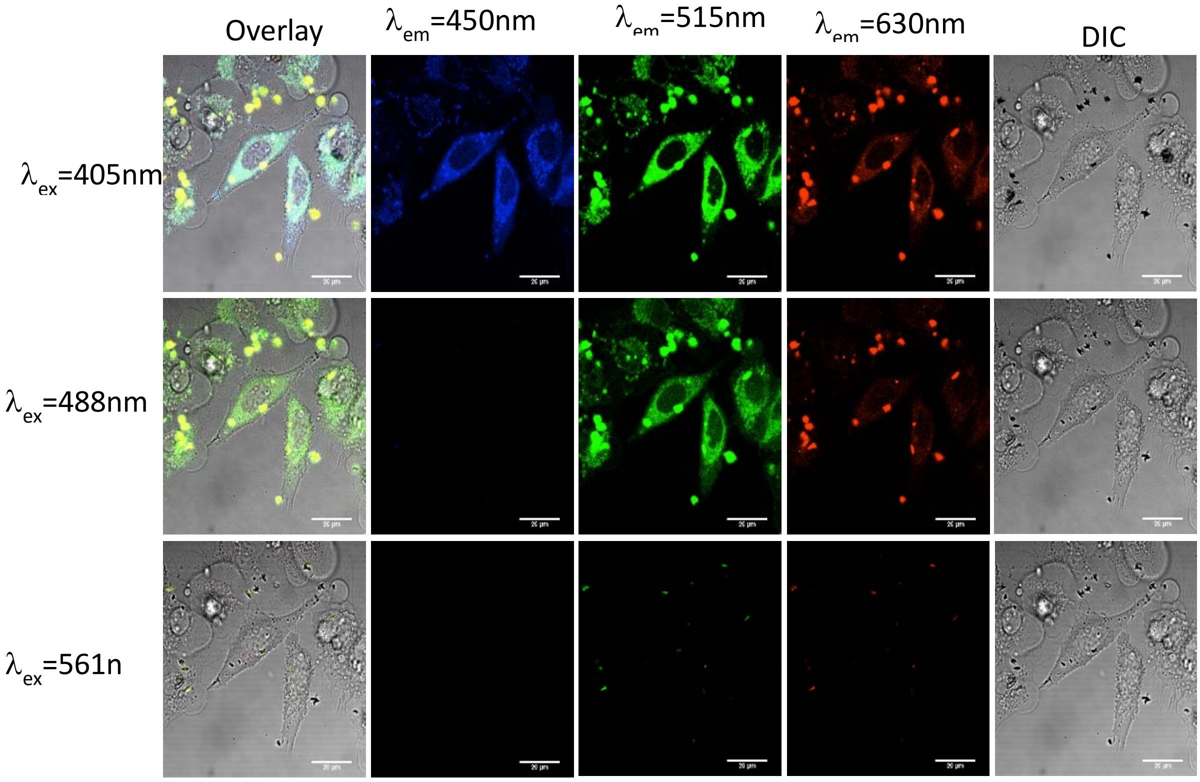

Figure S47. Laser confocal fluorescence imaging of Compound **1-BPin** at 37 °C (living HeLa cells micrographs show cellular membrane aggregation at 100 μM concentration in the absence of PBS).

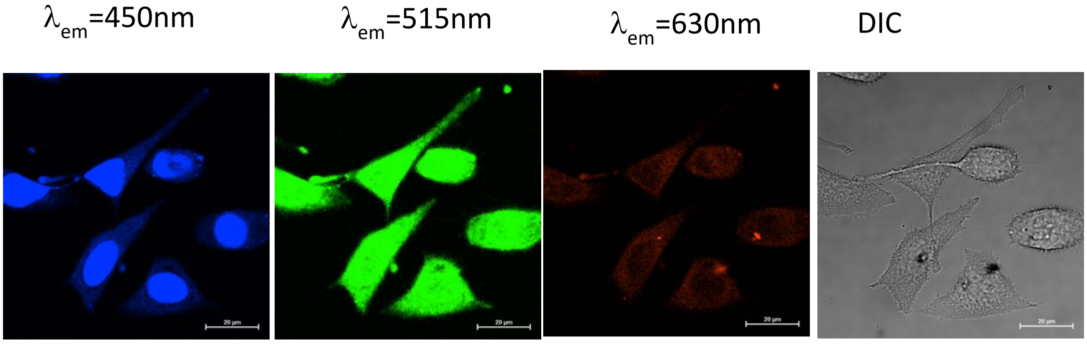

Figure S48. Laser confocal fluorescence imaging of Compound **1-BPin** at 37 °C co-incubated with Hoechst nuclear stain in living HeLa cells (micrographs recorded after PBS washing. Hoescht nucleic acid stain, 1 μg/mL, 30 min, λex = 405 nm, 1-BPin 100 μM, 15 min incubation.

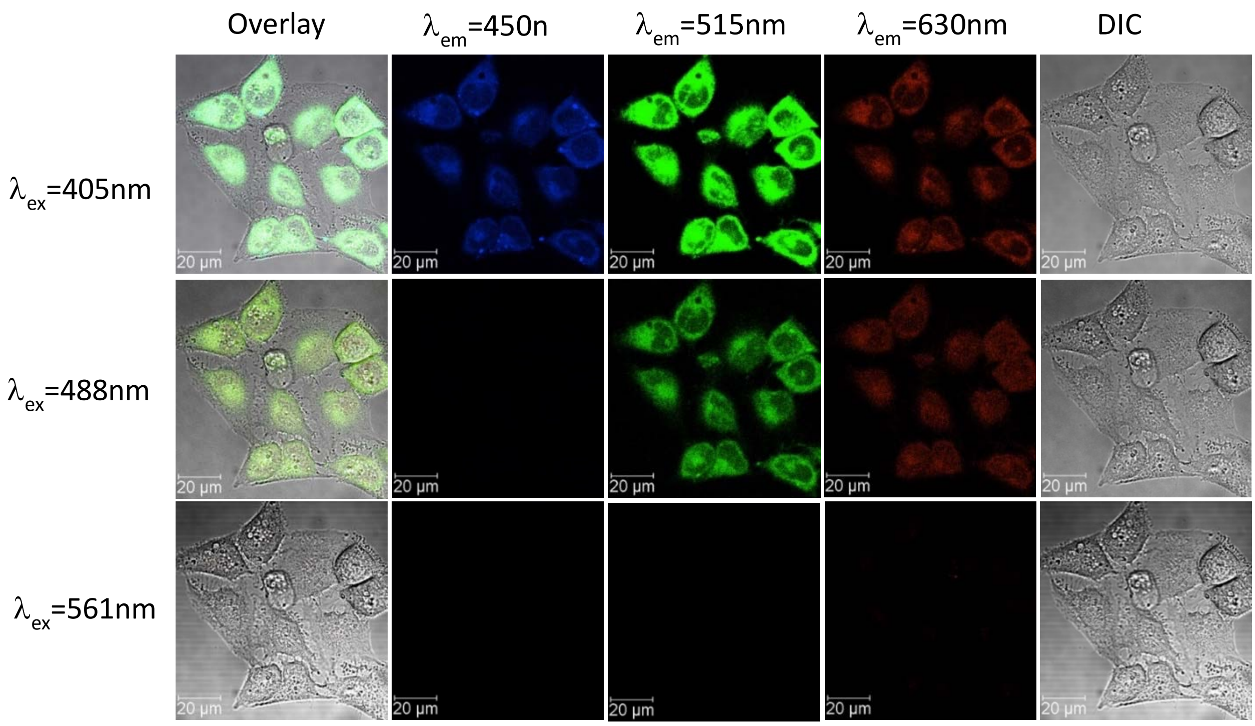

Figure S49. Laser confocal fluorescence imaging of Compound **1-BPin** at 4 ºC, 15 min incubation, 100 µM, 1 % DMSO in living HeLa cells (micrographs recorded after PBS washing).

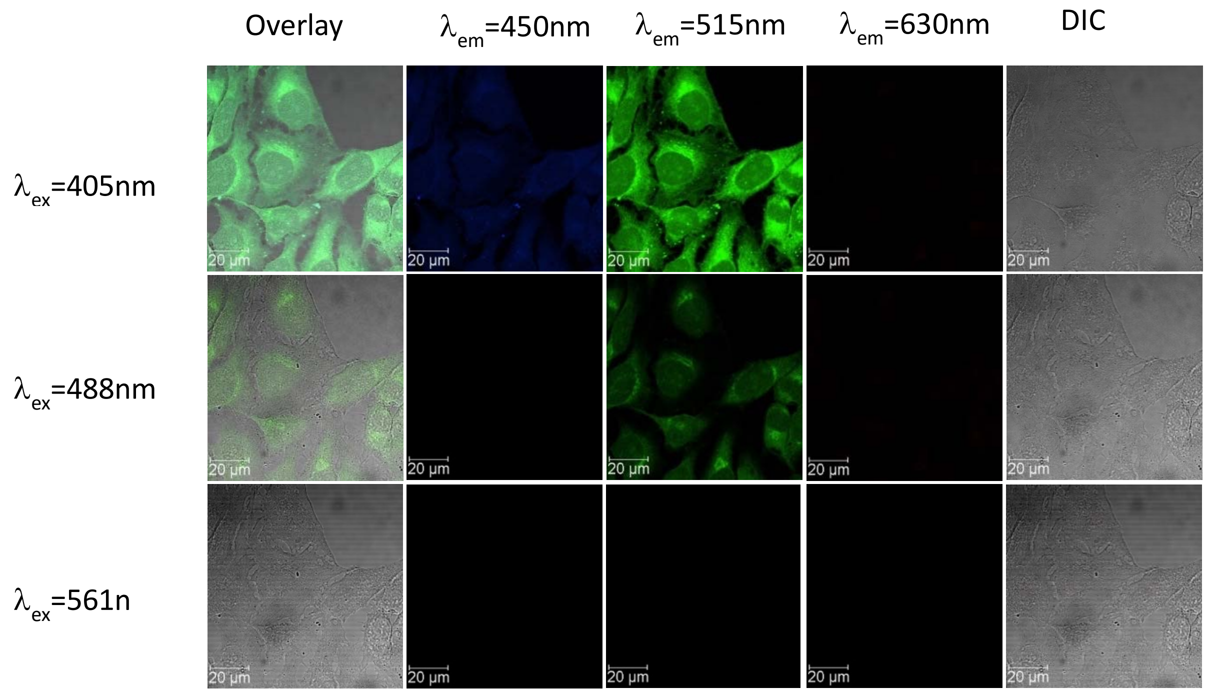

Figure S50. Laser confocal fluorescence imaging of **Compound 1@Beta-D-Glucan**, 37 ºC, 15 min incubation, 0.5 % DMSO in living HeLa cells.

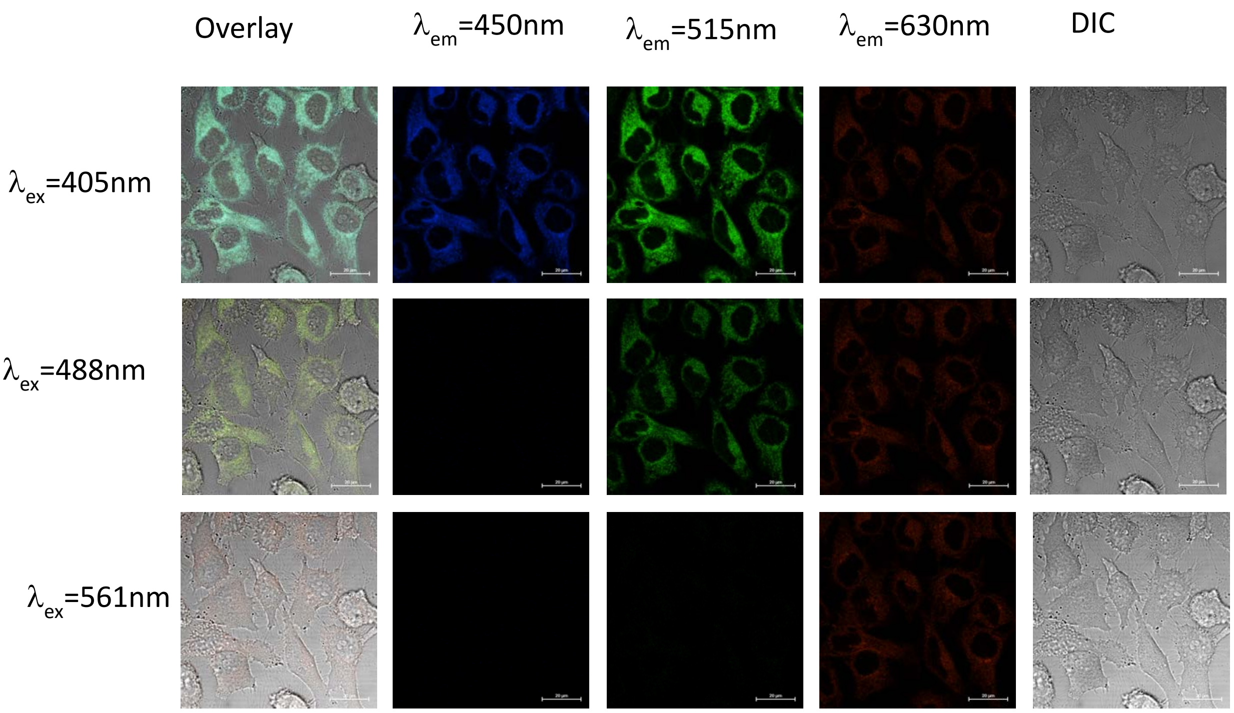

Figure S51. Laser confocal fluorescence imaging of **Compound 2-BPin** at 4 ºC, 15 min incubation, 100 uM, 1% DMSO in living HeLa cells.

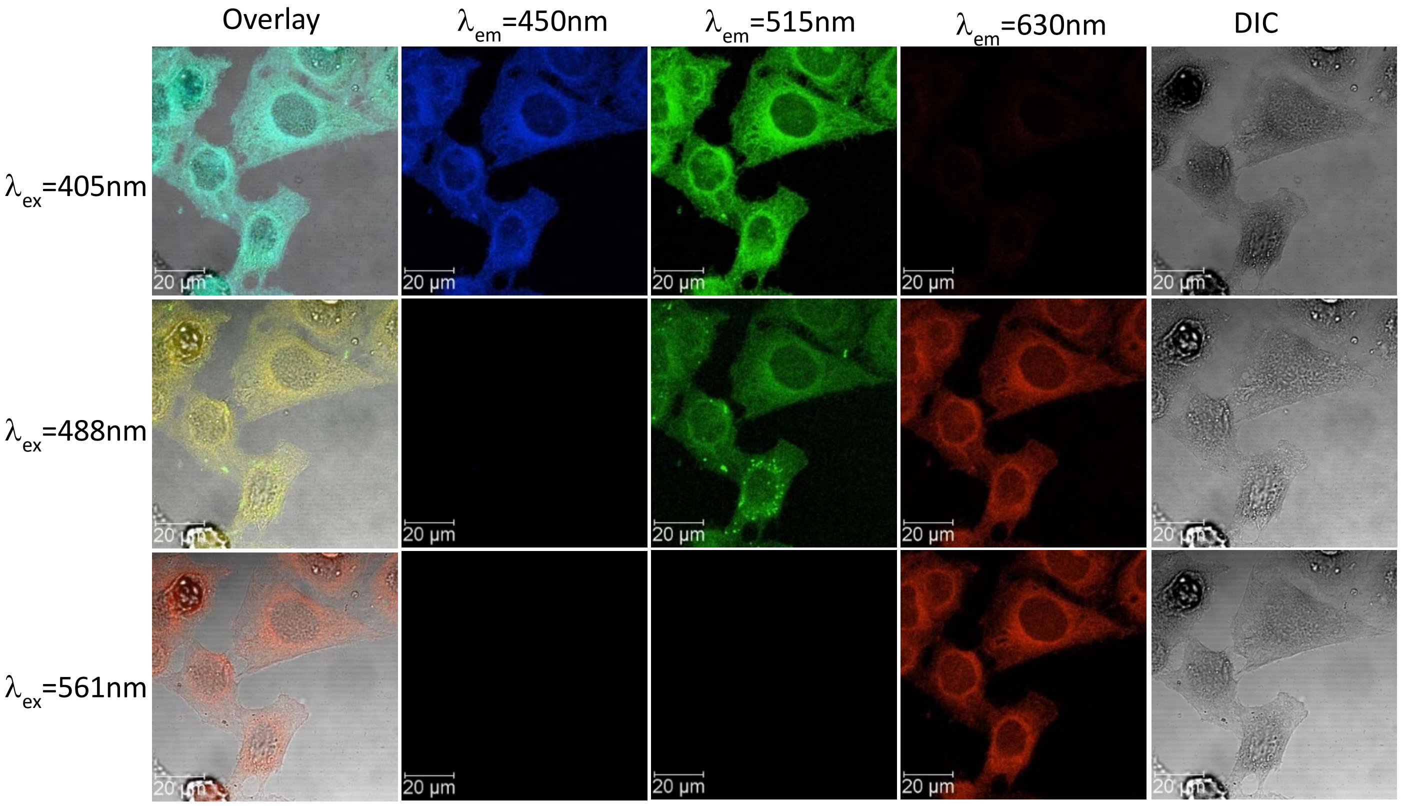

Figure S52. Confocal images recorded for living HeLa cells incubated with **Compound 2-BPin** at 100 μM for 15 min under various laser excitation conditions (1% DMSO, at 37 ºC);

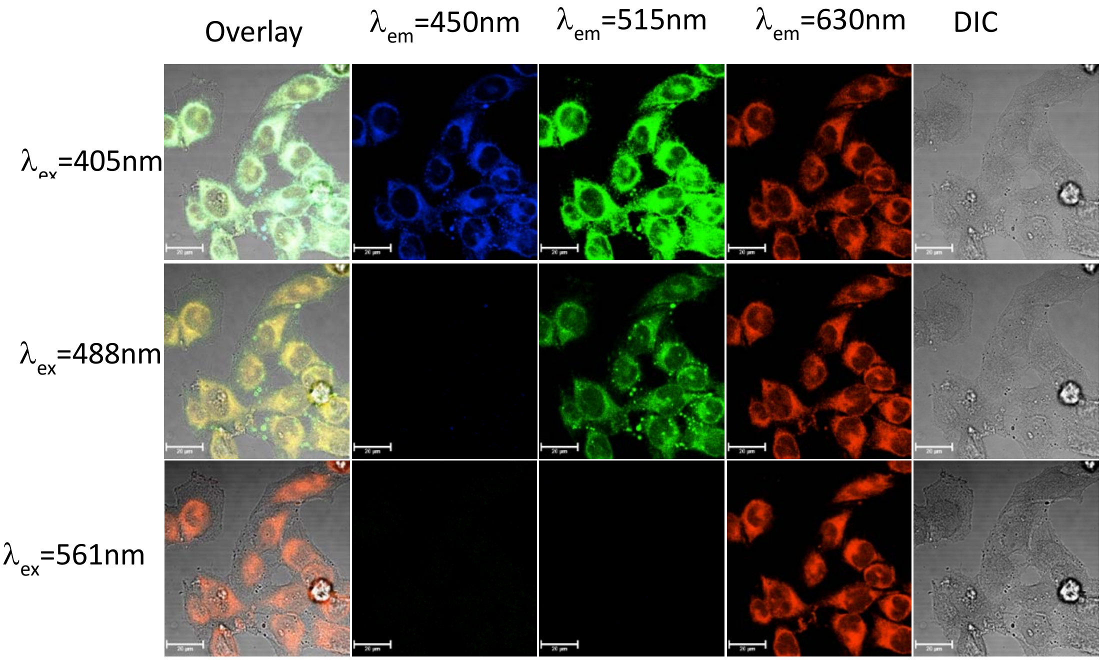

Figure S53. Confocal images recorded for living HeLa cells incubated with compound 2-BPin at 100 µM for 15 min = (1% DMSO, at 37 ºC); co-localised with ER Tracker dye.

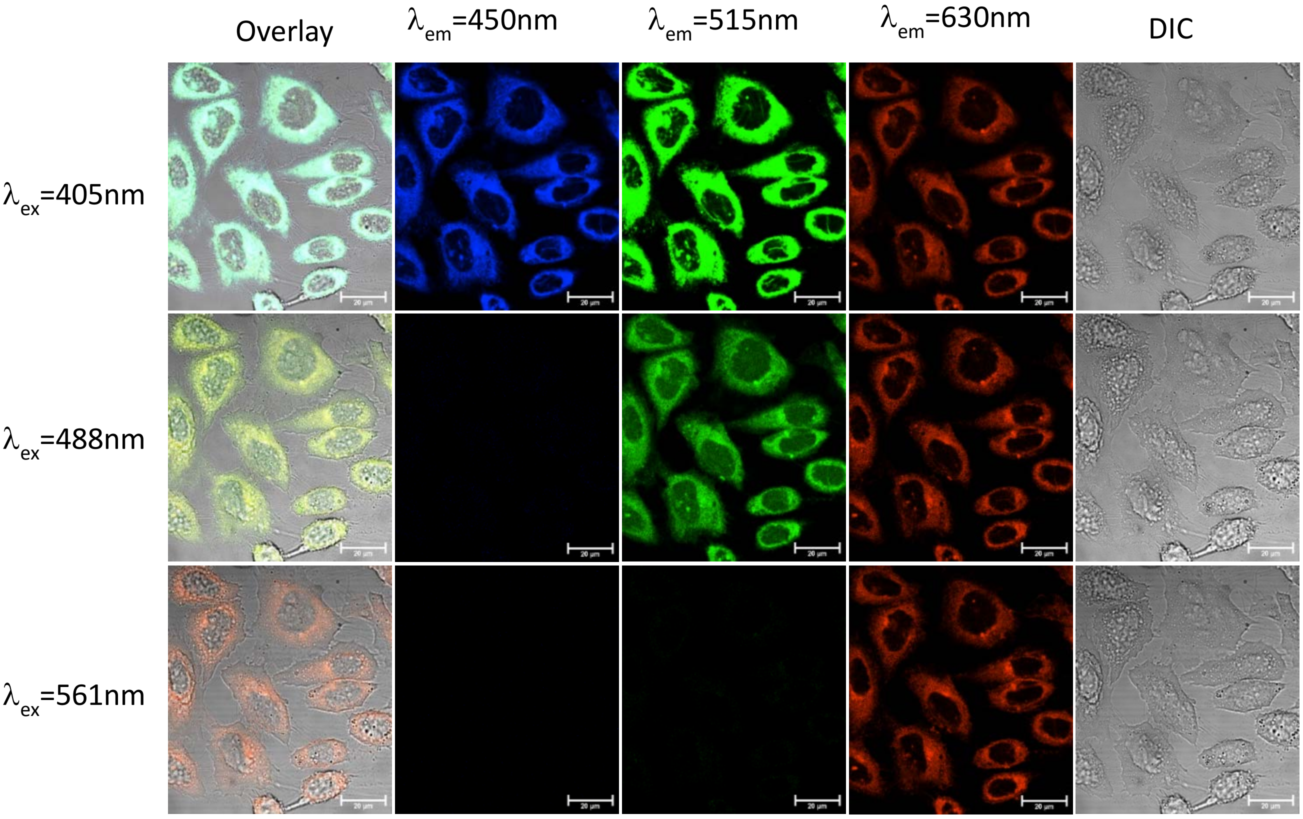

Figure S54. Laser confocal fluorescence imaging of **Compound 2@Beta-D-Glucan** at 37 ºC, 15 min incubation, 0.5 % DMSO in living HeLa cells.

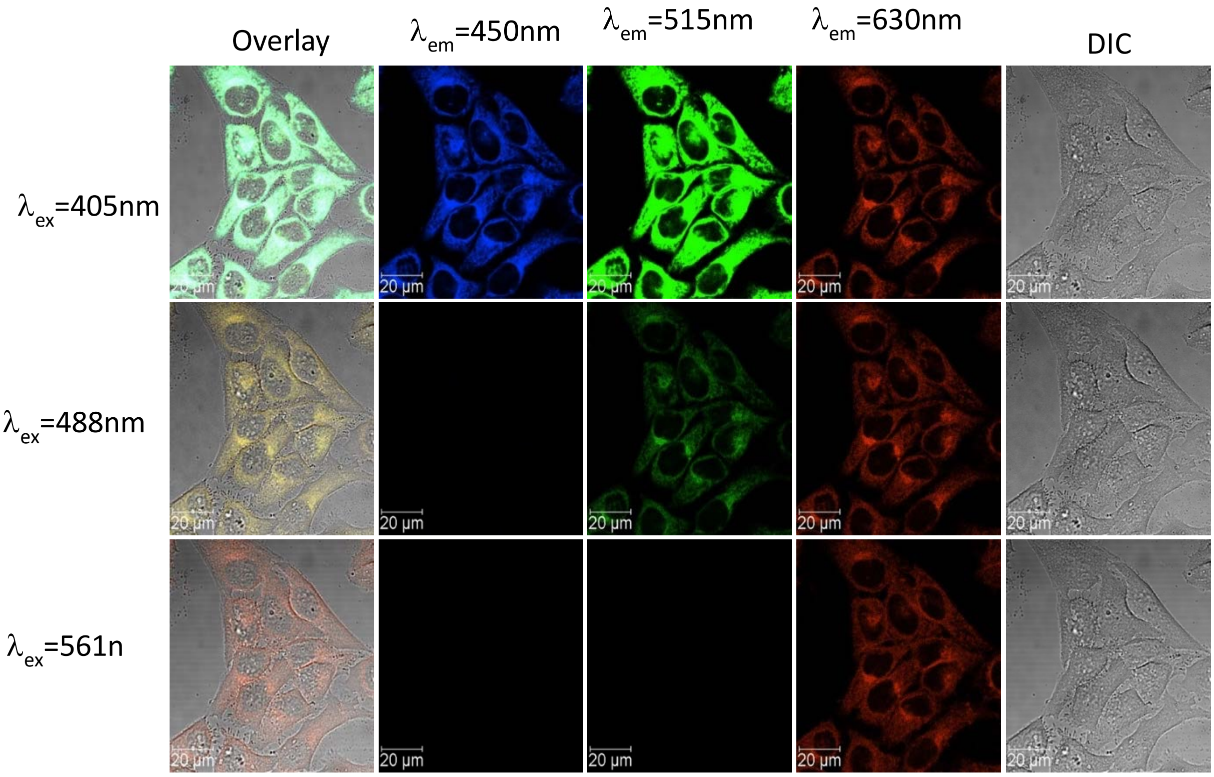

Figure S55. Laser confocal fluorescence imaging of Compound **2-BPin** at 4 ^{o}C, 15 min incubation, 1 % DMSO in living HeLa cells.

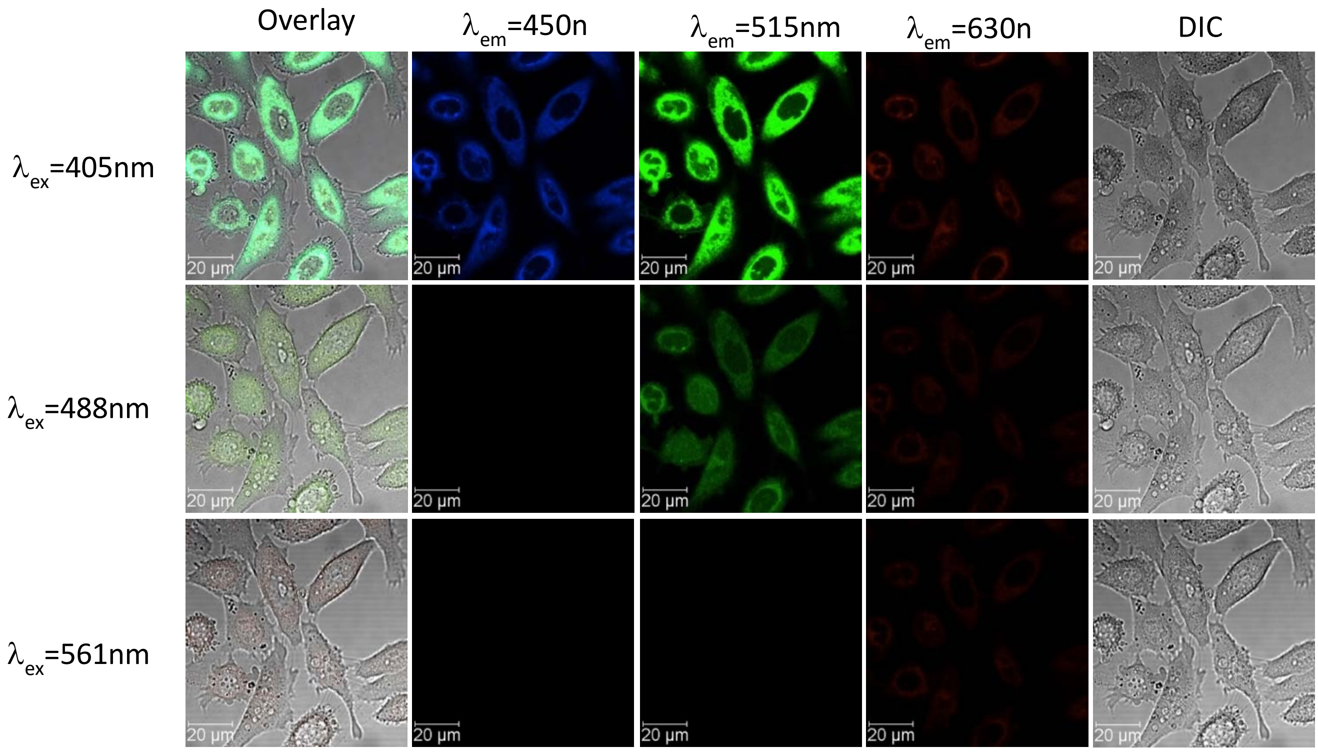

Figure S56. Laser confocal fluorescence imaging of **Compound 2@Beta-D-Glucan** at 37°C, 15 min incubation, 2 % DMSO in living HeLa cells.

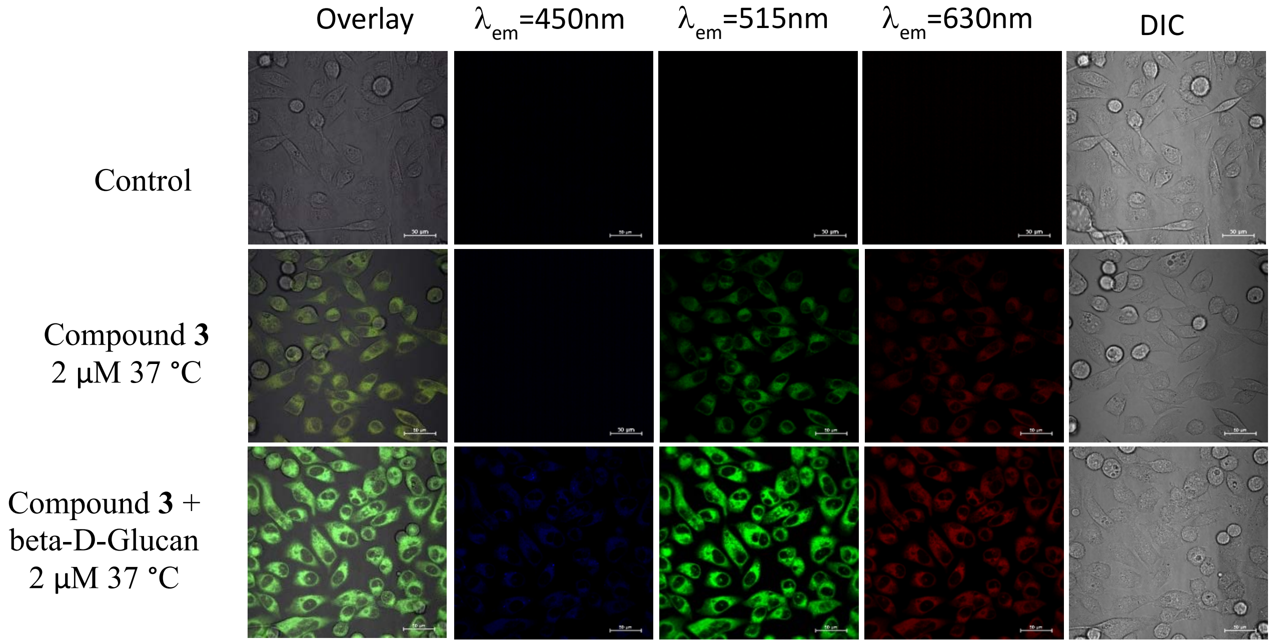

Figure S57. Confocal Fluorescence imaging with compound **3-BPin** in living PC-3 cells, 15 min incubation under various conditions (λex. 405 nm)

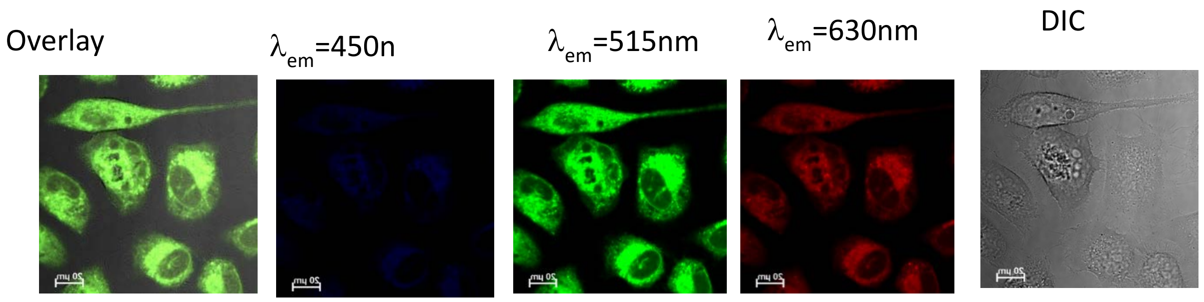

Figure S58. Confocal Fluorescence imaging with compound **1** in living PC-3 cells, 15 min incubation at 2 μM, 20 min incubation at 37 °C (λex. 488 nm).

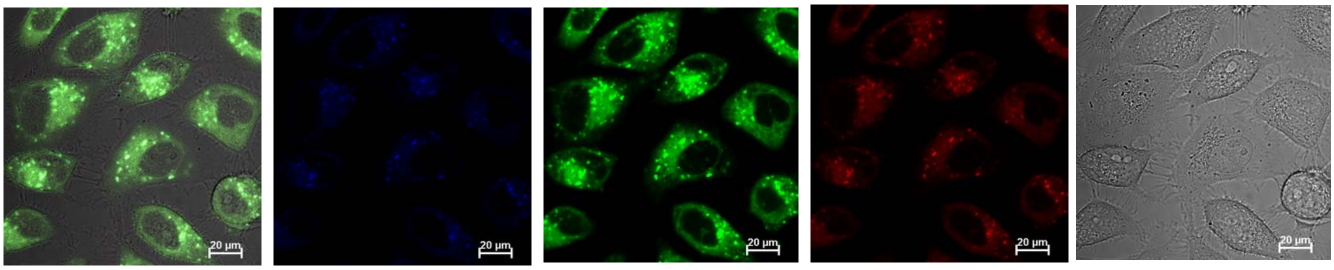

Figure S59. Confocal Fluorescence imaging with (un-protected) compound **1** in living PC-3 cells, 20 min incubation at 250 nM, 20 min incubation at 37 °C (λex. 405 nm).

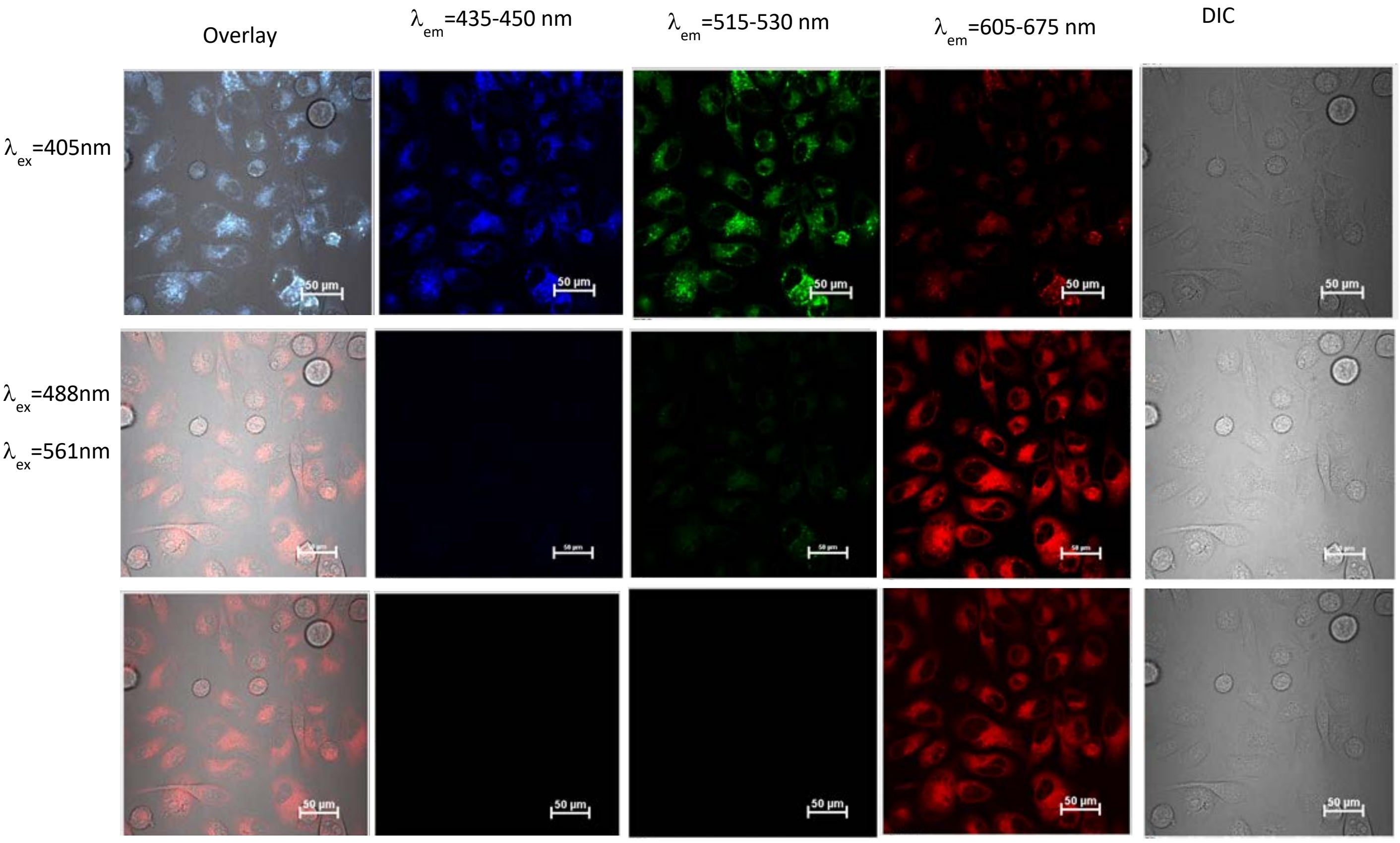

Figure S60. Laser confocal fluorescence imaging of (un-protected) Compound **1**, 250 nM, 20 min incubation at 37 °C. Co-localisation experiment with Nile Red stain.

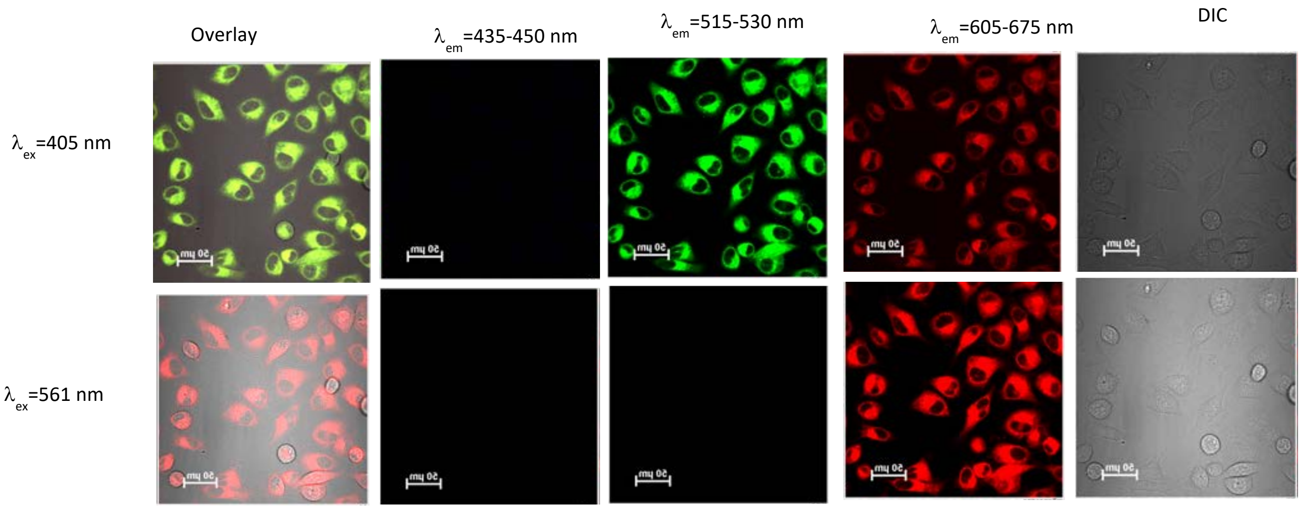

Figure S61. Laser confocal fluorescence imaging of Compound **3**, 1μM, 20 min incubation at 37 °C. Co-localisation experiment with Nile Red stain.

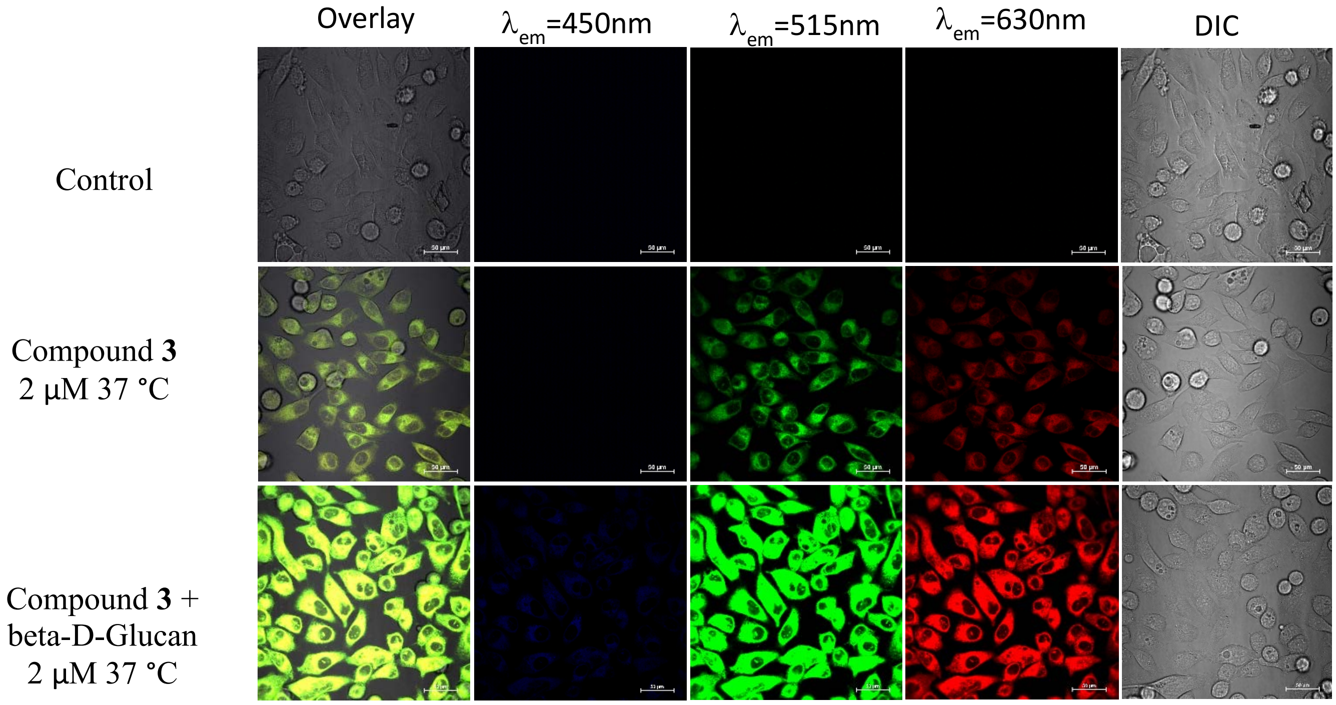

Figure S62. Confocal Fluorescence imaging with compound **3-BPin** in living PC-3, 15 min incubation under various conditions (λex. 488 nm)

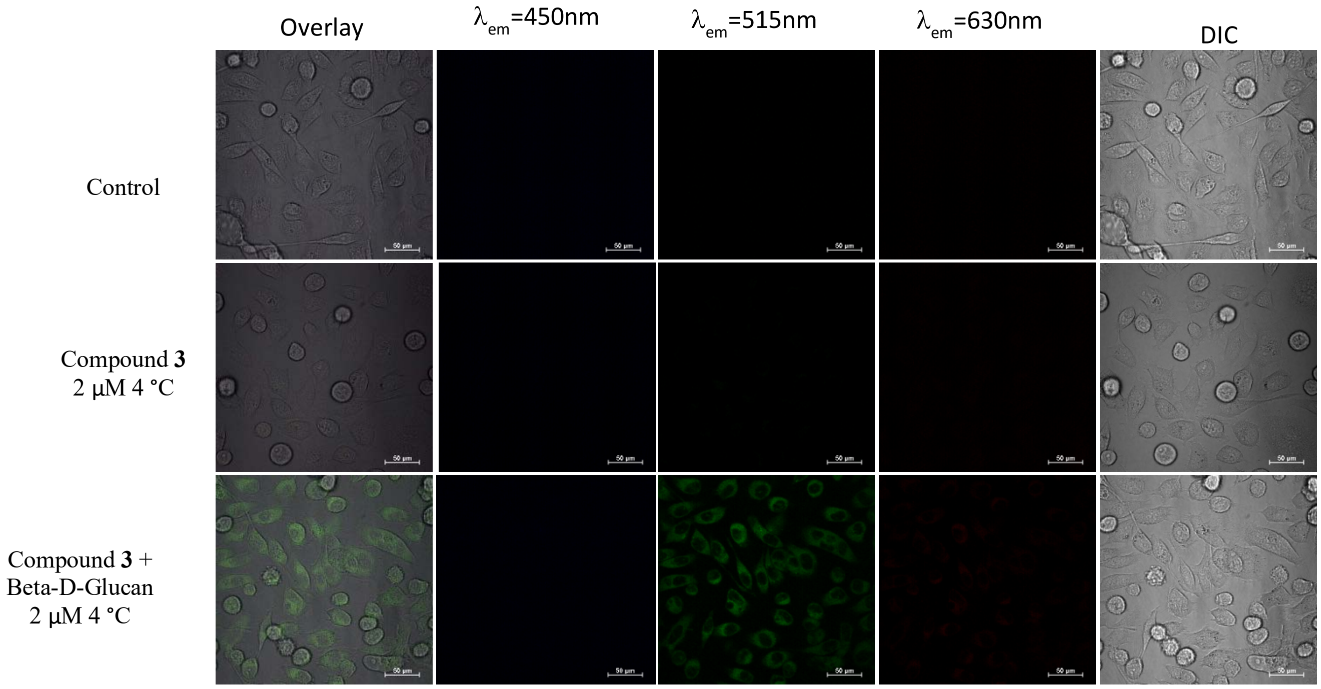

Figure S63. Confocal Fluorescence imaging with compound **3-BPin** in living PC-3 cells, 15 min incubation under various conditions (15 min incubation, λex. 405 nm)

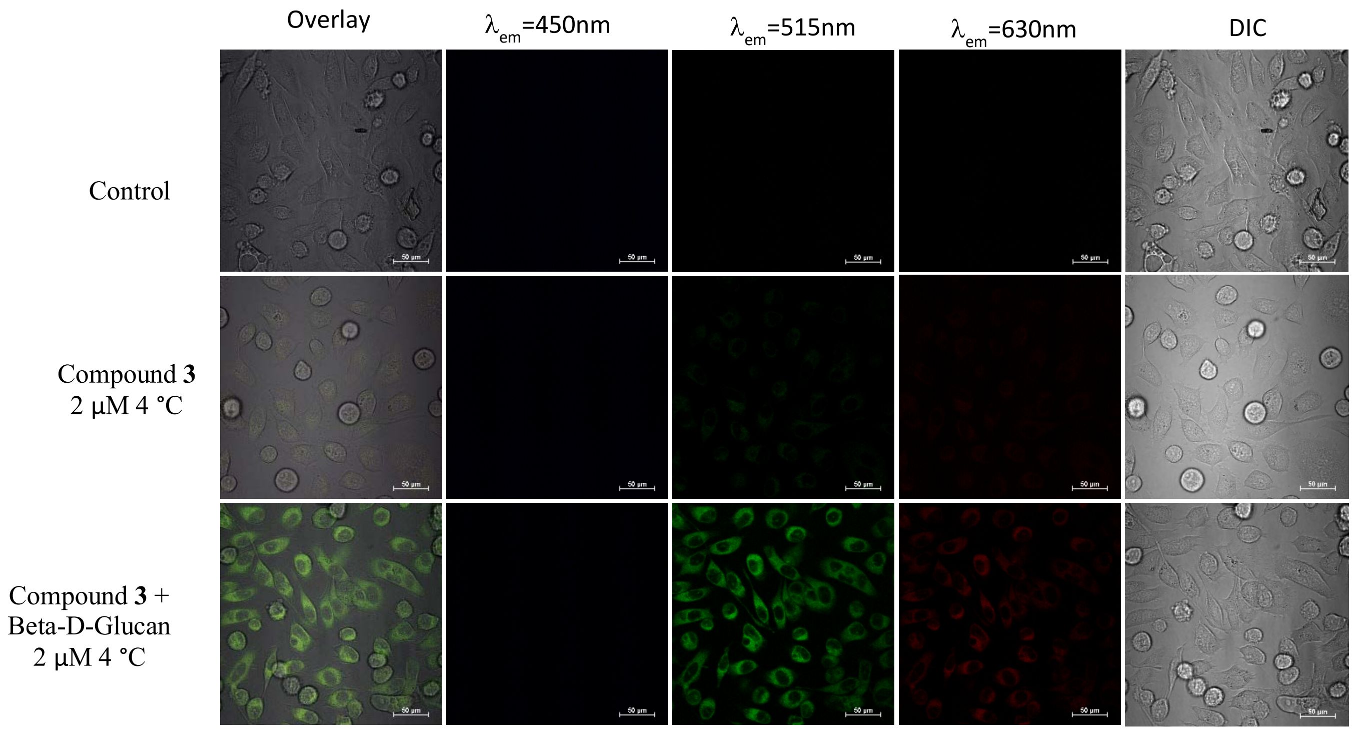

Figure S64 Confocal Fluorescence imaging with compound **3-BPin** in living PC3 15 min incubation under various conditions (λex 488 nm, 0.2% DMSO)

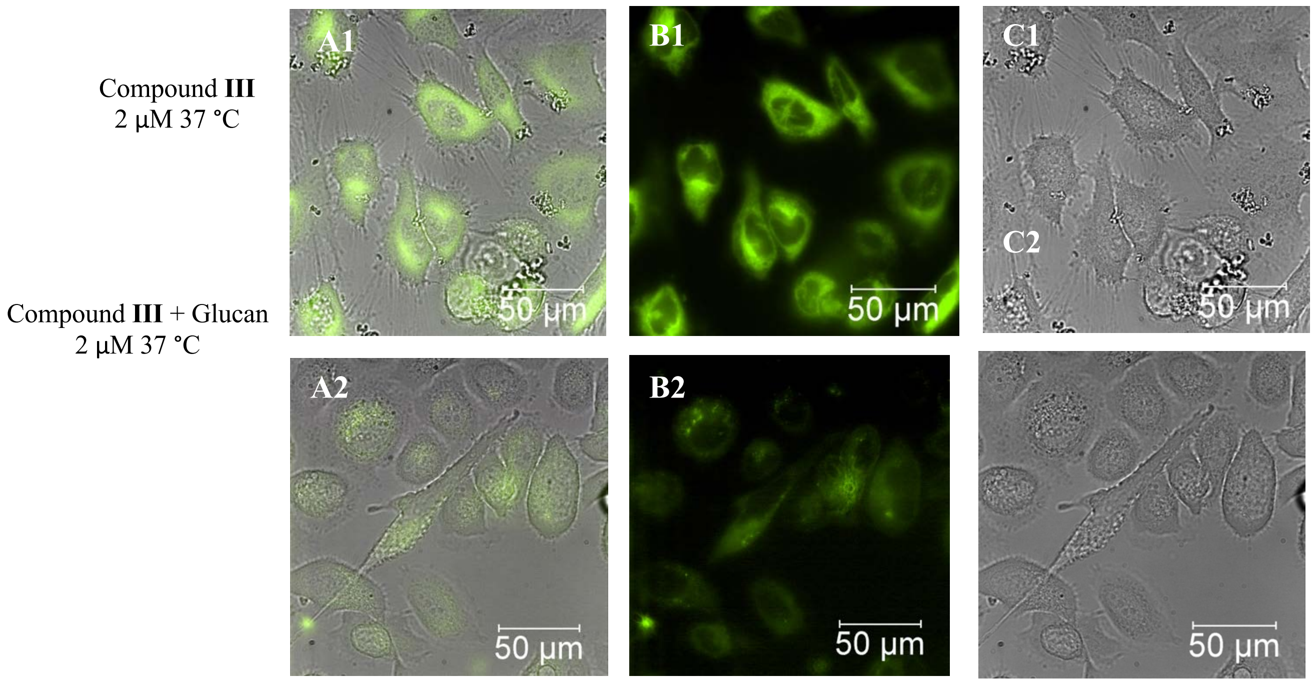

Figure S65. Epifluorescence imaging with compound **III-BPin** in living HeLa cells under 15 min incubation, 37 ºC, 2 μM probe **III-BPin** 0.2% DMSO, in presence, and absence of excess beta-D-glucan. Micrographs show: A(1-2) overlay images of B(1-2) and C(1-2), respectively; (B1-2) fluorescence images with λ_{ex} = 460–500 nm, long-pass filtered at 510 nm, showing the biolocalisation of 1 throughout the cytoplasm; (C1-2) brightfield images

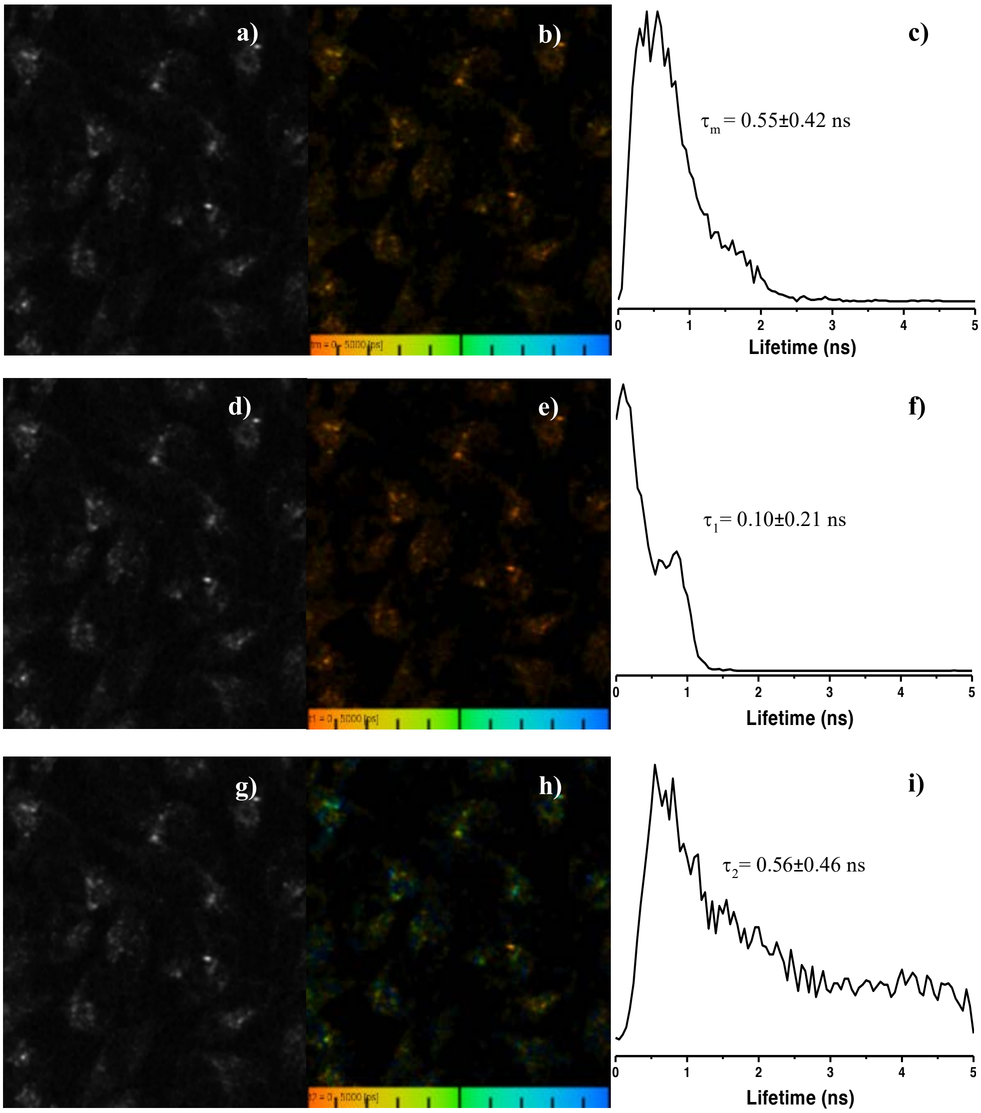

Figure S66. 2-photon FLIM control experiments aiming to evaluate effect of auto-fluorescence (1% DMSO, 15 min incubation at 37 °C in living HeLa cells, 2P excitation, λex=910 nm)

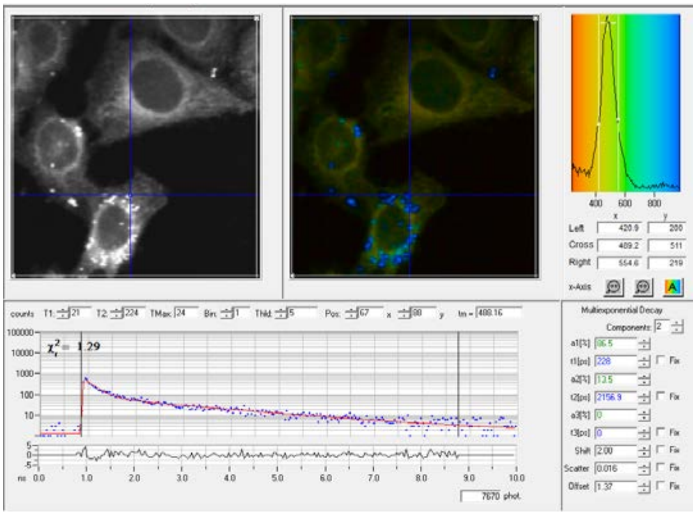

(a)

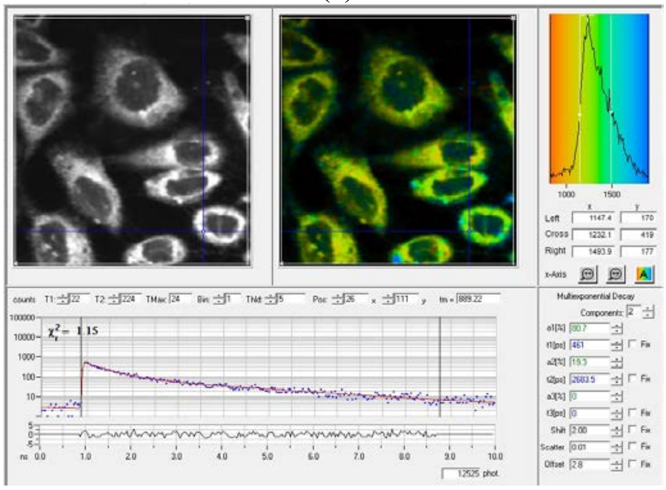

(b)

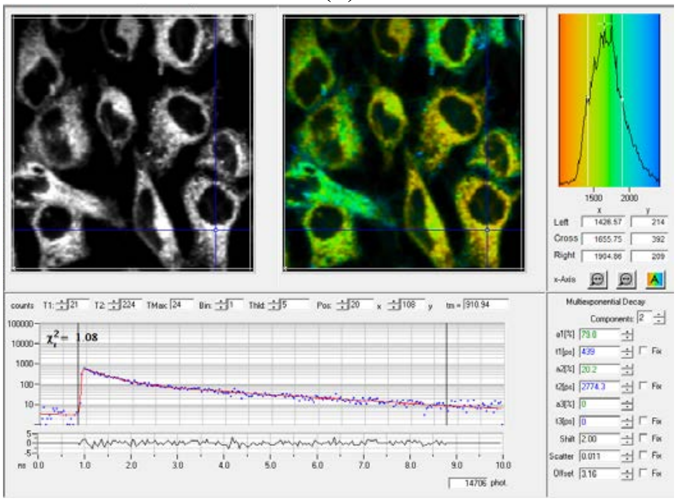

(c)

Figure S67. Cellular fluorescence intensity and lifetime maps, selected lifetime decay plot with data fit. For 2-photon FLIM images, cells were treated with 100 µM of compounds DMSO, ex = 910 nm where: (a) **compound 2-BPin**, 37 ºC, 1% DMSO. (b) **compound 2@glucan**, 37 ºC, 0.5% DMSO, excess beta-D-Glucan c) **compound 2-BPin**, 4 ºC, in 1% DMSO. Micrographs are showing the two-photon fluorescence intensity diagram of compounds, the fluorescence lifetime map of t_m of compounds in HeLa cells and corresponding fitted parameters.

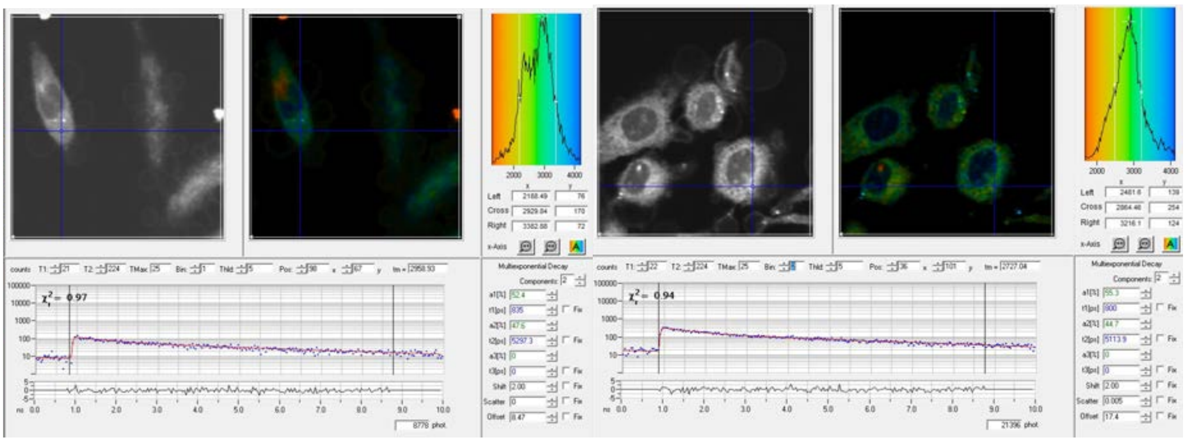

(a) (b)

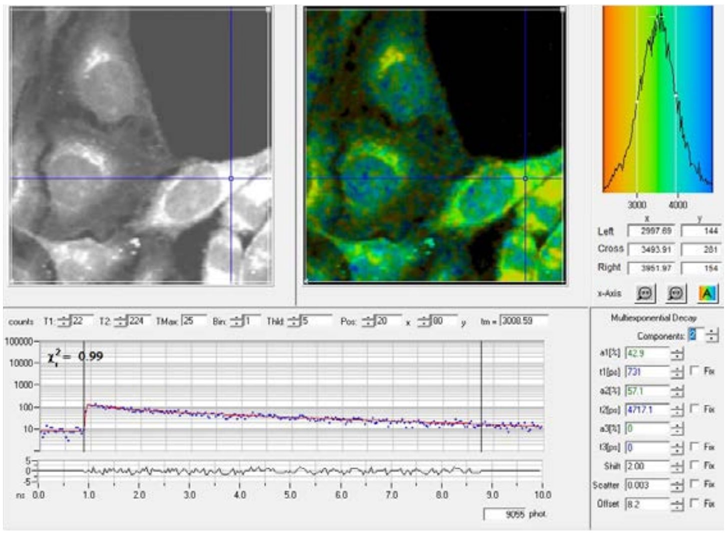

(c)

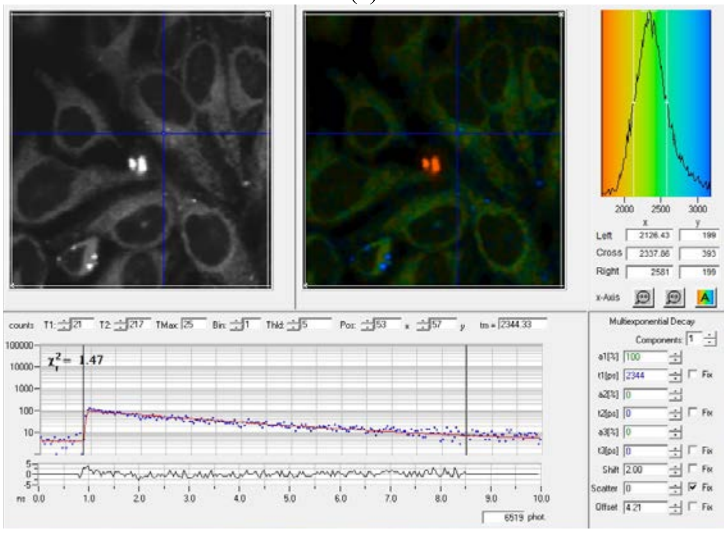

(d)

Figure S68. Cellular fluorescence intensity and lifetime maps, selected lifetime decay plot with data fit. For 2-photon FLIM images, cells were treated with 100 μM of compounds. Cells were incubated at 37 ºC or 4 ºC where micrographs (a) and (b) represent different fields of view for **1-BPin**, (37 ºC, λex = 910 nm, 1% DMSO and post-PBS washing); (c) **1@glucan** (37 ºC, λex = 910 nm, 0.5 % DMSO and excess beta-D-glucan); (d) **1-BPin**, 4 ºC, λex = 910 nm, 1% DMSO, post-PBS washing.

5. MTT assays in PC-3 cells

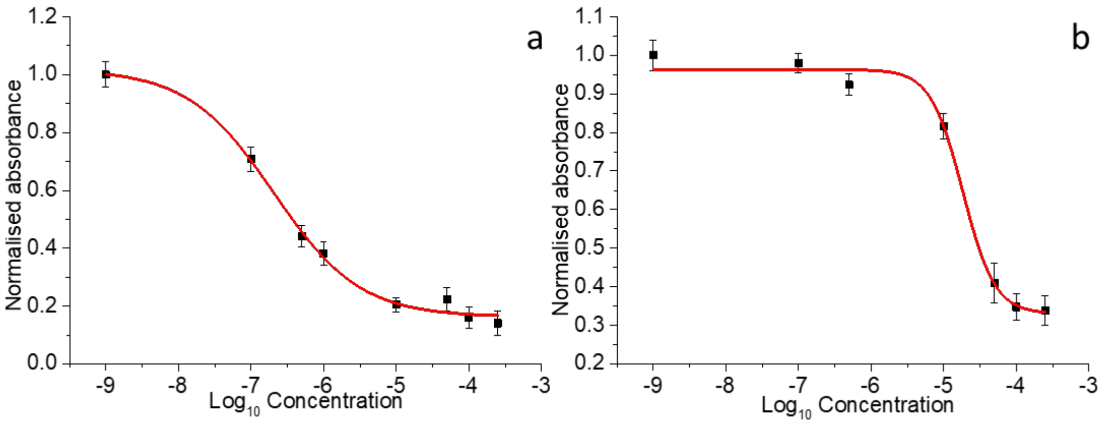

Figure

Compound **1-BPin**: IC_{50}=2.06536×10^{-7}±9.7116×10^{-8} M

Compound **2-BPin:** IC_{50}=1.83593×10^{-5}±3.6745×10^{-6} M

Figure S69. MTT assays of PC3 cells treated with (a) **1-BPin** and (b) **2-BPin** showing their dose response curves at 48 h. Error bars stand for standard error calculated from the six independent repeats.

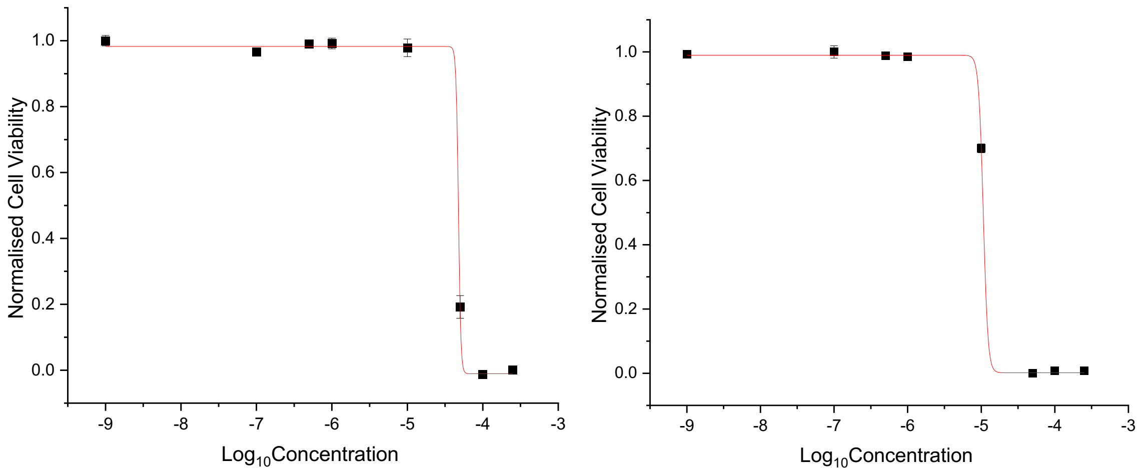

24 h: $IC_{50} = 4.75667 \times 10^{-5} \pm 0.80307 \times 10^{-6}$ M
48 h: $IC_{50} = 1.06607 \times 10^{-5} \pm 0.22857 \times 10^{-6}$ M

Figure S70. MTT assays of PC3 cells treated with **3-BPin** at (a) 24 hours and (b) 48 hours showing the dose response curve. Error bars stand for standard error calculated from six independent repeats.

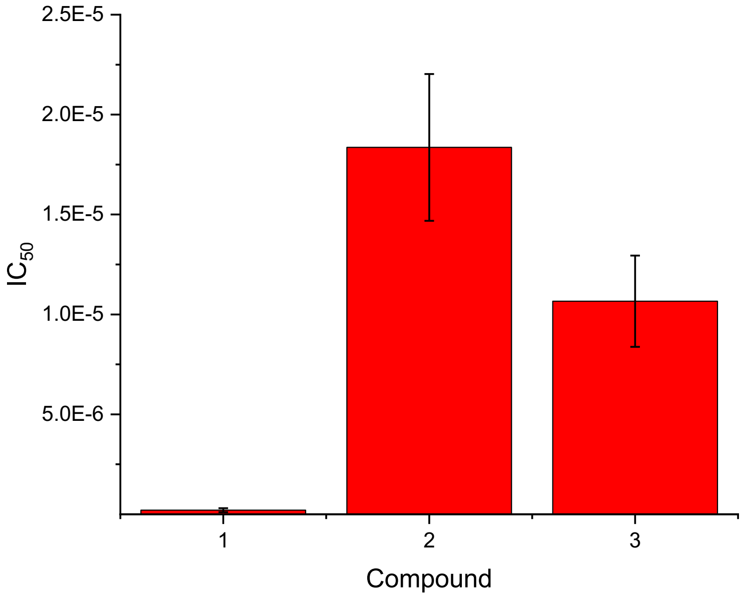

Figure S71. A comparison of IC_{50} values (M) from MTT assays in PC3 cells, treated with compounds **1-BPin**, **2-BPin** and **3-BPin** (48 h assays). Error bars stand for standard error calculated from the six repeats.

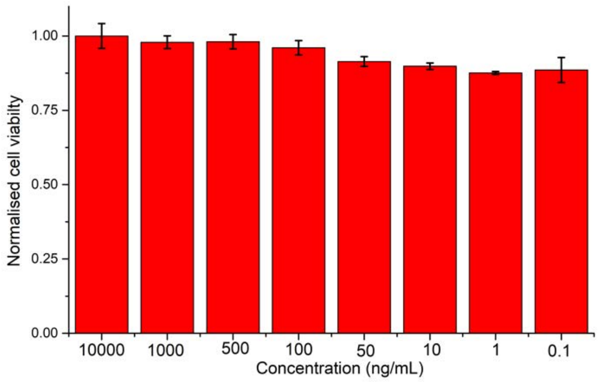

Figure S72. MTT assays of PC3 cells treated with **beta-D-glucan** for 48 hours showing that this biopolymer is non-toxic in the interval tested. Error bars stand for standard error calculated from the six repeats.

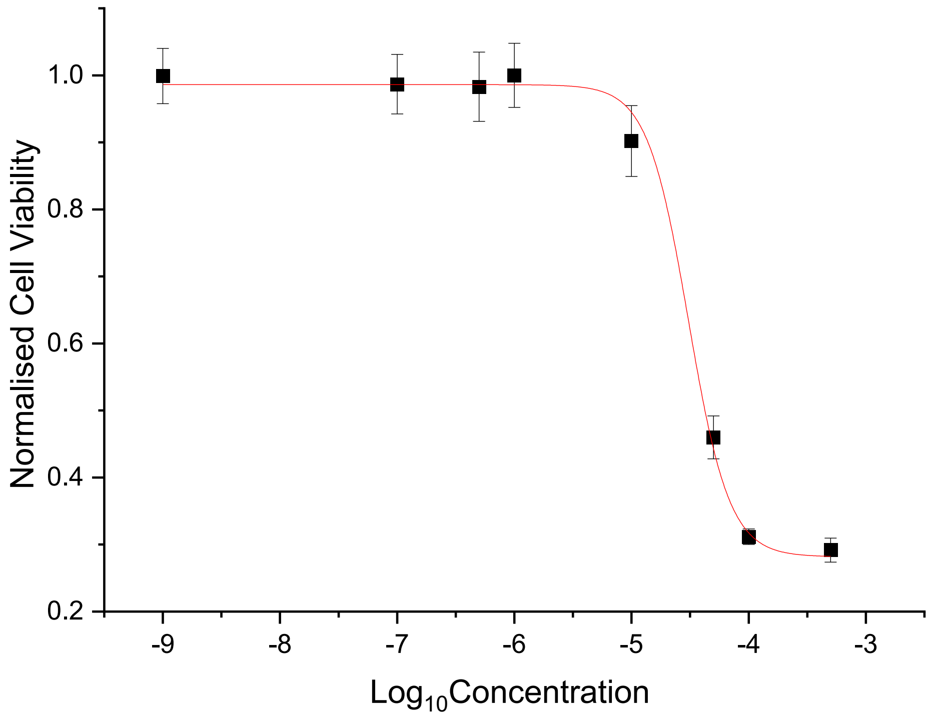

$IC_{50} = 3.06415 \times 10^{-5} \pm 3.258816 \times 10^{-6}$ M

Figure S73. MTT assays of PC3 cells treated with **cis-platin** $[Pt(Cl)_2(NH_3)_2]$ for 48 hours showing the dose response curve. Error bars stand for standard error calculated from the six repeats.

6. Structural Investigations of Compound 3-BPin by Gas Phase DFT Calculations

Optimised geometry of 3-BPin

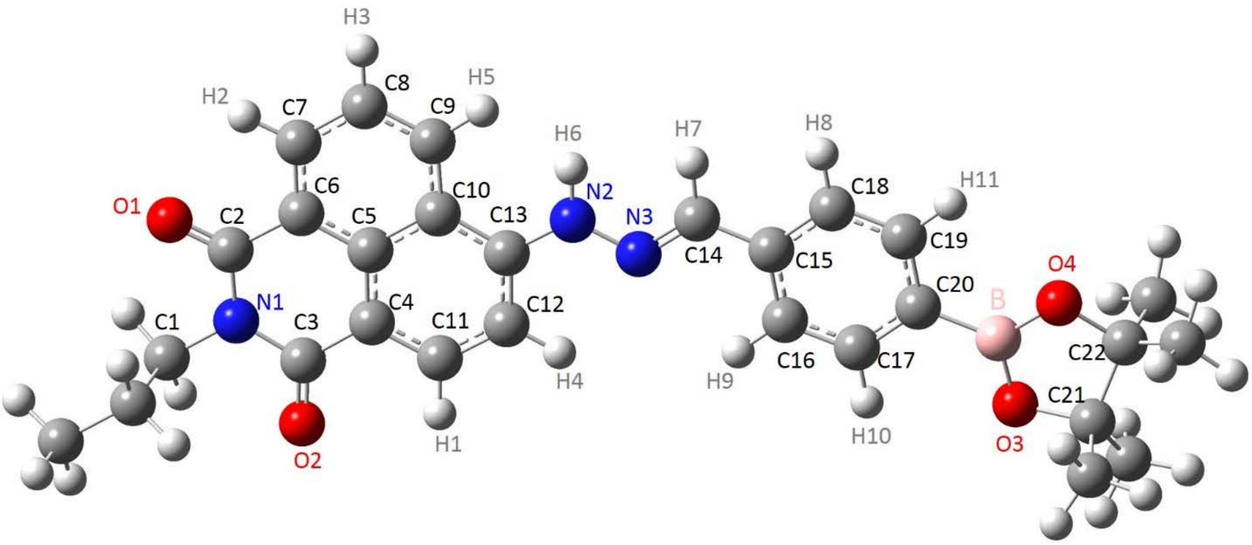

Figure S74. Relaxed, optimised geometry of naphthalimide boronate with pinacol-protected boronic acid group.

Table S3. Selected bond lengths and bond angles in the optimised structure of naphthalimide boronate with pinacol-protected boronic acid group.

Bond type	Bond length (Å)		Angle type	Bond angle (°)	
	X-ray (estimated using CCDC Mercury software – full structural parameters are given below)	DFT Calculated		X-ray (estimated using CCDC Mercury software – full structural parameters are given below)	DFT Calculated
C1-N1	1.48	1.47	C1-N1-C2	117.7	117.4
C2-N1	1.39	1.41	C1-N1-C3	117.6	117.4
C3-N1	1.40	1.42	N1-C2-O1	120.2	120.0
C2-O1	1.22	1.24	N1-C3-O2	119.4	120.0
C3-O2	1.22	1.24	C13-N2-H6	121.5	119.1
C2-C6	1.47	1.49	C13-N2-N3	120.4	122.1
C3-C4	1.46	1.48	H6-N2-N3	117.9	118.8
C13-N2	1.36	1.38	N2-N3-C14	113.8	117.1
N2-N3	1.38	1.35	C20-B-O3	121.2	123.0
N3-C14	1.28	1.30	C20-B-O4	124.9	123.2
N2-H6	0.88	1.02	O3-B-O4	113.9	113.8
B-C20	1.55	1.56	B-O3-C21	106.2	106.6

B-O4	1.37	1.38	B-O4-C22	107.0	106.6
B-O3	1.36	1.38			
C21-O3	1.47	1.46			
C22-O4	1.46	1.46			
C21-C22	1.56	1.58			

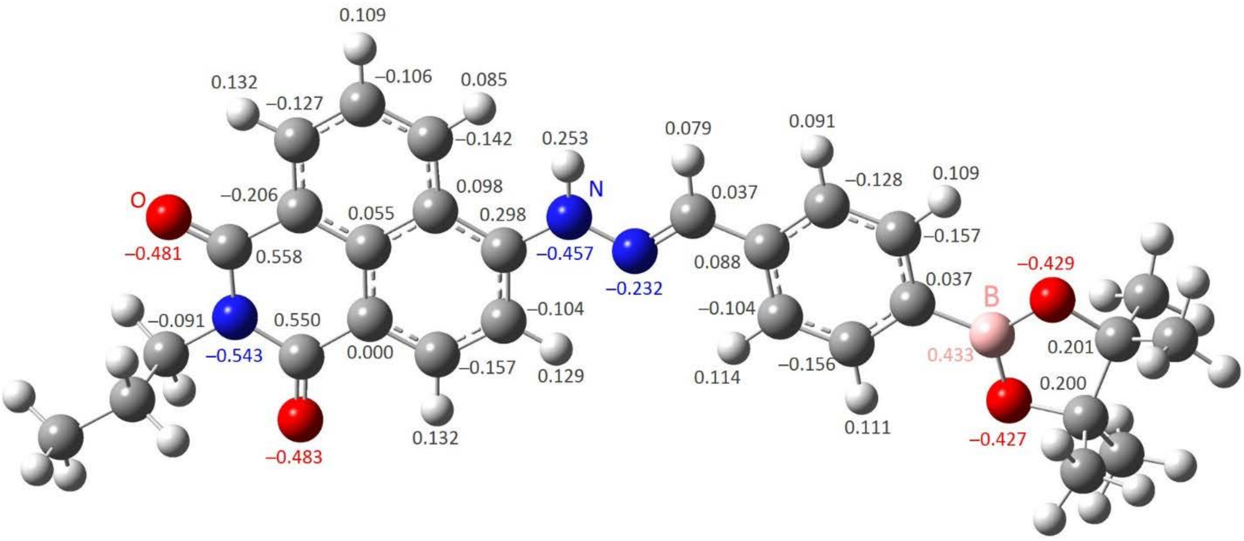

Figure S75. Mulliken charges on each atoms in the relaxed structure of napthalimide boronate with pinacol-protected boronic acid group.

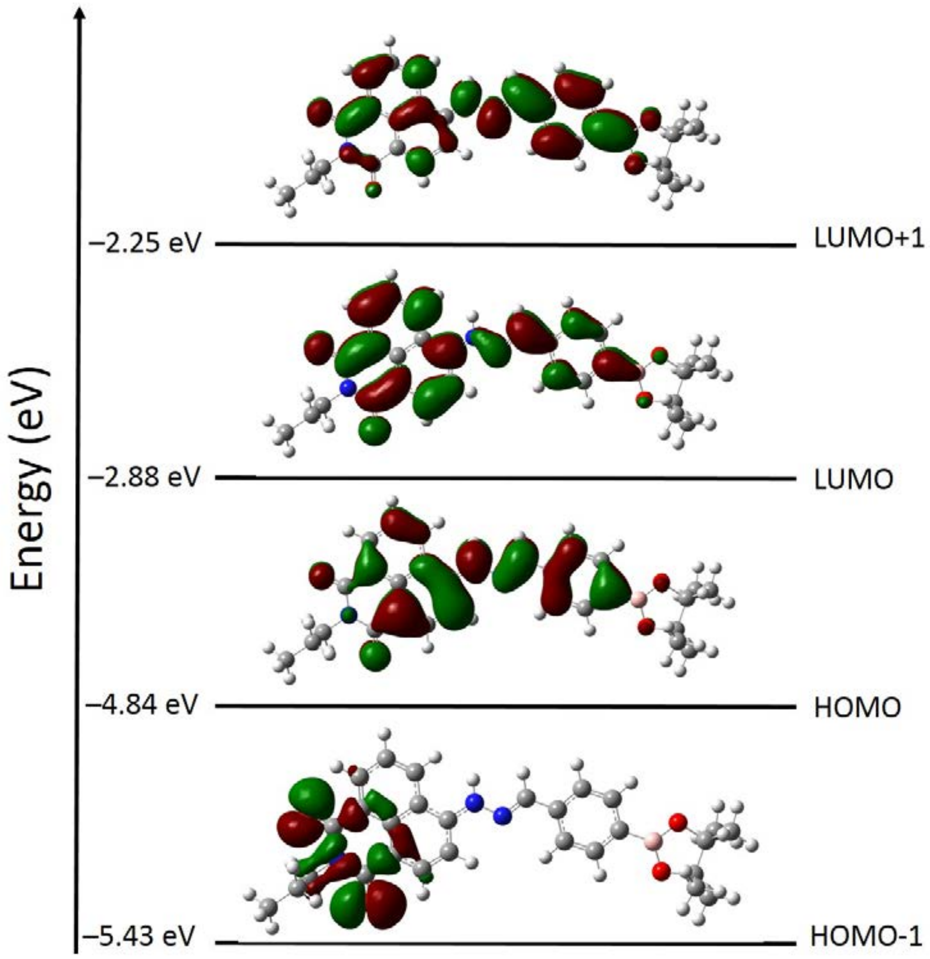

Figure S76. DFT calculated shapes and energies of frontier orbitals of naphthalimide boronate with pinacol-protected boronic acid group.

Naphthalimide boronate -deprotected compound 3

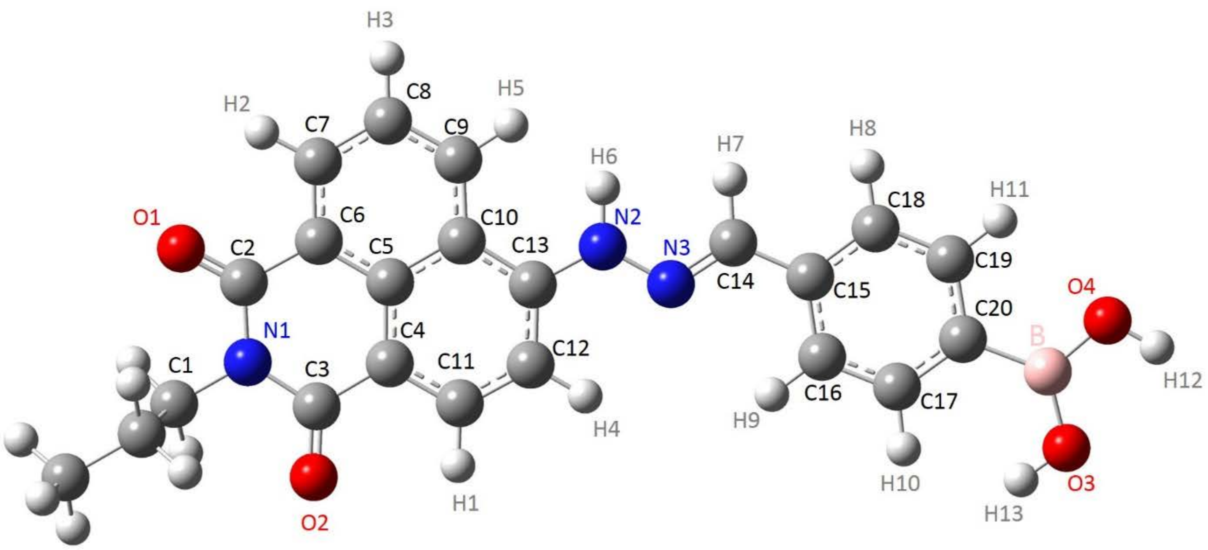

Figure S77. Relaxed, optimised geometry of napthalimide boronate with boronic acid group.

Table S4. Selected bond lengths and bond angles in the optimised structure of napthalimide boronate with boronic acid group.

Bond type	Bond length (Å)	Angle type	Bond angle (°)
C1-N1	1.47	C1-N1-C2	117.5
C2-N1	1.41	C1-N1-C3	117.3
C3-N1	1.42	N1-C2-O1	121.0
C2-O1	1.24	N1-C3-O2	120.3
C3-O2	1.24	C13-N2-H6	119.1
C2-C6	1.49	C13-N2-N3	122.1
C3-C4	1.48	H6-N2-N3	118.8
C13-N2	1.39	N2-N3-C14	117.1
N2-N3	1.35	C20-B-O3	124.4
N3-C14	1.20	C20-B-O4	117.9
N2-H6	1.03	O3-B-O4	117.7
B-C20	1.57	B-O3-H13	112.6
B-O4	1.38	B-O4-H12	110.1
B-O3	1.38		
O3-H13	0.97		
O4-H12	0.97		

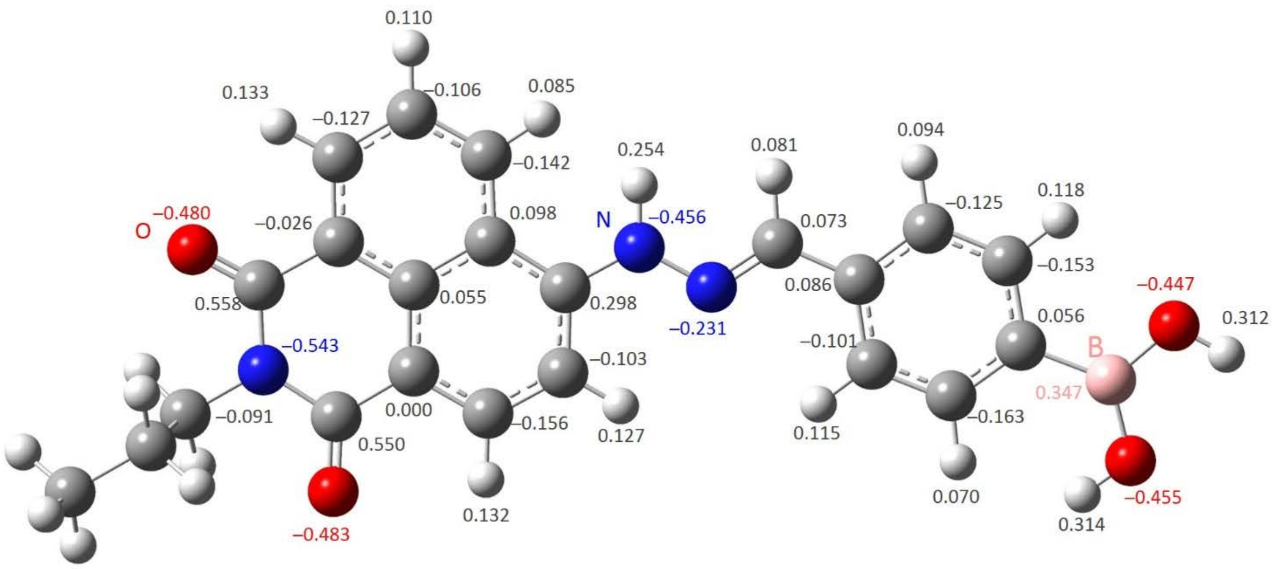

Figure S78. Mulliken charges on each atoms in the relaxed structure of napthalimide boronate with boronic acid group.

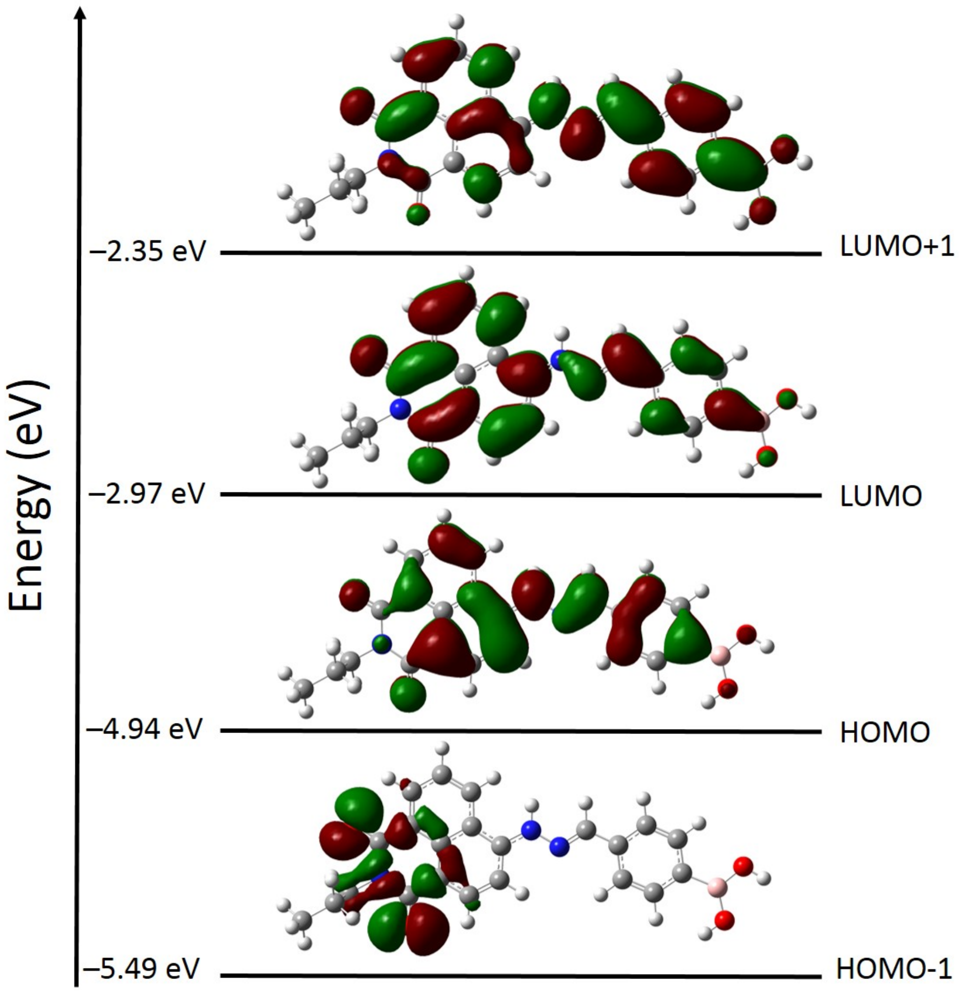

Figure S79. DFT calculated shapes and energies of frontier orbitals of napthalimide boronate with boronic acid group.

Table S5. Total energies of naphthalimide boronate with pinacol-protected boronic acid group (3-BPin), deprotected naphathalimide boronate, naphthalimide boronic acid (3), pinacol molecule (Pin) and water (H_2O).

Structure	Energy			
	Hartree	$kJmol^{-1}$	$kcalmol^{-1}$	eV
3-BPin	–1573.33	4130778.23	987279.69	42812.51
3	–1338.97	3515466.00	840216.54	36435.25
Pinacol	–387.04	1016173.60	242871.32	10531.89
H_2O	–76.33	200404.43	47897.81	2077.05

Reaction considered: *3* + *Pin* → *3-BPin* + *2* H_2O

Reaction energy = –0.02 Hartree/–52.51 $kJmol^{-1}$/–12.6 $kcalmol^{-1}$/–0.54 eV

7. Structural Investigations of 3-BPin: Single Crystal X-ray Diffraction Analysis

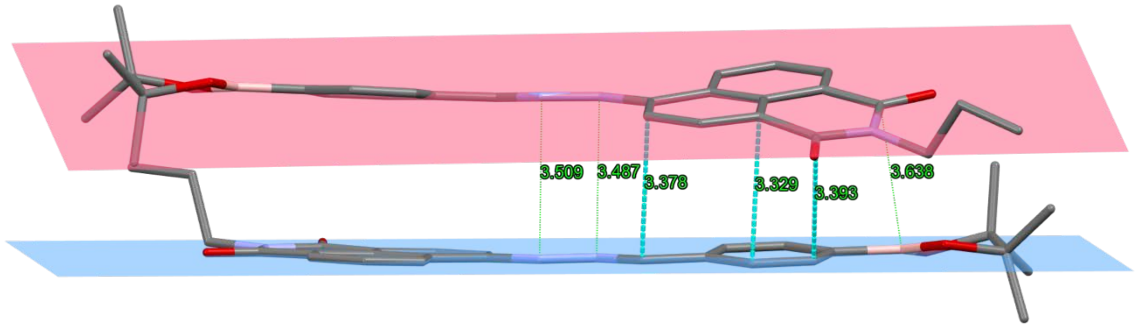

Figure 80. Best planes orientations and shortest contacts (close to the sum of van der Waals radii) for the two compound molecules in the asymmetric unit of Compound **3-BPin**.

Table S6. X-Ray Crystal data for Compound **3-BPin.**

Identification code	s23sip1	
Empirical formula	C30 H36 B N3 O5 S	
Formula weight	561.49	
Temperature	150.01(10) K	
Wavelength	1.54184 Å	
Crystal system	Monoclinic	
Space group	$P2_1/c$	
Unit cell dimensions	a = 20.82821(9) Å	a= 90°.
	b = 11.60042(6) Å	b= 108.6189(5)°.
	c = 26.05412(12) Å	g = 90°.
Volume	5965.62(5) $Å^3$	
Z	8	
Density (calculated)	1.250 Mg/m^3	
Absorption coefficient	1.310 mm^{-1}	
F(000)	2384	
Crystal size	0.430 x 0.170 x 0.110 mm^3	
Theta range for data collection	4.211 to 72.931°.	
Index ranges	-25<=h<=25, -14<=k<=10, -32<=l<=32	
Reflections collected	134828	
Independent reflections	11867 [R(int) = 0.0359]	
Completeness to theta = 67.684°	100.0 %	
Absorption correction	Semi-empirical from equivalents	
Max. and min. transmission	1.00000 and 0.74517	
Refinement method	Full-matrix least-squares on F^2	

Data / restraints / parameters	11867 / 6 / 865
Goodness-of-fit on F^2	1.084
Final R indices [I>2sigma(I)]	R1 = 0.0419, wR2 = 0.1078
R indices (all data)	R1 = 0.0469, wR2 = 0.1112
Extinction coefficient	n/a
Largest diff. peak and hole	0.273 and -0.316 e.Å^{-3}

Table S7. Bond lengths [Å] for Compound 3-BPin.

O(1)-C(4)	1.2233(18)
O(2)-C(14)	1.2219(17)
O(3)-B(1)	1.367(2)
O(3)-C(23)	1.4652(18)
O(4)-B(1)	1.364(2)
O(4)-C(26)	1.4601(16)
B(1)-C(20)	1.553(2)
N(1)-C(14)	1.3905(19)
N(1)-C(4)	1.4041(18)
N(1)-C(3)	1.4752(18)
N(2)-C(8)	1.3547(19)
N(2)-N(3)	1.3763(17)
N(2)-H(2)	0.88(2)
N(3)-C(16)	1.2817(19)
C(1)-C(2)	1.527(2)
C(1)-H(1A)	0.9800
C(1)-H(1B)	0.9800
C(1)-H(1C)	0.9800
C(2)-C(3)	1.514(3)
C(2)-H(2A)	0.9900
C(2)-H(2B)	0.9900
C(3)-H(3A)	0.9900
C(3)-H(3B)	0.9900
C(4)-C(5)	1.4540(19)
C(5)-C(6)	1.3861(19)
C(5)-C(15)	1.4129(19)
C(6)-C(7)	1.384(2)
C(6)-H(6)	0.9500
C(7)-C(8)	1.396(2)
C(7)-H(7)	0.9500
C(8)-C(9)	1.4430(19)
C(9)-C(10)	1.411(2)
C(9)-C(15)	1.4172(19)
C(10)-C(11)	1.375(2)
C(10)-H(10)	0.9500
C(11)-C(12)	1.393(2)
C(11)-H(11)	0.9500
C(12)-C(13)	1.375(2)

C(12)-H(12)	0.9500
C(13)-C(15)	1.4166(18)
C(13)-C(14)	1.474(2)
C(16)-C(17)	1.456(2)
C(16)-H(16)	0.9500
C(17)-C(22)	1.396(2)
C(17)-C(18)	1.399(2)
C(18)-C(19)	1.379(2)
C(18)-H(18)	0.9500
C(19)-C(20)	1.402(2)
C(19)-H(19)	0.9500
C(20)-C(21)	1.398(2)
C(21)-C(22)	1.381(2)
C(21)-H(21)	0.9500
C(22)-H(22)	0.9500
C(23)-C(25)	1.516(2)
C(23)-C(24)	1.517(2)
C(23)-C(26)	1.558(2)
C(24)-H(24A)	0.9800
C(24)-H(24B)	0.9800
C(24)-H(24C)	0.9800
C(25)-H(25A)	0.9800
C(25)-H(25B)	0.9800
C(25)-H(25C)	0.9800
C(26)-C(28)	1.514(2)
C(26)-C(27)	1.514(2)
C(27)-H(27A)	0.9800
C(27)-H(27B)	0.9800
C(27)-H(27C)	0.9800
C(28)-H(28A)	0.9800
C(28)-H(28B)	0.9800
C(28)-H(28C)	0.9800
O(7)-B(2)	1.3672(18)
O(7)-C(53)	1.4595(16)
O(8)-B(2)	1.3636(19)
O(8)-C(56)	1.4700(15)
B(2)-C(50)	1.5594(19)
N(5)-C(40)	1.3664(18)
N(5)-N(6)	1.3768(15)
N(5)-H(5)	0.87(2)
N(6)-C(46)	1.2801(19)
N(4)-C(34)	1.394(6)
N(4)-C(44)	1.397(6)
N(4)-C(33)	1.470(4)
C(31)-C(32)	1.502(8)
C(31)-H(31A)	0.9800
C(31)-H(31B)	0.9800
C(31)-H(31C)	0.9800
C(32)-C(33)	1.514(4)
C(32)-H(32A)	0.9900

C(32)-H(32B)	0.9900
C(33)-H(33A)	0.9900
C(33)-H(33B)	0.9900
C(34)-O(5)	1.215(7)
C(34)-C(35)	1.503(5)
O(6)-C(44)	1.224(6)
C(44)-C(43)	1.508(5)
N(4A)-C(34A)	1.376(11)
N(4A)-C(44A)	1.416(11)
N(4A)-C(33A)	1.457(6)
C(31A)-C(32A)	1.63(2)
C(31A)-H(31D)	0.9800
C(31A)-H(31E)	0.9800
C(31A)-H(31F)	0.9800
C(32A)-C(33A)	1.487(8)
C(32A)-H(32C)	0.9900
C(32A)-H(32D)	0.9900
C(33A)-H(33C)	0.9900
C(33A)-H(33D)	0.9900
C(34A)-O(5A)	1.222(13)
C(34A)-C(35)	1.474(10)
O(6A)-C(44A)	1.214(13)
C(44A)-C(43)	1.421(11)
C(35)-C(36)	1.373(2)
C(35)-C(45)	1.4174(18)
C(36)-C(37)	1.394(2)
C(36)-H(36)	0.9500
C(37)-C(38)	1.375(2)
C(37)-H(37)	0.9500
C(38)-C(39)	1.412(2)
C(38)-H(38)	0.9500
C(39)-C(45)	1.4193(19)
C(39)-C(40)	1.4412(18)
C(40)-C(41)	1.389(2)
C(41)-C(42)	1.391(2)
C(41)-H(41)	0.9500
C(42)-C(43)	1.3822(19)
C(42)-H(42)	0.9500
C(43)-C(45)	1.409(2)
C(46)-C(47)	1.4636(18)
C(46)-H(46)	0.9500
C(47)-C(52)	1.3911(19)
C(47)-C(48)	1.3989(19)
C(48)-C(49)	1.3851(18)
C(48)-H(48)	0.9500
C(49)-C(50)	1.4037(18)
C(49)-H(49)	0.9500
C(50)-C(51)	1.3967(19)
C(51)-C(52)	1.3833(18)
C(51)-H(51)	0.9500

C(52)-H(52)	0.9500
C(53)-C(54)	1.517(2)
C(53)-C(55)	1.520(3)
C(53)-C(56)	1.555(2)
C(54)-H(54A)	0.9800
C(54)-H(54B)	0.9800
C(54)-H(54C)	0.9800
C(55)-H(55A)	0.9800
C(55)-H(55B)	0.9800
C(55)-H(55C)	0.9800
C(56)-C(57)	1.514(2)
C(56)-C(58)	1.516(2)
C(57)-H(57A)	0.9800
C(57)-H(57B)	0.9800
C(57)-H(57C)	0.9800
C(58)-H(58A)	0.9800
C(58)-H(58B)	0.9800
C(58)-H(58C)	0.9800
S(1)-O(9)	1.5010(14)
S(1)-C(62)	1.7796(19)
S(1)-C(61)	1.784(2)
C(62)-H(62A)	0.9800
C(62)-H(62B)	0.9800
C(62)-H(62C)	0.9800
S(1A)-O(9)	1.514(7)
S(1A)-C(62A)	1.75(5)
S(1A)-C(61)	1.869(8)
C(62A)-H(62D)	0.9800
C(62A)-H(62E)	0.9800
C(62A)-H(62F)	0.9800
C(61)-H(61A)	0.9800
C(61)-H(61B)	0.9800
C(61)-H(61C)	0.9800
C(61)-H(61D)	0.9800
C(61)-H(61E)	0.9800
C(61)-H(61F)	0.9800
S(2)-O(10)	1.511(6)
S(2)-C(64)	1.765(3)
S(2)-C(63)	1.773(3)
C(64)-H(64A)	0.9800
C(64)-H(64B)	0.9800
C(64)-H(64C)	0.9800
S(2A)-O(10A)	1.439(19)
S(2A)-C(64A)	1.658(17)
S(2A)-C(63)	1.686(3)
C(64A)-H(64D)	0.9800
C(64A)-H(64E)	0.9800
C(64A)-H(64F)	0.9800
C(63)-H(63A)	0.9800
C(63)-H(63B)	0.9800

C(63)-H(63C)	0.9800
C(63)-H(63D)	0.9800
C(63)-H(63E)	0.9800
C(63)-H(63F)	0.9800

Table S8. Bond angles [°] for 3-BPin.

B(1)-O(3)-C(23)	106.21(12)
B(1)-O(4)-C(26)	107.00(11)
O(4)-B(1)-O(3)	113.91(14)
O(4)-B(1)-C(20)	121.19(14)
O(3)-B(1)-C(20)	124.90(14)
C(14)-N(1)-C(4)	124.65(12)
C(14)-N(1)-C(3)	117.65(12)
C(4)-N(1)-C(3)	117.58(12)
C(8)-N(2)-N(3)	120.41(13)
C(8)-N(2)-H(2)	121.5(13)
N(3)-N(2)-H(2)	117.9(13)
C(16)-N(3)-N(2)	113.80(13)
C(2)-C(1)-H(1A)	109.5
C(2)-C(1)-H(1B)	109.5
H(1A)-C(1)-H(1B)	109.5
C(2)-C(1)-H(1C)	109.5
H(1A)-C(1)-H(1C)	109.5
H(1B)-C(1)-H(1C)	109.5
C(3)-C(2)-C(1)	111.48(15)
C(3)-C(2)-H(2A)	109.3
C(1)-C(2)-H(2A)	109.3
C(3)-C(2)-H(2B)	109.3
C(1)-C(2)-H(2B)	109.3
H(2A)-C(2)-H(2B)	108.0
N(1)-C(3)-C(2)	111.25(13)
N(1)-C(3)-H(3A)	109.4
C(2)-C(3)-H(3A)	109.4
N(1)-C(3)-H(3B)	109.4
C(2)-C(3)-H(3B)	109.4
H(3A)-C(3)-H(3B)	108.0
O(1)-C(4)-N(1)	119.40(13)
O(1)-C(4)-C(5)	123.57(13)
N(1)-C(4)-C(5)	117.02(12)
C(6)-C(5)-C(15)	119.01(13)
C(6)-C(5)-C(4)	119.98(13)
C(15)-C(5)-C(4)	121.00(12)
C(7)-C(6)-C(5)	121.88(13)
C(7)-C(6)-H(6)	119.1
C(5)-C(6)-H(6)	119.1
C(6)-C(7)-C(8)	120.53(13)
C(6)-C(7)-H(7)	119.7
C(8)-C(7)-H(7)	119.7

N(2)-C(8)-C(7)	121.69(13)
N(2)-C(8)-C(9)	118.84(13)
C(7)-C(8)-C(9)	119.47(13)
C(10)-C(9)-C(15)	118.36(12)
C(10)-C(9)-C(8)	123.31(13)
C(15)-C(9)-C(8)	118.33(12)
C(11)-C(10)-C(9)	121.07(14)
C(11)-C(10)-H(10)	119.5
C(9)-C(10)-H(10)	119.5
C(10)-C(11)-C(12)	120.55(14)
C(10)-C(11)-H(11)	119.7
C(12)-C(11)-H(11)	119.7
C(13)-C(12)-C(11)	120.06(13)
C(13)-C(12)-H(12)	120.0
C(11)-C(12)-H(12)	120.0
C(12)-C(13)-C(15)	120.65(13)
C(12)-C(13)-C(14)	119.32(12)
C(15)-C(13)-C(14)	120.03(12)
O(2)-C(14)-N(1)	120.23(13)
O(2)-C(14)-C(13)	122.47(14)
N(1)-C(14)-C(13)	117.30(12)
C(5)-C(15)-C(13)	119.94(13)
C(5)-C(15)-C(9)	120.76(12)
C(13)-C(15)-C(9)	119.30(12)
N(3)-C(16)-C(17)	122.41(14)
N(3)-C(16)-H(16)	118.8
C(17)-C(16)-H(16)	118.8
C(22)-C(17)-C(18)	118.98(13)
C(22)-C(17)-C(16)	117.68(14)
C(18)-C(17)-C(16)	123.34(14)
C(19)-C(18)-C(17)	120.24(14)
C(19)-C(18)-H(18)	119.9
C(17)-C(18)-H(18)	119.9
C(18)-C(19)-C(20)	121.29(14)
C(18)-C(19)-H(19)	119.4
C(20)-C(19)-H(19)	119.4
C(21)-C(20)-C(19)	117.89(14)
C(21)-C(20)-B(1)	119.04(14)
C(19)-C(20)-B(1)	123.07(14)
C(22)-C(21)-C(20)	121.22(14)
C(22)-C(21)-H(21)	119.4
C(20)-C(21)-H(21)	119.4
C(21)-C(22)-C(17)	120.37(14)
C(21)-C(22)-H(22)	119.8
C(17)-C(22)-H(22)	119.8
O(3)-C(23)-C(25)	109.10(14)
O(3)-C(23)-C(24)	106.88(13)
C(25)-C(23)-C(24)	110.61(14)
O(3)-C(23)-C(26)	102.33(11)
C(25)-C(23)-C(26)	113.91(14)

C(24)-C(23)-C(26)	113.38(14)
C(23)-C(24)-H(24A)	109.5
C(23)-C(24)-H(24B)	109.5
H(24A)-C(24)-H(24B)	109.5
C(23)-C(24)-H(24C)	109.5
H(24A)-C(24)-H(24C)	109.5
H(24B)-C(24)-H(24C)	109.5
C(23)-C(25)-H(25A)	109.5
C(23)-C(25)-H(25B)	109.5
H(25A)-C(25)-H(25B)	109.5
C(23)-C(25)-H(25C)	109.5
H(25A)-C(25)-H(25C)	109.5
H(25B)-C(25)-H(25C)	109.5
O(4)-C(26)-C(28)	108.16(12)
O(4)-C(26)-C(27)	106.29(12)
C(28)-C(26)-C(27)	110.26(13)
O(4)-C(26)-C(23)	102.34(11)
C(28)-C(26)-C(23)	114.95(13)
C(27)-C(26)-C(23)	114.01(13)
C(26)-C(27)-H(27A)	109.5
C(26)-C(27)-H(27B)	109.5
H(27A)-C(27)-H(27B)	109.5
C(26)-C(27)-H(27C)	109.5
H(27A)-C(27)-H(27C)	109.5
H(27B)-C(27)-H(27C)	109.5
C(26)-C(28)-H(28A)	109.5
C(26)-C(28)-H(28B)	109.5
H(28A)-C(28)-H(28B)	109.5
C(26)-C(28)-H(28C)	109.5
H(28A)-C(28)-H(28C)	109.5
H(28B)-C(28)-H(28C)	109.5
B(2)-O(7)-C(53)	107.30(11)
B(2)-O(8)-C(56)	106.45(11)
O(8)-B(2)-O(7)	113.71(12)
O(8)-B(2)-C(50)	125.71(13)
O(7)-B(2)-C(50)	120.59(13)
C(40)-N(5)-N(6)	120.10(12)
C(40)-N(5)-H(5)	122.8(13)
N(6)-N(5)-H(5)	115.4(13)
C(46)-N(6)-N(5)	114.39(12)
C(34)-N(4)-C(44)	124.9(3)
C(34)-N(4)-C(33)	117.0(3)
C(44)-N(4)-C(33)	118.1(3)
C(32)-C(31)-H(31A)	109.5
C(32)-C(31)-H(31B)	109.5
H(31A)-C(31)-H(31B)	109.5
C(32)-C(31)-H(31C)	109.5
H(31A)-C(31)-H(31C)	109.5
H(31B)-C(31)-H(31C)	109.5
C(31)-C(32)-C(33)	109.6(4)

C(31)-C(32)-H(32A)	109.8
C(33)-C(32)-H(32A)	109.8
C(31)-C(32)-H(32B)	109.8
C(33)-C(32)-H(32B)	109.8
H(32A)-C(32)-H(32B)	108.2
N(4)-C(33)-C(32)	113.1(3)
N(4)-C(33)-H(33A)	109.0
C(32)-C(33)-H(33A)	109.0
N(4)-C(33)-H(33B)	109.0
C(32)-C(33)-H(33B)	109.0
H(33A)-C(33)-H(33B)	107.8
O(5)-C(34)-N(4)	121.0(5)
O(5)-C(34)-C(35)	122.2(5)
N(4)-C(34)-C(35)	116.8(4)
O(6)-C(44)-N(4)	120.4(4)
O(6)-C(44)-C(43)	122.6(5)
N(4)-C(44)-C(43)	117.1(4)
C(34A)-N(4A)-C(44A)	124.4(7)
C(34A)-N(4A)-C(33A)	117.2(5)
C(44A)-N(4A)-C(33A)	118.4(6)
C(32A)-C(31A)-H(31D)	109.5
C(32A)-C(31A)-H(31E)	109.5
H(31D)-C(31A)-H(31E)	109.5
C(32A)-C(31A)-H(31F)	109.5
H(31D)-C(31A)-H(31F)	109.5
H(31E)-C(31A)-H(31F)	109.5
C(33A)-C(32A)-C(31A)	108.6(9)
C(33A)-C(32A)-H(32C)	110.0
C(31A)-C(32A)-H(32C)	110.0
C(33A)-C(32A)-H(32D)	110.0
C(31A)-C(32A)-H(32D)	110.0
H(32C)-C(32A)-H(32D)	108.3
N(4A)-C(33A)-C(32A)	114.5(6)
N(4A)-C(33A)-H(33C)	108.6
C(32A)-C(33A)-H(33C)	108.6
N(4A)-C(33A)-H(33D)	108.6
C(32A)-C(33A)-H(33D)	108.6
H(33C)-C(33A)-H(33D)	107.6
O(5A)-C(34A)-N(4A)	121.0(9)
O(5A)-C(34A)-C(35)	121.4(9)
N(4A)-C(34A)-C(35)	117.3(7)
O(6A)-C(44A)-N(4A)	118.1(9)
O(6A)-C(44A)-C(43)	124.2(9)
N(4A)-C(44A)-C(43)	117.3(8)
C(36)-C(35)-C(45)	120.89(13)
C(36)-C(35)-C(34A)	119.0(4)
C(45)-C(35)-C(34A)	118.6(4)
C(36)-C(35)-C(34)	118.5(2)
C(45)-C(35)-C(34)	120.2(2)
C(35)-C(36)-C(37)	120.14(13)

C(35)-C(36)-H(36)	119.9
C(37)-C(36)-H(36)	119.9
C(38)-C(37)-C(36)	120.36(14)
C(38)-C(37)-H(37)	119.8
C(36)-C(37)-H(37)	119.8
C(37)-C(38)-C(39)	121.22(13)
C(37)-C(38)-H(38)	119.4
C(39)-C(38)-H(38)	119.4
C(38)-C(39)-C(45)	118.38(12)
C(38)-C(39)-C(40)	123.39(13)
C(45)-C(39)-C(40)	118.22(12)
N(5)-C(40)-C(41)	122.36(12)
N(5)-C(40)-C(39)	118.09(12)
C(41)-C(40)-C(39)	119.55(13)
C(40)-C(41)-C(42)	120.56(13)
C(40)-C(41)-H(41)	119.7
C(42)-C(41)-H(41)	119.7
C(43)-C(42)-C(41)	121.69(13)
C(43)-C(42)-H(42)	119.2
C(41)-C(42)-H(42)	119.2
C(42)-C(43)-C(45)	119.06(13)
C(42)-C(43)-C(44A)	118.8(4)
C(45)-C(43)-C(44A)	120.9(5)
C(42)-C(43)-C(44)	120.5(2)
C(45)-C(43)-C(44)	119.6(2)
C(43)-C(45)-C(35)	120.17(13)
C(43)-C(45)-C(39)	120.82(12)
C(35)-C(45)-C(39)	119.00(13)
N(6)-C(46)-C(47)	122.35(13)
N(6)-C(46)-H(46)	118.8
C(47)-C(46)-H(46)	118.8
C(52)-C(47)-C(48)	118.74(12)
C(52)-C(47)-C(46)	118.13(12)
C(48)-C(47)-C(46)	123.12(12)
C(49)-C(48)-C(47)	120.47(12)
C(49)-C(48)-H(48)	119.8
C(47)-C(48)-H(48)	119.8
C(48)-C(49)-C(50)	121.14(13)
C(48)-C(49)-H(49)	119.4
C(50)-C(49)-H(49)	119.4
C(51)-C(50)-C(49)	117.58(12)
C(51)-C(50)-B(2)	118.97(12)
C(49)-C(50)-B(2)	123.45(12)
C(52)-C(51)-C(50)	121.51(12)
C(52)-C(51)-H(51)	119.2
C(50)-C(51)-H(51)	119.2
C(51)-C(52)-C(47)	120.55(13)
C(51)-C(52)-H(52)	119.7
C(47)-C(52)-H(52)	119.7
O(7)-C(53)-C(54)	108.24(13)

O(7)-C(53)-C(55)	105.96(14)
C(54)-C(53)-C(55)	110.51(15)
O(7)-C(53)-C(56)	102.47(11)
C(54)-C(53)-C(56)	114.32(15)
C(55)-C(53)-C(56)	114.49(15)
C(53)-C(54)-H(54A)	109.5
C(53)-C(54)-H(54B)	109.5
H(54A)-C(54)-H(54B)	109.5
C(53)-C(54)-H(54C)	109.5
H(54A)-C(54)-H(54C)	109.5
H(54B)-C(54)-H(54C)	109.5
C(53)-C(55)-H(55A)	109.5
C(53)-C(55)-H(55B)	109.5
H(55A)-C(55)-H(55B)	109.5
C(53)-C(55)-H(55C)	109.5
H(55A)-C(55)-H(55C)	109.5
H(55B)-C(55)-H(55C)	109.5
O(8)-C(56)-C(57)	106.41(12)
O(8)-C(56)-C(58)	109.38(12)
C(57)-C(56)-C(58)	110.55(14)
O(8)-C(56)-C(53)	102.43(11)
C(57)-C(56)-C(53)	113.77(14)
C(58)-C(56)-C(53)	113.65(14)
C(56)-C(57)-H(57A)	109.5
C(56)-C(57)-H(57B)	109.5
H(57A)-C(57)-H(57B)	109.5
C(56)-C(57)-H(57C)	109.5
H(57A)-C(57)-H(57C)	109.5
H(57B)-C(57)-H(57C)	109.5
C(56)-C(58)-H(58A)	109.5
C(56)-C(58)-H(58B)	109.5
H(58A)-C(58)-H(58B)	109.5
C(56)-C(58)-H(58C)	109.5
H(58A)-C(58)-H(58C)	109.5
H(58B)-C(58)-H(58C)	109.5
O(9)-S(1)-C(62)	106.39(10)
O(9)-S(1)-C(61)	104.64(10)
C(62)-S(1)-C(61)	97.33(11)
S(1)-C(62)-H(62A)	109.5
S(1)-C(62)-H(62B)	109.5
H(62A)-C(62)-H(62B)	109.5
S(1)-C(62)-H(62C)	109.5
H(62A)-C(62)-H(62C)	109.5
H(62B)-C(62)-H(62C)	109.5
O(9)-S(1A)-C(62A)	98.5(17)
O(9)-S(1A)-C(61)	100.2(4)
C(62A)-S(1A)-C(61)	86.1(19)
S(1A)-C(62A)-H(62D)	109.5
S(1A)-C(62A)-H(62E)	109.5
H(62D)-C(62A)-H(62E)	109.5

S(1A)-C(62A)-H(62F)	109.5
H(62D)-C(62A)-H(62F)	109.5
H(62E)-C(62A)-H(62F)	109.5
S(1)-C(61)-H(61A)	109.5
S(1)-C(61)-H(61B)	109.5
H(61A)-C(61)-H(61B)	109.5
S(1)-C(61)-H(61C)	109.5
H(61A)-C(61)-H(61C)	109.5
H(61B)-C(61)-H(61C)	109.5
S(1A)-C(61)-H(61D)	109.5
S(1A)-C(61)-H(61E)	109.5
H(61D)-C(61)-H(61E)	109.5
S(1A)-C(61)-H(61F)	109.5
H(61D)-C(61)-H(61F)	109.5
H(61E)-C(61)-H(61F)	109.5
O(10)-S(2)-C(64)	105.9(3)
O(10)-S(2)-C(63)	104.5(2)
C(64)-S(2)-C(63)	99.3(2)
S(2)-C(64)-H(64A)	109.5
S(2)-C(64)-H(64B)	109.5
H(64A)-C(64)-H(64B)	109.5
S(2)-C(64)-H(64C)	109.5
H(64A)-C(64)-H(64C)	109.5
H(64B)-C(64)-H(64C)	109.5
O(10A)-S(2A)-C(64A)	102.7(9)
O(10A)-S(2A)-C(63)	106.3(9)
C(64A)-S(2A)-C(63)	97.7(6)
S(2A)-C(64A)-H(64D)	109.5
S(2A)-C(64A)-H(64E)	109.5
H(64D)-C(64A)-H(64E)	109.5
S(2A)-C(64A)-H(64F)	109.5
H(64D)-C(64A)-H(64F)	109.5
H(64E)-C(64A)-H(64F)	109.5
S(2)-C(63)-H(63A)	109.5
S(2)-C(63)-H(63B)	109.5
H(63A)-C(63)-H(63B)	109.5
S(2)-C(63)-H(63C)	109.5
H(63A)-C(63)-H(63C)	109.5
H(63B)-C(63)-H(63C)	109.5
S(2A)-C(63)-H(63D)	109.5
S(2A)-C(63)-H(63E)	109.5
H(63D)-C(63)-H(63E)	109.5
S(2A)-C(63)-H(63F)	109.5
H(63D)-C(63)-H(63F)	109.5
H(63E)-C(63)-H(63F)	109.5

8. References

1. CrysAlisPro 1.171.39.46 (Rigaku Oxford Diffraction, 2018).
2. SHELXL: G. M. Sheldrick, ActaCryst, 2015, C71, 3-8.
3. ShelXle: a Qt graphical user interface for SHELXL: C. B. Hübschle, G. M. Sheldrick and B. Dittrich, J. Appl. Cryst., 44, (2011) 1281-1284.
4. ORTEP3 for Windows L. J. Farrugia, J. Appl. Crystallogr. 1997, 30, 565.
5. Mercury: C. F. Macrae, P. R. Edgington, P. McCabe, E. Pidcock, G. P. Shields, R. Taylor, Towler and van der Streek, J. Appl. Crystallogr., 2006, 39, 453-457.

6. Frisch, M. J. et al.; Gaussian 09 Gaussian Inc., Wallingford, CT (2009)
7. Perdew, J. P.; Burke, K.; Ernzerhof, M., Generalized Gradient Approximation Made Simple. *Physical Review Letters* 1996, 77 (18), 3865-3868.
8. Mulliken, R. S., Electronic Population Analysis on LCAO–MO Molecular Wave Functions. I. *The Journal of Chemical Physics* 1955, 23 (10), 1833-1840.
9. GaussView, Version 6.1.1, Roy Dennington, Todd Keith, and John Millam, Semichem Inc., Shawnee Mission, KS, 2019
10. Su-Ying Xu, Xiaolong Sun, Haobo Ge, Rory L. Arrowsmith, John S. Fossey, Sofia I. Pascu, Yun-Bao Jiang and Tony D. James, *Synthesis and evaluation of a boronate-tagged 1,8-naphthalimide probe for fluoride recognition*, Org. Biomol. Chem., 2015, 13, 4143
